# Structure-guided and phage-assisted evolution of a therapeutic anti-EGFR antibody to reverse acquired resistance

**DOI:** 10.1038/s41467-022-32159-6

**Published:** 2022-07-30

**Authors:** Xinlei Zhuang, Zhe Wang, Jiansheng Fan, Xuefei Bai, Yingchun Xu, James J. Chou, Tingjun Hou, Shuqing Chen, Liqiang Pan

**Affiliations:** 1 Institute of Drug Metabolism and Pharmaceutical Analysis, College of Pharmaceutical Sciences, Zhejiang University, Hangzhou, 310058 China; 2 Department of Biological Chemistry and Molecular Pharmacology, Harvard Medical School, 250 Longwood Avenue, Boston, MA 02115 USA; 3 Department of Precision Medicine on Tumor Therapeutics, ZJU-Hangzhou Global Scientific and Technological Innovation Center, Hangzhou, 311200 China; 4 The First Affiliated Hospital, Zhejiang University School of Medicine, Hangzhou, 310003 China; 5 Key Laboratory of Pancreatic Disease of Zhejiang Province, Hangzhou, 310003 China

**Keywords:** Molecular evolution, Antibody therapy, Protein design

## Abstract

Acquired resistance to cetuximab in colorectal cancers is partially mediated by the acquisition of mutations located in the cetuximab epitope in the epidermal growth factor receptor (EGFR) ectodomain and hinders the clinical application of cetuximab. We develop a structure-guided and phage-assisted evolution approach for cetuximab evolution to reverse EGFR^S492R^- or EGFR^G465R^-driven resistance without altering the binding epitope or undermining antibody efficacy. Two evolved cetuximab variants, Ctx-VY and Ctx-Y104D, exhibit a restored binding ability with EGFR^S492R^, which harbors the most common resistance substitution, S492R. Ctx-W52D exhibits restored binding with EGFR harboring another common cetuximab resistance substitution, G465R (EGFR^G465R^). All the evolved cetuximab variants effectively inhibit EGFR activation and downstream signaling and induce the internalization and degradation of EGFR^S492R^ and EGFR^G465R^ as well as EGFR^WT^. The evolved cetuximab variants (Ctx-VY, Ctx-Y104D and Ctx-W52D) with one or two amino acid substitutions in the complementarity-determining region inherit the optimized physical and chemical properties of cetuximab to a great extent, thus ensuring their druggability. Our data collectively show that structure-guided and phage-assisted evolution is an efficient and general approach for reversing receptor mutation-mediated resistance to therapeutic antibody drugs.

## Abstract (web summary)

Acquired resistance to cetuximab can be mediated by generation of mutations in the EGFR ectodomain. Here the authors report a structure-guided and phage-assisted evolution approach for cetuximab evolution to reverse resistance without altering the binding epitope or undermining antibody efficacy.

## Introduction

Since Muromonab-CD3, marketed under the name Orthoclone OKT3, was approved by the FDA in 1986 for the treatment of acute organ transplant rejection^1^, monoclonal antibodies (mAbs) have been widely used to treat various diseases, such as cancers, immune disorders, and infections^2–4^. However, the emergence of primary (de novo) or secondary (acquired) resistance with different underlying mechanisms, mainly but not limited to antigen point mutation-mediated drug resistance^5–7^, aberrancies in downstream signaling pathways^8–10^ and upregulated expression of drug resistance-related proteins^11,12^, undermines the application of therapeutic antibodies for targeted cancer therapy.

Cetuximab, a human-mouse chimeric IgG1 mAb targeting epidermal growth factor receptor (EGFR), is the first mAb drug approved for treating patients with irinotecan-refractory and/or oxaliplatin-refractory metastatic colorectal cancer (mCRC)^13^. Panitumumab, a fully human IgG2 mAb, is another EGFR-targeted therapeutic mAb for mCRC^14^. Both cetuximab and panitumumab compete with epidermal growth factor (EGF) for binding to perturb the downstream signaling of EGFR, thus inhibiting the proliferation of tumor cells^15,16^. The point mutation T790M, an acquired mutation located in the intracellular domain of EGFR, is the most commonly associated with intrinsic resistance as for acquired resistance to EGFR tyrosine kinase inhibitors (TKIs)^17,18^, whereas point mutations in the EGFR ectodomain (ECD) are inclined to disrupt the specific targeting of therapeutic antibodies^19^. Analysis of clinical cell-free DNA (cfDNA) from mCRC patients with a history of anti-EGFR mAb treatment revealed a cluster of EGFR ECD mutations that mediate acquired resistance by attenuating anti-EGFR antibody binding, for example, EGFR ECD mutations at positions V441, S442, R451, I462, S464, G465, K467, K489, I491 and S492^20–26^. All the above residues except R451 are located in the binding site of cetuximab and/or panitumumab^24^. S492R and G465R accounted for approximately 20% of clinical acquired resistance cases, and are among the most common point mutations conferring resistance to anti-EGFR antibodies^20,21^. Strategies for reversing point mutation-driven resistance to anti-EGFR antibodies have focused on the employment of new antibody combinations, e.g., MM-151 and Sym004, to target nonoverlapping epitopes, a time-consuming and labor-intensive approach^26,27^.

In this study, we provide a structure-guided and phage-assisted evolution (SGAPAE) approach for the evolution of the parental antibody cetuximab to reverse resistance mediated by EGFR^S492R^ or EGFR^G465R^, the two most common antibody-resistance point mutations in cetuximab treatment. We use the SGAPAE approach to determine the energy difference between the bound and unbound states of the interface using the Rosetta platform, developing a semirationally designed library of cetuximab mutants with a restricted epitope. The focused library allows us to efficiently identify three cetuximab variants with minimal mutations (Ctx-VY, Ctx-Y104D and Ctx-W52D) to reverse EGFR^S492R^ or EGFR^G465R^-driven resistance to cetuximab, while maintaining the optimized physical and chemical properties of cetuximab to a great extent and thus ensuring their druggability. The SGAPAE approach thus constitutes an efficient and viable strategy to overcome single mutation-driven drug resistance while maintaining the druggability of the parental antibodies, and should be a promising approach for the evolution of other biologics.

## Results

### Many acquired EGFR mutations block cetuximab and/or panitumumab binding and confer drug resistance

**Fig. 1:**
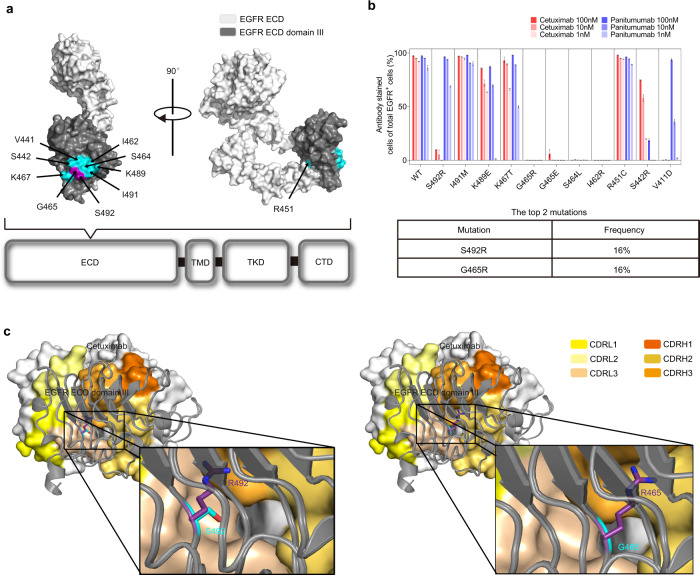
The impact of acquired point mutations in the EGFR ectodomain on the binding ability of cetuximab. **a** The location of acquired point mutations in EGFR. The structure of domain III (dark gray) in the EGFR ECD (white) was adapted from the Protein Data Bank (PDB accession code: 1YY9) and visualized by PyMOL. Single amino acid mutations that abrogate cetuximab or panitumumab binding are highlighted in cyan, except the S492R and G465R substitutions (purple). **b** The impact of acquired point mutations in the EGFR ECD on the binding ability of cetuximab and panitumumab (top panel). NIH3T3 cells stably expressing full-length EGFR^WT^ or EGFR^Mut^ were treated with cetuximab (red) or panitumumab (blue) (1, 10 and 100 nmol/L) and further fluorescently labeled with a FITC-conjugated goat anti-human IgG (H + L) secondary antibody. Experiments were performed in triplicate. The bars indicate the mean ± SD values. S492R and G465R are the two most frequent mutations, according to the literature (bottom panel). **c** The reported crystal structure of the wild-type EGFR ECD/cetuximab Fab complex (PDB code: 1YY9) and the predicted structure of cetuximab in complex with EGFR^S492R^ or EGFR^G465R^. Source data are provided as a Source Data file.

Recent studies have shown that a number of EGFR ECD mutations may be acquired in patients with metastatic colorectal cancer (mCRC) after cetuximab and/or panitumumab treatment. These EGFR ECD mutations occurred in EGFR domain III (amino acids 334–504) which is the binding epitope for cetuximab and panitumumab (Fig. 1a), mediating acquired resistance to cetuximab and/or panitumumab through attenuating or abolishing antibody binding^20–26^.

To better investigate the effect of these mutations on cetuximab and/or panitumumab, we evaluated the binding of cetuximab and panitumumab (1, 10, and 100 nmol/L) with NIH3T3 cells expressing wild-type EGFR or mutant EGFR (Supplementary Fig. 1) by flow cytometry. Several introduced single mutations nearly abolished anti-EGFR antibody binding to EGFR—e.g., S492R, G465R, S464L or V441D mutation significantly reduced the cetuximab binding affinity, and G465R, G465E, S464L or S442R mutation abolished panitumumab binding to EGFR (Fig. 1b, top). Among the mutations in mCRC patients with a history of cetuximab or panitumumab treatment, S492R and G465R appeared to be the two most common point mutations, present in ~20% of patients with clinical resistance^20,21^. A retrospective analysis of plasma samples from a phase III trial (ASPECCT) in a large number of colorectal cancer patients revealed that the EGFR S492R mutation developed in 16% of patients in the cetuximab arm (Fig. 1b, bottom)^20^.

High-resolution EGFR/cetuximab structures show a large, flat binding interface primarily comprising CDRL3, CDRH3, and CDRH2 of cetuximab^28^. Both the S492R and G465R mutations are located in the cetuximab binding site (epitope) on EGFR. We performed homology modeling to predict the effect of the S492R and G465R mutations on cetuximab binding to EGFR (Fig. 1c). The crystal structure of the wild-type EGFR/Fab complex for cetuximab (PDB code: 1YY9) revealed that the CDR of cetuximab formed a central cavity. The S492 and G465 residues of EGFR are accommodated into the above central cavity^29^. According to our structural modeling results, the mutated arginine (R492 and R465) with a large side chain (guanidino group), however, could not fit into the small central cavity in the cetuximab CDR, thus resulting in steric clashes between cetuximab and EGFR^S492R^ or EGFR^G465R^.

### Application of the SGAPAE approach efficiently identified cetuximab variants to reverse the resistance conferred by EGFR^S492R^ or EGFR^G465R^

**Fig. 2:**
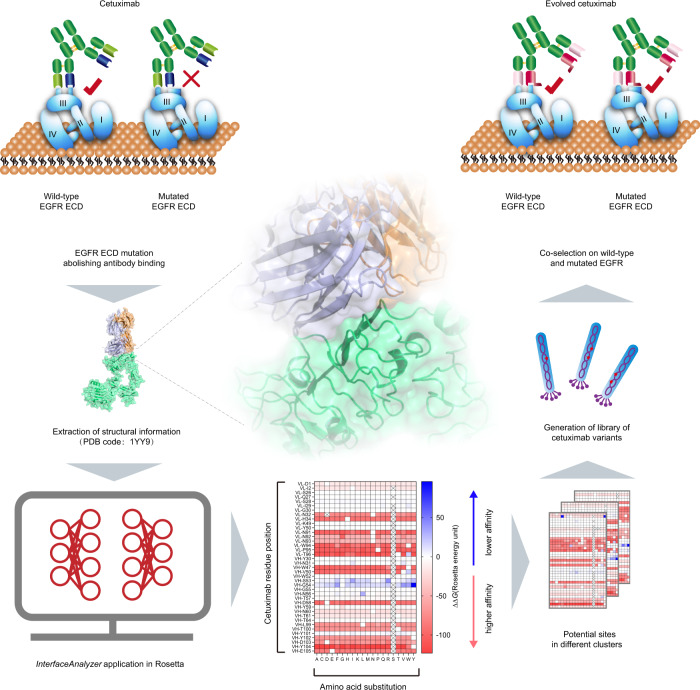
Schematic diagram of the SGAPAE approach for directed evolution of cetuximab. The structure-guided and phage-assisted evolution (SGAPAE) approach was applied to develop cetuximab variants for reversing S492R- or G465R-mediated resistance. First, the crystal structure of the wild-type EGFR/cetuximab Fab complex was obtained from the Protein Data Bank (PDB code: 1YY9). Second, the critical residues in the interface were identified by evaluating the interfacial free energy of the known structure of the EGFR/cetuximab Fab complex and determining the energy difference between the bound and unbound states of the interface with the Rosetta platform. Third, an epitope-restricted cetuximab mutant phage-display library with a size of 10^6^–10^7^ phages was generated by randomization of identified critical residues (*n* = 6). Finally, the cetuximab variants that bound to both EGFR^WT^ and EGFR^Mut^ were selected after panning the cetuximab mutant phage-display library.

We used the SGAPAE approach to direct the evolution of cetuximab to reverse EGFR^S492R^- or EGFR^G465R^-mediated resistance (Fig. 2). Based on the crystal structure of wild-type EGFR bound to cetuximab Fab (PDB code: 1YY9), the Rosetta platform^30^ determined crucial interface residues that are essential for re-establishing the EGFR^S492R^/cetuximab or EGFR^G465R^/cetuximab interaction through an exhaustive scan of residues on the interface. In this way, we constructed an epitope-focused cetuximab mutant library to improve the efficiency of subsequent phage display.

**Fig. 3:**
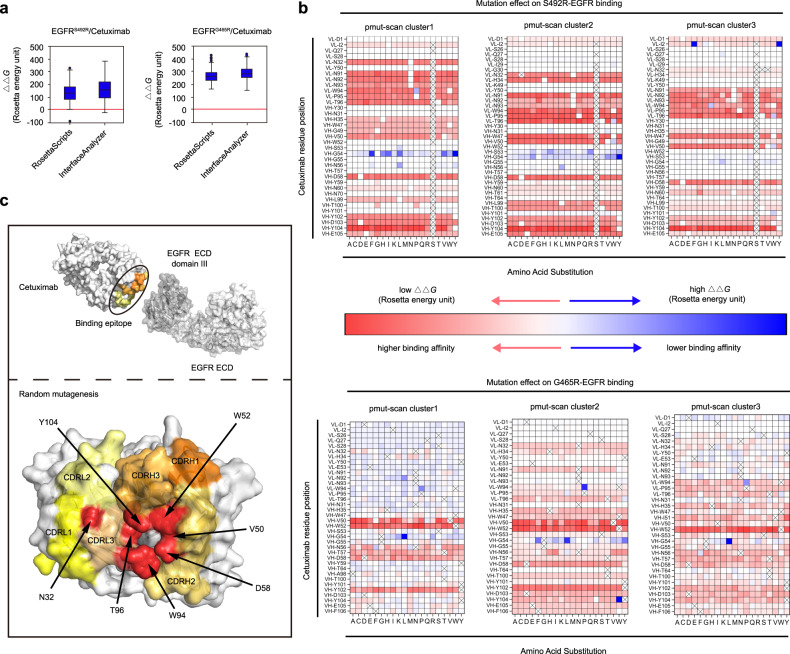
In-silico identification of residues essential for restoration of cetuximab binding. **a** The changes in binding energies (*∆∆G*) caused by the EGFR S492R or G465R mutation were analyzed by RosettaScripts and InterfaceAnalyzer (*n* = 100). The middle line in the boxplot displays the median, the box indicates the first and third quartile, whiskers the 1.5 interquartile range (IQR). **b** The *∆∆G* values for the cetuximab variants in complex with EGFR^S492R^ or EGFR^G465R^ in three clusters (Supplementary Data 1–6). **c** Key amino acid residues are marked in red and shown as spheres at the interface for randomization to construct the cetuximab mutant phage-display library. Source data are provided as a Source Data file.

To facilitate the construction of the focused phage-display library, we used the RosettaScripts protocol^31,32^ and the InterfaceAnalyzer^33^ module in Rosetta^30^, software designed for protein–protein interaction (PPI) interface analysis, to narrow down the list of residues critical for restoring cetuximab binding with the EGFR mutants. Among the multiple indicators (e.g., dG_separated, packstat), dG_separated, which represents the energy difference between protein interface separation and binding, was chosen to evaluate the impact of mutations on cetuximab affinity. EGFR S492R or G465R mutation significantly decreased the binding affinity of cetuximab to EGFR, resulting from an increased free energy of binding. The predicted *∆∆G* values for 100 models of EGFR^S492R^/cetuximab and EGFR^G465R^/cetuximab complexes were consistent with the clinical data (*∆∆G* > 0) (Supplementary Table 1), which justified the application of the RosettaScripts protocol and InterfaceAnalyzer method for predicting changes in binding energy accompanying EGFR mutations (Fig. 3a). We further chose the InterfaceAnalyzer method to perform subsequent mutation scanning for the interface residues of cetuximab because it is easy to use and has high calculation efficiency.

A total of 2014 and 1957 predictions were conducted for the EGFR^S492R^/cetuximab (Cluster 1:684, Cluster 2:665, Cluster 3:665) and EGFR^G465R^/cetuximab (Cluster 1:684, Cluster 2:627, Cluster 3:646) complexes, respectively (Fig. 3b). According to the changes in binding energy (*∆∆G*) in the three clusters (Supplementary Tables 2–7; Supplementary Data 1–6), the intersection of the three clusters, indicating crucial residues for affinity restoration, was obtained. Light chain residues (N32, W94 and T96) and heavy chain residues (V50, D58 and Y014) of cetuximab were identified as potential “replaceable” residues for directed evolution of cetuximab toward binding with EGFR S492R. The heavy chain residues V50 and W52 of cetuximab were identified as mutation site candidates to reverse cetuximab resistance conferred by the acquired EGFR G465R mutation. Therefore, we selected the above potent residues in the receptor binding site of cetuximab for further construction of the phage-display mutagenesis library (Fig. 3c).

**Fig. 4:**
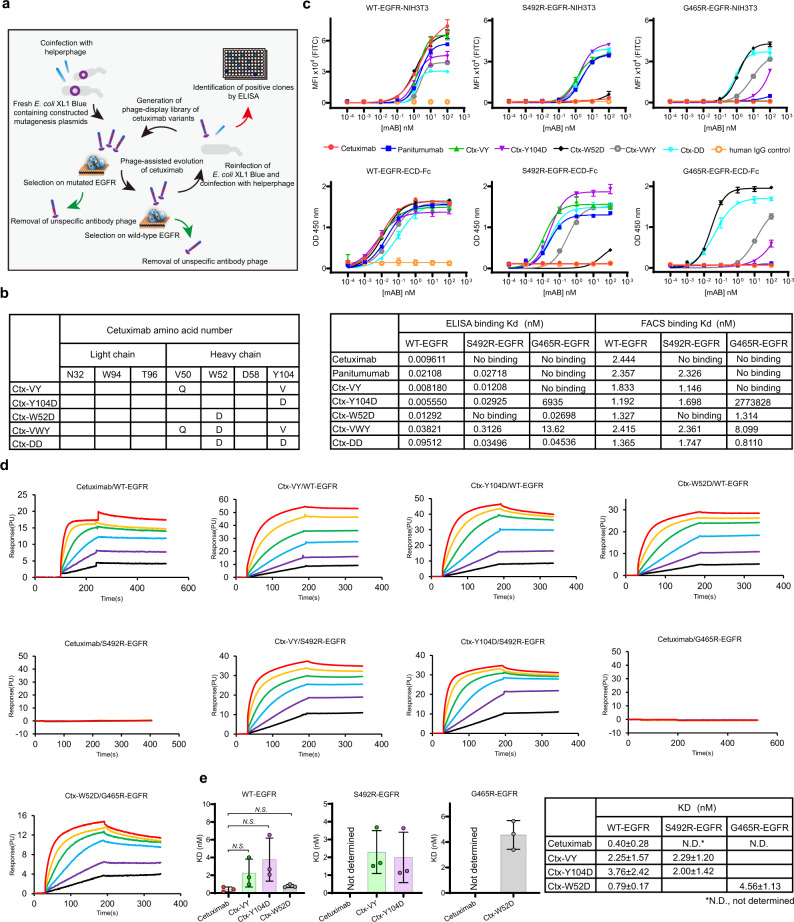
Identification and characterization of the evolved cetuximab variants. **a** Schematic diagram of phage-assisted cetuximab evolution. **b** Summary of the mutations responsive to directed evolution of cetuximab to reverse EGFR^S492R^- or EGFR^G465R^- mediated resistance. **c** The binding ability of cetuximab and its variants with (top panel) NIH3T3 cells expressing EGFR^WT^ or EGFR^Mut^ (i.e., EGFR^S492R^ and EGFR^G465R^) or with (bottom panel) the WT-EGFR-ECD-Fc or Mut-EGFR-ECD-Fc fusion protein. NIH3T3 cell-based binding assays were performed by flow cytometry, while EGFR-ECD-Fc fusion protein-related binding assays were performed by ELISA. The Kd value was calculated using nonlinear regression analysis of a one-site binding hyperbolic equation in GraphPad Prism 8.0 software. **d** Representative traces of SPR analysis of the binding kinetics of the cetuximab variants with WT-EGFR-ECD-Fc or Mut-EGFR-ECD-Fc. **e** The determination of dissociation constants (KD) of cetuximab and its variants upon binding with the fusion protein WT-EGFR-ECD-Fc or Mut-EGFR-ECD-Fc. All above assays were performed in triplicate. The bars indicate the mean ± SD values (*N.S.* means not significant; two-tailed Student’s *t*-test). Source data are provided as a Source Data file.

We applied a phage-display-based coselection strategy^34,35^ to screen for cetuximab scFv mutants that can bind EGFR^G465R^ or EGFR^S492R^ while maintaining high affinity for wild-type EGFR (Fig. 4a). After three rounds of panning, two cetuximab scFv variants targeting EGFR^S492R^ were identified from the S492R library and named Ctx-VY-scFv (V50Q, CDRH2; Y104V, CDRH3) and Ctx-Y104D-scFv (Y104D, CDRH3) (Fig. 4b). Another cetuximab variant targeting EGFR^G465R^ was selected from the G465R library and named Ctx-W52D-scFv (W52D, CDRH3) (Fig. 4b). For further characterization, Ctx-VY-scFv, Ctx-Y104D-scFv and Ctx-W52D-scFv were transformed into human IgG1/κ format (Ctx-VY, Ctx-Y104D and Ctx-W52D) and were then transiently expressed in HEK293F cells. The heavy and light chains of each cetuximab variant appeared at approximately 55 and 30 kDa, respectively, under reducing conditions, and the intact cetuximab variants appeared at approximately150 kDa (Supplementary Fig. 2a). The SDS–PAGE results, together with the size-exclusion chromatography (SEC) results and dynamic light scattering (DLS) results, indicated that the introduced mutations did not affect the stability or polymerization state of the parental antibody (Supplementary Fig. 2a–c). On this basis, we further combined the W52D mutation with the V50Q/Y104V or Y104D mutations to generate cetuximab variants Ctx-VWY (V50Q and W52D, CDRH2; Y104V, CDRH3) and Ctx-DD (W52D, CDRH2; Y104D, CDRH3) in order to cover these most common EGFR mutations (Fig. 4b and Supplementary Fig. 3a).

### Cetuximab variants restored binding to EGFR^G465R^ or EGFR^S492R^

The binding of Ctx-VY, Ctx-Y104D, Ctx-W52D, Ctx-VWY and Ctx-DD to wild-type EGFR or EGFR mutants was analyzed by flow cytometry and ELISA. Panitumumab, wild-type and mutant EGFR-ECD-Fc, and cetuximab variants were successfully produced in HEK293F cells and demonstrated to be intact and correctly assembled according to the SDS–PAGE results under reducing and nonreducing conditions (Supplementary Fig. 3b, c). As shown in Fig. 4c, cetuximab, panitumumab, Ctx-VY and Ctx-W52D showed a similar binding affinity with wild-type EGFR-expressing stable NIH3T3 (WT-EGFR-NIH3T3) cells and WT-EGFR-ECD-Fc. The introduction of Y104D slightly weakened the binding ability of cetuximab with either wild-type EGFR-expressing stable NIH3T3 cells or WT-EGFR-ECD-Fc in comparison with that of the other evolved mutants Ctx-VY and Ctx-W52D. Furthermore, when two mutations, Y104D and W52D (Ctx-DD), or three mutations, V50Q, W52D and Y104V (Ctx-VWY), were introduced at the same time, the binding affinity of the mutants, especially Ctx-DD, for WT-EGFR-NIH3T3 and WT-EGFR-ECD-Fc was greatly reduced. As expected, cetuximab did not exhibit binding with EGFR^S492R^-expressing stable NIH3T3 cells (S492R-EGFR-NIH3T3), while Ctx-VY, Ctx-Y104D, Ctx-DD and Ctx-VWY exhibited successfully restoration of a strong interaction with S492R-EGFR-NIH3T3 cells similar to that of panitumumab, an anti-EGFR antibody with a binding site different from that of cetuximab. Intriguingly, Y104D resulted in a higher Emax with EGFR^S492R^-expressing stable NIH3T3 cells and S492R-EGFR-ECD-Fc than panitumumab, Ctx-VY, Ctx-DD and Ctx-VWY. Among all the cetuximab variants, only Ctx-W52D exhibited restoration of strong binding to EGFR-expressing stable cells and for EGFR-ECD-Fc with the G465R mutation, which abolished the binding of both cetuximab and panitumumab. In addition, we tested the binding affinity of Ctx-W52D in the EGFR^G465E^ model. However, Ctx-W52D could not reverse G465E-driven resistance to cetuximab (Supplementary Fig. 4a).

### VH-Y104 of cetuximab is a critical residue for the EGFR S492R mutation

The Y104 residue in the cetuximab VH domain (VH-Y104) is alteredin both Ctx-VY and Ctx-Y104D. Hence, we performed site saturation mutagenesis for VH-Y104 and Ctx-Y104X (X = A, C, D, E, F, G, H, I, K, L, M, N, P, Q, R, S, T, V, W, Y) to thoroughly investigate the impact of this residue on the binding affinity of cetuximab for EGFR^S492R^ (Supplementary Fig. 3d). As shown in Supplementary Fig. 4b, Ctx-Y104D, Ctx-Y104C and Ctx-Y104N are the top three high-affinity binders to EGFR^S492R^ for both stable cells and S492R-EGFR-ECD-Fc, which suggests that the size of the residue side chain is not the major mechanism for restoring EGFR^S492R^ binding. In addition, although Ctx-VY showed favorable binding with EGFR^WT^ and EGFR^S492R^, the binding of Ctx-Y104V to EGFR^S492R^ was weaker than that of the Ctx-Y104D/C/N mutants (Supplementary Fig. 4b). Therefore, we further explored the impact of the V50Q mutation (located in CDRH3) on Ctx-VY binding affinity (Supplementary Fig. 4c). Cetuximab with either Y104V or V50Q showed a decreased affinity for EGFR^S492R^, indicating that both mutations are essential for cetuximab to achieve high-affinity binding with EGFR^WT^ and EGFR^S492R^.

### Cetuximab variants restored high-affinity binding to EGFR^Mut^ while exhibiting binding kinetics similar to those of cetuximab in the interaction with wild-type EGFR

The binding kinetics of cetuximab and the cetuximab variants with WT-EGFR-ECD-Fc and Mut- EGFR-ECD-Fc were quantitively determined by SPR analysis (Fig. 4d and Supplementary Table 8). Cetuximab, Ctx-VY, Ctx-Y104D and Ctx-W52D exhibited similar high-affinity binding to WT-EGFR-ECD-Fc, with *K*_D_ values of 0.40 ± 0.28, 2.25 ± 1.57, 3.76 ± 2.42, and 0.79 ± 0.17 nM, respectively (Fig. 4e). The cetuximab variants had a relatively low association rate (mean value: Ctx-VY: kon = 5.77 × 10^4^ Ms^−1^, koff = 1.29 × 10^−4^ s^−1^; Ctx-Y104D: kon = 8.20 × 10^4^ Ms^−1^, koff = 3.07 × 10^−4^ s^−1^; Ctx-W52D: kon = 3.54 × 10^5^ Ms^−1^, koff = 8.78 × 10^−5^ s^−1^) compared to that of cetuximab (mean value: kon=1.68×10^5^ Ms^−1^, koff = 7.29 × 10^−5^ s^−1^), which accounted for the incomplete restoration of binding affinity for EGFR^WT^. The interaction of cetuximab with EGFR^S492R^ or EGFR^G465R^ was not detectable by SPR; in contrast, Ctx-VY, Ctx-Y104D, and Ctx-W52D, exhibited major restoration of binding with EGFR^S492R^ (*K*_D_ = 2.29 ± 1.20 nM; *K*_D_ = 2.00 ± 1.42 nM) and EGFR^G465R^ (K_D_ = 4.56 ± 1.13 nM) with respect to the cetuximab/EGFR^WT^ interaction (Fig. 4e). The deleterious impact of the S492R and G465R mutations on cetuximab binding affinity was also confirmed by SPR.

### Potential structural basis of the binding mechanism of cetuximab variants to S492R- or G465R-mutated EGFR

**Fig. 5:**
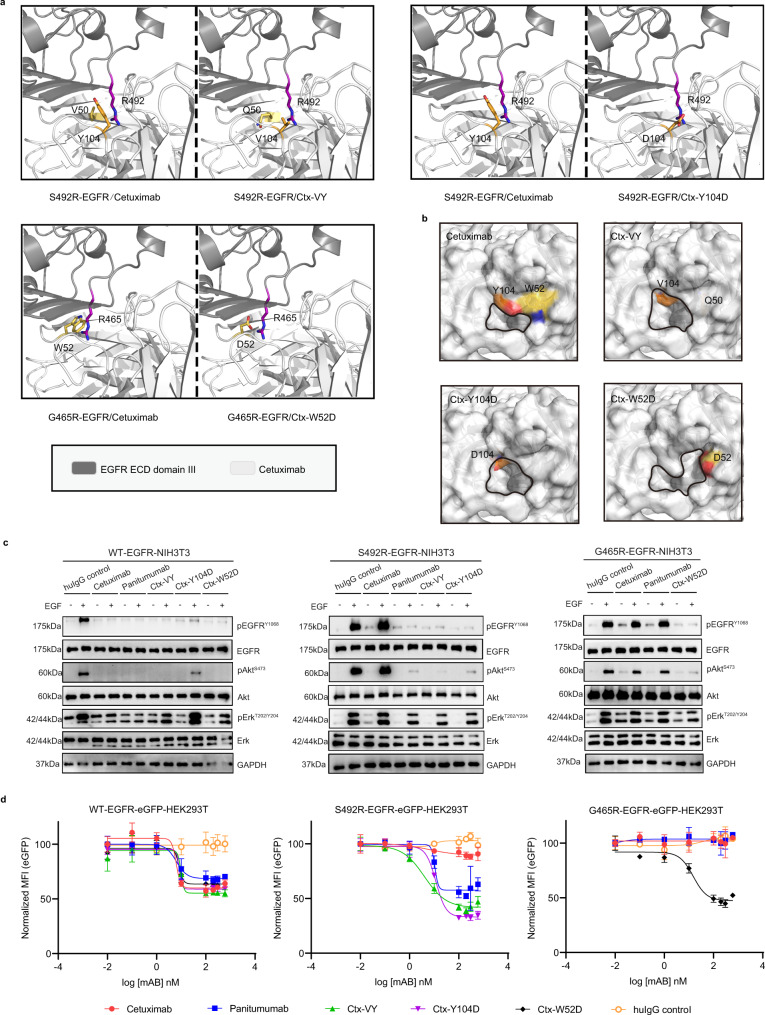
The mechanisms of action by which the cetuximab variants in inhibit EGFR^S492R^ or EGFR^G465R^ activation. **a** The modeled structural mechanisms by which the cetuximab variants restore binding with EGFR^S492R^ or EGFR^G465R^. The critical residues in the cetuximab variants and the EGFR mutations are highlighted as colored sticks in the interface. The structures of VH domain in the cetuximab/cetuximab variants and EGFR^S492R^ or EGFR^G465R^ ECD domain III are shown in white and gray, respectively. **b** The central cavities (circled in black) in the paratopes of cetuximab (PDB code: 1YY9) and its variants. **c** The mechanism by which the cetuximab variants inhibit the ligand-induced activation of EGFR^S492R^ or EGFR^G465R^, as shown by western blot analysis. Stable NIH3T3 cells were stimulated with EGF (0.5 nM) and immunoblotted for total and phosphorylated EGFR, Akt, and Erk (pEGFR^Y1068^, pAkt^S473^, and pErk^T202/Y204^). **d** The mechanism by which the cetuximab variants inhibit the ligand-induced receptor internalization and degradation of EGFR^S492R^ or EGFR^G465R^, as shown by flow cytometry. The mean fluorescence intensity (MFI) of different stable HEK293T cells was determined after 48 h of preincubation with the indicated concentrations of antibodies. MFI values were normalized to the EGFR-eGFP signal in cells with no antibody treatment. All above assays were performed in triplicates. The bars indicate the mean ± SD values. Source data are provided as a Source Data file.

To explore the potential structural basis underlying the binding of the cetuximab variants to EGFR^S492R^ and EGFR^G465R^, we simulated the structure of the cetuximab Fab variants bound to domain III of EGFR with the S492R or G465R substitution via the protein structure homology-modelling server SWISS-MODEL (Fig. 5a). Because of the minimal mutations in cetuximab CDR, the overall structures of the EGFR^S492R^/cetuximab Fab variant complex and EGFR^G465R^/cetuximab Fab variant complex were extremely similar to that of EGFR^WT^ in complex with cetuximab Fab. The restoration of the binding affinity of the cetuximab variants could be attributed to the significant reduction in steric hindrance via the V50Q/Y104V mutation (Ctx-VY) or Y104D mutation (Ctx-Y104D) and possible strong charge accumulation through the Asp-Arg (D-R) salt bridge (Ctx-Y104D versus EGFR^S492R^) (Fig. 5a). Regarding the superiority of Ctx-W52D in recognizing EGFR^G465R^, the Asp-Arg (D-R) salt bridge-mediated reconnection and reduction in steric hindrance probably contributed simultaneously to restoration of the binding ability, since the large indolyl side chain of tryptophan (W) was downsized to the carboxyl group of aspartate (D). We also compared the paratopes of cetuximab and the cetuximab variants in surface mode (Fig. 5b). The size of the central cavity surrounded by CDRL3, CDRH2 and CDRH3 is different between cetuximab and the cetuximab variants. The larger central cavity of the cetuximab variants could accommodate the side chain of mutated R492 or R465, which may explain the molecular mechanism underlying the restoration of cetuximab binding to EGFR^S492R^ and EGFR^G465R^ (Fig. 5b).

### The evolved cetuximab variants successfully inhibited EGF-induced EGFR^S492R^ and EGFR^G465R^ activation

The primary mechanism of action of cetuximab is the blockade of ligand-induced EGFR activation and downstream signaling, including signaling through the PI3K/Akt and MAPK/Erk pathways which have been linked to many processes crucial to tumor progression, including metastasis and cell survival, proliferation, adhesion, differentiation, migration, transformation, and motility^36^. To evaluate the inhibitory activities of the cetuximab variants, we established stable NIH3T3 cells expressing full-length EGFR^WT^ or EGFR^Mut^ as an in vitro cell model. Robust phosphorylation of EGFR and activation of Akt and Erk in the downstream pathway were induced in stable WT-EGFR-NIH3T3, S492R-EGFR-NIH3T3 and G465R-EGFR-NIH3T3 cells upon EGF stimulation, according to the western blot results (Fig. 5c). Pretreatment with cetuximab, panitumumab, Ctx-VY, Ctx-Y104D, or Ctx-W52D reduced the level of phosphorylated EGFR (pEGFR) to basal levels and decreased the levels of phosphorylated Akt and Erk (pAkt and pErk) in WT-EGFR-NIH3T3 cells (Fig. 5c, left). In the S492R-EGFR-NIH3T3 cells, panitumumab, Ctx-VY and Ctx-Y104D but not cetuximab abolished EGF-induced EGFR phosphorylation and downstream signaling (Fig. 5c, middle), which was consistent with the binding affinity results (Fig. 4c). In G465R-EGFR-NIH3T3 cells, Ctx-W52D significantly reduced EGF-stimulated EGFR^G465R^ activation, although cetuximab and panitumumab did not, according to the western blot results for downstream pathway indicators, such as pEGFR, pAkt and pErk (Fig. 5c, right). Owing to the incomplete restoration of the binding affinity of the cetuximab variants for EGFR^WT^, the Erk attenuation induced by the cetuximab variants was still limited, particularly in WT-EGFR-NIH3T3 cells.

Induction of EGFR internalization and degradation, which results in reduced EGFR downstream signaling, is another mechanism of cetuximab^37^. HEK293T cell lines stably expressing various full-length EGFR-eGFP fusion proteins were successfully generated and named WT-EGFR-eGFP-HEK293T, S492R-EGFR-eGFP-HEK293T and G465R-EGFR-eGFP-HEK293T (Supplementary Fig. 5a, b). We used flow cytometry to measure the EGFR-eGFP signal in HEK293T cells after 48 h of antibody exposure (Fig. 5d). In WT-EGFR-eGFP-HEK293T cells, cetuximab, panitumumab, Ctx-VY, Ctx-Y104D and Ctx-W52D reduced the eGFP signal (EGFR^WT^-eGFP) in a concentration-dependent manner, with EC50 values of 6.2, 6.3, 9.7, 8.3 and 8.1 nM, respectively (Fig. 5d, left). In S492R-EGFR-eGFP-HEK293T cells, cetuximab showed barely any efficacy in decreasing the eGFP signal (EGFR^S492R^-eGFP), whereas panitumumab, Ctx-VY and Ctx-Y104D induced the degradation of S492R-mutated EGFR with EC50 values of 10.7, 4.0, and 11.8 nM, respectively (Fig. 5d, middle). Ctx-W52D significantly reduced the eGFP signal (EGFR^G465R^-eGFP), with an EC50 of 15 nM in G465R-EGFR-eGFP-HEK293T cells; However, cetuximab and panitumumab did not (Fig. 5d, right).

### Cetuximab variants with unimpaired effector activities potently inhibited tumor cell proliferation

**Fig. 6:**
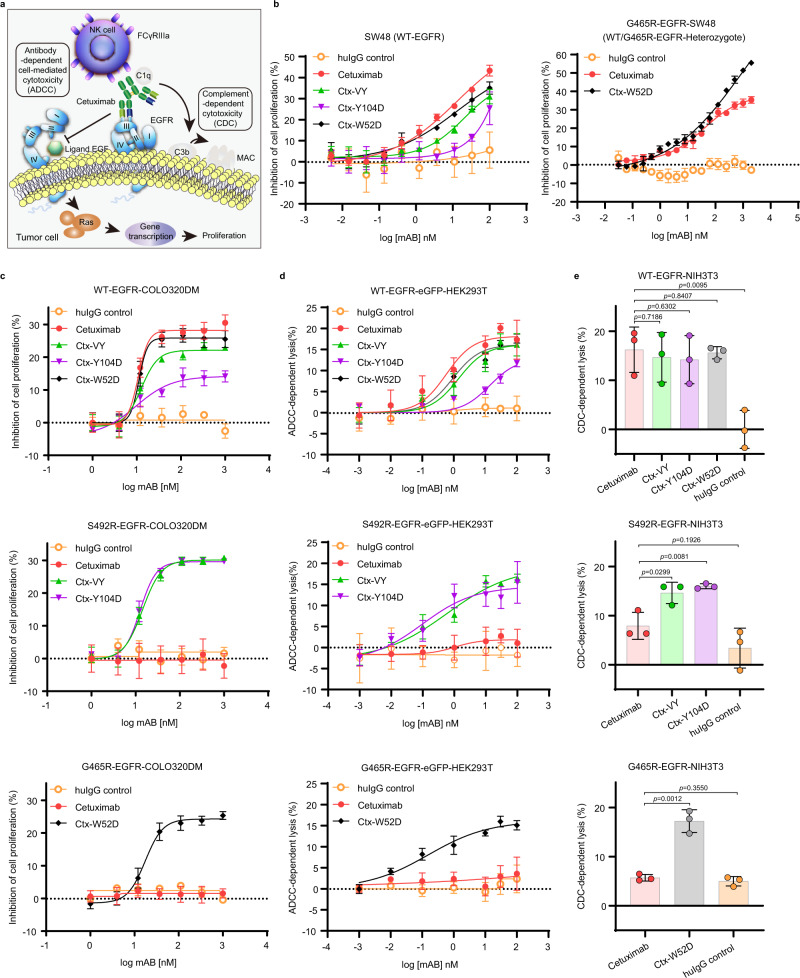
The cetuximab variants inhibited the growth of wild-type EGFR- and S492R/G465R-mutated EGFR-positive cells in vitro. **a** The mechanisms of action underlying the antitumor activities of cetuximab. **b** The in vitro efficacy of cetuximab and the cetuximab variants in inhibiting the proliferation of cetuximab-sensitive/resistant cells. The wild-type EGFR cell line SW48 (WT-EGFR) and SW48 cells stably expressing full-length EGFR^G465R^ (G465R-EGFR-SW48) were used as model cell lines, and treated with serial concentrations of antibodies for 3 days. Cell viability was determined by a CCK-8 assay. **c** The in vitro efficacy of cetuximab and the cetuximab variants in inhibiting the proliferation of cells in the established CRC xenograft model. WT-EGFR-COLO320DM cells, S492R-EGFR-COLO320DM cells and G465R-EGFR-COLO320DM cells were used as model cell lines, and treated with serial concentrations of antibodies for 3 days. Cell viability was determined by a CCK-8 assay. **d** The ADCC activity of cetuximab and the cetuximab variants. LDH release was detected after hours of coincubation of PBMC effector cells and WT-EGFR-eGFP-HEK293T or mutant-EGFR-eGFP-HEK293T target cells at an *E*:*T* ratio of 40:1. **e** The CDC activity of cetuximab and the cetuximab variants. WT-EGFR-NIH3T3 cells and mutant-EGFR-NIH3T3 cells were incubated with serial concentrations of antibodies in the presence of 10% human serum for 2 h, and cell viability was measured by a CCK-8 assay. All above experiments were performed in triplicates. The bars indicate the mean ± SD values (*p < 0.05, **p < 0.01, *N.S.* means not significant; two-tailed Student’s t-test). Source data are provided as a Source Data file.

Cell proliferation inhibition and immune effector activity are important mechanisms of action for antitumor therapeutic antibodies (Fig. 6a). We further evaluated the in vitro efficacy of cetuximab and the cetuximab variants against the wild-type EGFR cell line SW48 (WT-EGFR)^38^, SW48 cells stably expressing full-length EGFR^G465R^, i.e., G465R-EGFR-SW48 (WT/G465R-EGFR-Heterozygote), and COLO320DM cell lines stably expressing full-length wild-type or an EGFR mutant (WT-EGFR-COLO320DM, S492R-EGFR-COLO320DM and G465R-EGFR-COLO320DM) (Fig. 6b, c and Supplementary Fig. 6). COLO320DM is an EGFR-negative human colorectal cancer cell line with wild-type KRAS/BRAF, which is an ideal cell model for generating stable EGFR mutant cell lines. Both cetuximab and the cetuximab variants inhibited the proliferation of SW48 cells and WT-EGFR-COLO320DM cells and exhibited similar in vitro efficacy and wild-type EGFR binding ability (Fig. 6b, left and Fig. 6c, top). Considering the heterogeneous cell surface expression of EGFR and its variants in tumors, we generated the G465R-EGFR-SW48 cell line to evaluate the inhibitory effect of cetuximab and Ctx-W52D. Cetuximab showed lower efficacy in inhibiting EGFR signaling in the G465-EGFR-SW48 model, while Ctx-W52D maintained its high potency. This result indicated that exogenous introduction of full-length EGFR^G465R^ in the SW48 cell model impaired the inhibitory effect of cetuximab but not Ctx-W52D on EGFR downstream signaling (Fig. 6b, right). In the S492R-EGFR-COLO320DM cell model, Ctx-VY and Ctx-Y104D exhibited similar inhibition efficacies; however, cetuximab had almost no inhibitory effect (Fig. 6c, middle). Consistent with its EGFR^G465R^ binding ability, only Ctx-W52D inhibited cell proliferation in a dose-dependent manner in the G465R-EGFR-COLO320DM cell model in comparison with cetuximab and control IgG (Fig. 6c, bottom).

Immune effector activity also plays an important role in the antitumor activity of therapeutic antibodies. We also investigated the complement-dependent cytotoxicity (CDC) and antibody-dependent cellular cytotoxicity (ADCC) activity of cetuximab variants according to previously described methods^39,40^. The results showed that the cetuximab variants exhibited ADCC (Fig. 6d) and CDC (Fig. 6e) activities similar to those of the parental antibody cetuximab in EGFR^WT^ model cells, and showed additional ADCC and CDC activities in EGFR^Mut^-expressing model cells, benefiting from their restored EGFR^Mut^ binding ability.

### Cetuximab variants can inhibit the growth of mutant EGFR-positive and WT-EGFR-positive tumors in vivo

**Fig. 7:**
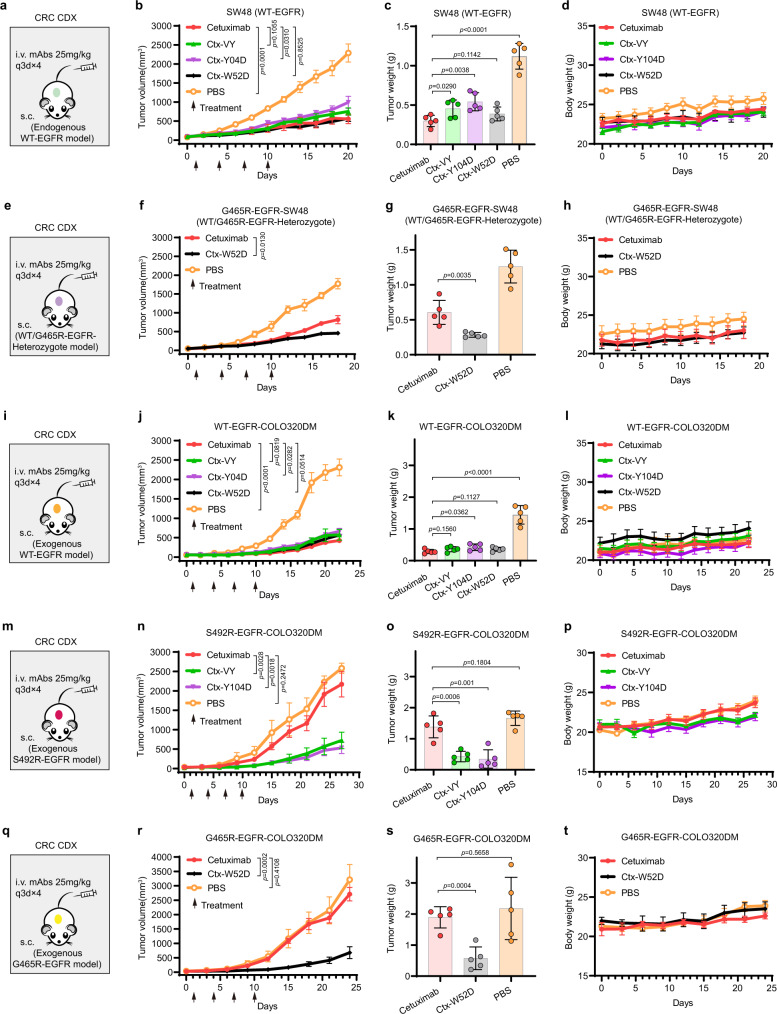
The in vivo antitumor activities of the cetuximab variants in mouse EGFR^WT^- or EGFR^Mut^-positive xenograft models. **a** The illustration of the SW48 (WT-EGFR) xenograft mouse model establishment, and the administration route and antibody dosages. **b** The in vivo efficacy of cetuximab and the cetuximab variants in the SW48 xenograft mouse model. **c** The tumor weight (wet) by the end of study. **d** The monitoring result of mouse body weight in each experimental or control group. **e** The G465R-EGFR-SW48 (WT/G465R-EGFR-Heterozygote) xenograft mouse model. **f** The in vivo efficacy of cetuximab and Ctx-W52D in the G465R-EGFR-SW48 xenograft mouse model. **g** The tumor weight (wet) by the end of study. **h** The monitoring result of mouse body weight. **i** The WT-EGFR-COLO320DM xenograft mouse model. **j** The in vivo efficacy of cetuximab and its variants in the WT-EGFR-COLO320DM xenograft mouse model. **k** The tumor weight (wet) by the end of study. **l** The monitoring result of mouse body weight. **m** The S492R-EGFR-COLO320DM xenograft mouse model. **n** The in vivo efficacy of cetuximab, Ctx-VY and Ctx-Y104D in the S492R-EGFR-COLO320DM xenograft mouse model. **o** The tumor weight (wet) by the end of study. **p** The monitoring result of mouse body weight. **q** The G465R-EGFR-COLO320DM xenograft mouse model. **r** The in vivo efficacy of cetuximab and Ctx-W52D in the G465R-EGFR-COLO320DM xenograft mouse model. **s** The tumor weight (wet) by the end of study. **t** The monitoring result of mouse body weight. For all in vivo experiments, the BALB/c nude mice were subcutaneously injected with tumor cells. When the mean tumor volume reached approximately 50 mm^3^, mice were administered with antibodies at a dosage of 25 mg/kg once every 3 days for four times (q3d×4). All data are shown in mean ± SD values, *n* = 5 mice for each group (*p < 0.05, **p < 0.01, ***p < 0.001, ****p < 0.0001, *N.S.* means not significant; two-tailed Student’s t-test). Source data are provided as a Source Data file.

We used colorectal cancer (CRC) cell line-derived xenograft (CDX) models to evaluate the in vivo antitumor activity of Ctx-VY, Ctx-Y104D and Ctx-W52D. In the SW48 (WT-EGFR) xenograft model (Fig. 7a), tumor growth was significantly suppressed in the Ctx-VY, Ctx-Y104D, Ctx-W52D and cetuximab groups compared to the PBS group, and the in vivo antitumor activities of the cetuximab variants were consistent with their binding affinities for EGFR. In particular, the in vivo performance of Ctx-W52D and cetuximab was highly similar according to the mean tumor volume and tumor weight data (Fig. 7b, c). No body weight loss was observed in mice during cetuximab and cetuximab variants treatment (Fig. 7d). In addition, we could not detect any obvious tissue damage in the important organs, such as heart, liver, spleen, lung, and kidney, in mice, according to histological examination of tissue sections via H&E staining (Supplementary Fig. 7a). Further serological studies revealed that serum ALT, AST, BUN and Cr did not show any significant difference among the vehicle and treatment groups (Supplementary Fig. 7b), excluding the possibility of severe liver or kidney toxicity. In the G465R-EGFR-SW48 (WT/G465R-EGFR-Heterozygote) xenograft model (Fig. 7e), cetuximab could not bind with EGFR^G465R^ or affect EGFR^G465R^ signaling, therefore the exogenous introduction of EGFR G465R in SW48 lowered the potency of cetuximab on tumor growth inhibition in the G465R-EGFR-SW48 (WT/G465R-EGFR-Heterozygote) model, in comparison to the SW48 (WT-EGFR) model. On the other hand, the Ctx-W52D, capable of binding with EGFR^WT^ and EGFR^G465R^, showed similar in vivo efficacy in two xenograft models (Fig. 7f, g). No body weight loss was observed in mice during cetuximab and Ctx-W52D treatment (Fig. 7h). In addition, cetuximab and its variants showed similar in vivo antitumor activity in the WT-EGFR-COLO320DM xenograft model (Fig. 7i), with respect to the SW48 (WT-EGFR) model (Fig. 7j–l). The in vivo efficacy of Ctx-VY and Ctx-Y104D was maintained in the S492R-EGFR-COLO320DM xenograft model; however, there was no significant difference between the cetuximab group and the PBS group in tumor growth suppression (Fig. 7m–o). No body weight loss was observed in mice during Ctx-VY and Ctx-Y104D treatment (Fig. 7p). The in vivo efficacy of Ctx-W52D was also maintained in the G465R-EGFR-COLO320DM xenograft model; however, there was no significant difference between the cetuximab group and PBS group in tumor growth suppression (Fig. 7q–s). No body weight loss was observed in mice during Ctx-W52D treatment (Fig. 7t). Further H&E staining results demonstrated that the tumor tissue morphology was severely disrupted in the cetuximab variant groups compared to the cetuximab group and PBS group (Supplementary Fig. 8a). Immunostaining for EGFR showed that intratumoral EGFR^WT^ expression was downregulated after the treatment of cetuximab and its variants in the EGFR^WT^ model. However, in the EGFR^Mut^ models, immunostaining for EGFR showed that intratumoral EGFR^Mut^ expression was downregulated in the cetuximab variant groups, while no difference was observed between the cetuximab group and vehicle group (Supplementary Fig. 8a). Immunohistochemical staining for Ki-67, an indicator of tumor progression, revealed markedly fewer intratumoral Ki-67-positive cells in cetuximab variant groups, indicating that the cetuximab variants effectively inhibited EGFR^WT^and EGFR^Mut^ tumor cell proliferation in the xenograft model (Supplementary Fig. 8a–d). The TUNEL assay revealed that there were apoptotic tumor cells in cetuximab variant groups, further confirming the in vivo efficacy of the cetuximab variants against EGFR^WT^ and EGFR^Mut^ tumor cells (Supplementary Fig. 8a, e–g).

## Discussion

Owing to deleterious single point mutations in the ectodomain of EGFR, such as S492R and G465R, acquired resistance to anti-EGFR therapeutic antibodies emerges during clinical antibody therapy, hindering further applications of antibody derivatives, including antibody-drug conjugates (ADCs)^41^ and bispecific antibodies (bsAbs)^42^. In this study, we successfully applied a structure-guided and phage-assisted evolution (SGAPAE) approach to direct the evolution of cetuximab, successfully reversing the acquired drug resistance conferred by EGFR ECD mutations. According to structural analysis and computational prediction of EGFR ECD with the S492R mutation, we identified 6 critical residues in the CDRs of cetuximab and created a 6-residue randomized phage-display library. After panning the epitope-restricted cetuximab mutant library, the cetuximab variants Ctx-VY and Ctx-Y104D were identified to exhibit restored binding affinity for EGFR^S492R^ while maintaining their binding affinity for EGFR^WT^. The SGAPAE approach also reversed EGFR^G465R^-driven drug resistance to cetuximab, demonstrating its more general application. Another cetuximab variant, Ctx-W52D, with a single mutation in the cetuximab CDR, was shown to exhibit restored binding affinity for EGFR^G465R^. Intriguingly, all cetuximab variants maintained the functions of their parental antibody cetuximab, including blockade of EGFR activation and downstream signaling, induction of EGFR degradation, inhibition of cell proliferation, and CDC and ADCC activity. Collectively, these results demonstrate that with the SGAPAE approach, we efficiently achieved epitope-restricted evolution of cetuximab and identified cetuximab variants with minimal point mutations (1 or 2) to reverse EGFR^S492R^- or EGFR^G465R^- mediated drug resistance.

The application of SGAPAE in cetuximab evolution, which involves the use of the Rosetta platform, relies heavily on the structural information of the EGFR/cetuximab complex. Developing a computational model consistent with the binding characteristics of an antibody/antigen complex is essential for the prediction of potential hot spot residues for evolution. The Rosetta platform offers several advantages for evaluating the interfacial free energy. The InterfaceAnalyzer module of Rosetta determines the energy difference between the bound and unbound states of the interface, which is beneficial for evaluating the effect of a single point mutation on the affinity of cetuximab based on the known structure of the EGFR/cetuximab Fab complex. The flexible setting of constraints (e.g., residue distance) in the Rosetta platform facilitates fast and exhaustive searching surrounding the point mutation. Finally, with the restricted binding site of the cetuximab variant, Rosetta provides the most accurate local docking pose after simply orienting the Fab in the expected binding site, leading to the identification of key amino acid residues for randomization. Therefore, the Rosetta platform in the SGAPAE approach was ideally suited to address EGFR single mutation (e.g., S492R or G465R)-driven acquired resistance to cetuximab and should be effective for addressing single mutations in other receptors (e.g., HER2 S310F, which abolishes pertuzumab binding)^43^.

To date, phage and yeast display technologies have been widely used in antibody discovery^44^ and cytokine engineering, e.g., IL-2 bias screening^45^. However, to the best of our knowledge, phage display has not been applied to overcome single mutation-mediated antibody-drug resistance. Because of the limited key residues for randomization, the size of the cetuximab mutant library was reduced significantly to 10^6^–10^7^ phages, which exhibited superiority over conventional phage-display libraries with a size of 10^9^–10^12^ phages in biased screening processes ^46^. The diversity of the semirationally designed cetuximab mutant library is confined within the binding epitope of the parental cetuximab structure, providing an obvious evolutionary direction toward EGFR and thereby retaining the highly similar binding site in the selected cetuximab variants. In combination with the known structural information and computational predictions, our SGAPAE approach was able to improve the screening efficiency for effective cetuximab variants. The binding epitope of the parental antibody is usually correlated with antibody efficacy. With the epitope-restricted cetuximab mutant phage-display library, we found that minimal point mutations or even a single point mutation (e.g., Y104D or W52D) in the cetuximab CDR could completely reverse EGFR^S492R^- or EGFR^G465R^- mediated cetuximab resistance without altering the binding epitope or attenuating antibody efficacy. In addition, the evolved cetuximab variants (Ctx-VY, Ctx-Y104D and Ctx-W52D) with one or two amino acid substitutions in the CDR, are less likely than other variants to perturb the rest of the antibody domains, inheriting the optimized physical and chemical properties of cetuximab to the greatest possible extent.

In summary, we successfully applied the SGAPAE approach to efficiently reverse EGFR^S492R^- or EGFR^G465R^- mediated resistance to cetuximab. Acquired drug resistance during antibody therapy has greatly undermined the effectiveness of antibody treatment due to frequent mutations in the target receptors (e.g., EGFR and HER2). The SGAPAE approach provides an efficient and viable strategy to overcome single mutation-driven drug resistance while maintaining the druggability of parental antibodies. The SGAPAE approach also constitutes a promising strategy for efficiently evolution of other proteins, such as recombinant enzymes and cytokines, whose structural information is usually abundant and rapidly expanding owing to the rapid development of structural biology.

## Methods

### Ethical statement

Experiments were carried out according to the National Institutes of Health (United States) Guide for the Care and Use of Laboratory Animals. All animal work was performed in accordance with the protocol approved by the Committee on the Ethics of Animal Experiments of Zhejiang University (Hangzhou, China).

### Cell culture and reagents

NIH3T3 mouse embryonic fibroblasts were obtained from the Cell Bank of the Chinese Academy of Sciences (Shanghai, China). The human colorectal cancer cell lines SW48 and COLO320DM were purchased from Cobioer Biosciences (Nanjing, China). HEK293T cells and HEK293F cells were kindly provided by Professor Dr. Linqi Zhang (Tsinghua University, China). NIH3T3 cells, HEK293T cells, and SW48 cells were cultured in DMEM (Gibco, Grand Island, NY, USA) supplemented with 10% (v/v) fetal bovine serum (Gibco, MA, USA) and 1% (v/v) penicillin/streptomycin (Solarbio, Beijing, China) at 37 °C in a humidified atmosphere containing 5% CO_2_. COLO320DM cells were cultured in RPMI-1640 medium (Gibco, Grand Island, NY, USA) supplemented with 10% (v/v) fetal bovine serum (Gibco, MA, USA) and 1% (v/v) penicillin/streptomycin (Solarbio, Beijing, China) at 37 °C in a humidified atmosphere containing 5% CO_2_. HEK293F cells were cultured in SMM 293-TI medium (SinoBiological, China) supplemented with 0.5% (v/v) fetal bovine serum and 1% (v/v) penicillin/streptomycin at 37 °C under 5% CO_2_ in a New Brunswick S41i shaking incubator (Eppendorf, 120 rpm).

### Mice

BALB/c nude mice (6–8 weeks old, male, body weight 20–30 g) were purchased from Shanghai SLAC Laboratory Animal Co., Ltd. (Shanghai, China) and housed under specific pathogen-free conditions. Experiments were carried out according to the National Institutes of Health (United States) Guide for the Care and Use of Laboratory Animals. All animal work was performed in accordance with the protocol approved by the Committee on the Ethics of Animal Experiments of Zhejiang University (Hangzhou, China).

### Homology modeling and structural analysis

The online server SWISS-MODEL was used to predict the 3D protein structures of S492R- and G465R-mutated EGFR and the cetuximab variants using the crystal structure of the wild-type EGFR/Fab complex for cetuximab (PDB code: 1YY9) as a template. Essential amino acid substitutions that lead to loss of cetuximab or panitumumab binding to EGFR were identified according to the literature (Refs). All predicted structures were decorated and displayed with the open-source version of the PyMOL program (https://github.com/schrodinger/PyMOL -open-source).

### Structure preparation and computational mutation scanning

The crystal structure of the wild-type EGFR ECD/cetuximab Fab complex was obtained from the Protein Data Bank (PDB code: 1YY9). Waters and other HET residues (e.g., NDG, NAG, BMA) in the crystal structure were removed. Missing loops and hydrogen atoms were added to the cleaned structure of the antibody/antigen complex, which was then subjected to initial refinement using the Relax application in Rosetta. The relaxed structure with the lowest energy was selected for subsequent mutant modeling.

The change in binding energy (*∆∆G*) caused by EGFR point mutation was used as a criterion for method suitability testing. One hundred structural models were generated for each of the EGFR^S492R^/cetuximab and EGFR^G465R^ /cetuximab complexes, and their corresponding *∆∆G* values (see Eq. 1) were simultaneously calculated via a RosettaScripts protocol using the ref2015 energy function. In addition, the InterfaceAnalyzer application was used to determine the difference in binding energy (dG_separated) between the EGFR mutants and wild-type EGFR.

1 $$\Delta \Delta G=\Delta {G}_{{{{{{{\rm{EGFR}}}}}}}^{{{{{{\rm{mutant}}}}}}/{{{{{\rm{cetuximab}}}}}}}}{{{{{\rm{-}}}}}}\Delta {G}_{{{{{{{\rm{EGFR}}}}}}}^{{{{{{\rm{wild}}}}}}-{{{{{\rm{type}}}}}}/{{{{{\rm{cetuximab}}}}}}}}$$

All predicted structures of EGFR^S492R^/cetuximab and EGFR^G465R^/cetuximab were clustered according to the interface residues between the antigen and antibody. Three representative structures were selected for each EGFR^S492R^/cetuximab and EGFR^G465R^/cetuximab complex. For cetuximab’s amino acids within 5 Å of any residue of EGFR, a systematic single point mutation scan was performed by using Rosetta’s pmut_scan application. The binding energies of all EGFR/cetuximab^mutant^ and EGFR/cetuximab^wild-type^ complexes were calculated by using the InterfaceAnalyzer application. Finally, cetuximab variants potentially effective against EGFR mutants were identified based on the change in binding energy (see Eq. 2).

2 $$\Delta \Delta G=\Delta {G}_{{{{{{{\rm{EGFR}}}}}}/{{{{{\rm{cetuximab}}}}}}}^{{{{{{\rm{mutant}}}}}}}}{{{{{\rm{-}}}}}}\Delta {G}_{{{{{{{\rm{EGFR}}}}}}/{{{{{\rm{cetuximab}}}}}}}^{{{{{{\rm{wild}}}}}}-{{{{{\rm{type}}}}}}}}$$

### Generation of stable cell lines

To generate cell lines with stable expression of EGFR and its mutants, the sequence encoding N-terminal Flag-tagged full-length human wild-type *EGFR* was inserted into the expression vector pCMV3-hygromycin/ampicillin (Sino Biological, China). S492R-, I491M-, K489E-, K467T-, G465R-, G465E-, S464L-, I462R-, R451C-, S442R- and V441D-mutated *EGFR* constructs were generated by site-directed mutagenesis using the wild-type *EGFR* construct as a template. Primers used in this experiment were listed in Supplementary Table 9. NIH3T3 cells were transfected with the wild-type or *EGFR* mutant constructs by a Lipofectamine 3000 transfection kit according to the manufacturer’s protocol (Invitrogen, Carlsbad, CA, USA) and were then treated with hygromycin B (300 μg/mL; Solarbio, China) for 96 h. After immunostaining with a mouse polyclonal anti-Flag tag antibody (Genscript, China) at a dilution of 1:500 and a FITC-conjugated goat anti-mouse IgG (H + L) secondary antibody at a dilution of 1:500, stable cells expressing wild-type or mutant EGFR were sorted by fluorescence-activated cell sorting (FACS) in a MoFlo Astrios EQ flow cytometer (Beckman, USA). Single NIH3T3 cell clones that stably expressed wild-type or mutant EGFR (S492R, I491M, K489E, K467T, G465R, G465E, S464L, I462R, R451C, S442R, or V441D) were identified with an ACEA NovoCyteTM flow cytometer (ACEA Biosciences, USA). Stable HEK293T cells harboring C-terminal eGFP-tagged human wild-type, S492R-mutated or G465R-mutated EGFR were generated as described above. Stable SW48 cells overexpressing G465R-mutated EGFR and stable COLO320DM cells overexpressing wild-type, S492R-mutated or G465R-mutated EGFR were also generated as described above.

### Recombinant EGFR-ECD-Fc fusion protein

The fusion gene fragment of the human wild-type EGFR ectodomain (ECD, residues 25–644) and the human IgG1 Fc-fragment were cloned into the expression vector pMH3. The S492R-EGFR-ECD-Fc, G465E-EGFR-ECD-Fc and G465R-EGFR-ECD-Fc mutant constructs were generated by site-directed mutagenesis. Primers used in this experiment were listed in Supplementary Table 9. The recombinant EGFR-ECD-Fc fusion protein and mutants were expressed in HEK293F cells through transient transfection with polyethylenimine (PEI). Recombinant fusion proteins were purified from culture supernatants using a HiTrap Protein A column (GE Healthcare, PA, USA) in an ÄKTA pure system (GE Healthcare, PA, USA), dialyzed against PBS (pH 7.4) and stored in −80 °C for further study.

### Generation of phage-display libraries

The single-chain variable fragment (scFv) of cetuximab was cloned into the SfiI-NotI restriction sites of the phagemid vector pCANTAB-5E. On the basis of computational prediction, the S492R-associated library was constructed with random mutations in CDR2 and CDR3 of the VH domain (targeting Val50, Asp58; Tyr104), and CDR1 and CDR3 of the VL domain (targeting Asn32; Trp94, Thr96). The G465R-associated library was generated with random mutations in CDR2 and CDR3 of the VH domain (targeting Val50, Trp52). These residues were mutated to 20 other amino acids by introducing NNS (N = A, T, C or G; S = C or G) codons in the oligonucleotide primer, and the corresponding scFv gene fragments were amplified by PCR. Primers used in this experiment were listed in Supplementary Table 9. The resulting mutant scFv gene libraries were transfected into *Escherichia coli* XL1 BLUE to generate construct bacterial libraries. Each library was pooled and grown overnight at 30 °C in 2XTY broth supplemented with M13KO7 helper phage, 100 μg/mL ampicillin, 50 μg/mL kanamycin and 50 μg/mL tetracycline. Phages were precipitated with 20% PEG-8000/2.5 M NaCl followed by centrifugation at 5,000×g. The precipitated phages were washed with cold PBS before suspension in PBS (pH 7.4)-buffered 2% (w/v) nonfat milk (blocking buffer). To select phages that display cetuximab-derived scFvs capable of binding to EGFR^S492R^ or EGFR^G465R^, recombinant phages were first prepanned with parental NIH3T3 cells in blocking buffer to remove unbound phages, and subsequently panned with S492R-EGFR-NIH3T3 cells or G465R-EGFR-NIH3T3 cells after blocking. After 2 h of incubation, the cells were washed ten times with PBS. Bound phages were then eluted by incubation with glycine elution buffer (pH 2.2) for 15 min and neutralized by the addition of 2 M Tris-HCl buffer (pH 8.0). Phages were further screened in WT-EGFR-NIH3T3 cells to obtain WT and mutant EGFR dual binders. The eluted phages were amplified by reinfection of *E. coli* XL1 BLUE, followed by two additional rounds of selection. Eluted phages from the third round of panning were used to reinfect *E. coli* XL1 BLUE, and single clones were then expanded in 96-well microtiter plates and induced to express soluble scFv antibody with 0.1 mM isopropyl β-D-1-thiogalactopyranoside (IPTG). Culture supernatants containing the C-terminal E tag-fused scFv antibody were analyzed by enzyme-linked immunosorbent assays (ELISAs). ELISA plates were precoated with recombinant S492R-EGFR-ECD-Fc or G465R-EGFR-ECD-Fc, blocked with 5% (w/v) milk/PBS (pH 7.4), and then washed with PBST (pH 7.4, containing 1% Tween 20). Bound scFv antibodies were detected using an HRP-conjugated anti-E tag antibody (Abcam, Cambridge, MA, USA) at a dilution of 1:1000 in 5% (w/v) milk/PBS (pH 7.4), followed by incubation with 3′,3′,5,5′-tetramethylbenzidine (TMB) substrate. Positive phage clones were sequenced to obtain various scFv sequences.

### Recombinant antibodies

VH and VL domain fragments from selected anti-EGFR scFvs were subcloned into the expression vector pMH3 to generate the full-length human IgG1 (Cκ) format. The cetuximab variants Ctx-Y104X constructs were generated by site-directed mutagenesis. Primers used in this experiment were listed in Supplementary Table 9. For the control antibody, the VH and VL domains of another anti-EGFR antibody, panitumumab, were cloned into the expression vector pMH3 to generate full-length human IgG2/κ. Recombinant antibodies were produced in HEK293F cells through transient transfection. Antibodies were purified from culture supernatants using a HiTrap Protein A column (GE Healthcare, PA, USA) in an ÄKTA pure system (GE Healthcare, PA, USA) and were dialyzed against PBS (pH 7.4). The purity and homogeneity of cetuximab variants were analyzed by size-exclusion chromatography-high performance liquid chromatography (SEC-HPLC) and date was acquired with the Agilent 1200 & EZChrom Elite software (Agilent Technologies, Palo Alto, CA, USA). The stability of cetuximab variants were analyzed by dynamic light scattering (DLS). LitesizerTM 500 (Anton paar, USA) was used for DLS data acquisition.

### Antibody binding ability in wild-type or mutant EGFR-expressing stable cell lines

To determine the binding affinity of therapeutic antibodies in cell lines, wild-type or mutant EGFR (i.e., S492R, I491M, K489E, K467T, G465R, G465E, S464L, I462R, R451C, S442R, or V441D)-expressing NIH3T3 cells were harvested by trypsinization and washed twice with cold PBS (pH 7.4). Each cell line was stained with cetuximab or panitumumab (1, 10, and 100 nmol/L, 30 min) on ice, washed twice with ice-cold PBS (pH 7.4) and incubated with a FITC-conjugated goat anti-human IgG (H + L) secondary antibody (Beyotime, China) at a 1:500 dilution in PBS (pH 7.4) for 30 min on ice. Samples were washed and resuspended in ice-cold PBS (pH 7.4) before analysis via an ACEA NovoCyteTM flow cytometer (ACEA Biosciences, USA). The percentages of monoclonal antibody-stained cells among total EGFR-positive cells were determined. The mean fluorescence intensity (MFI) of the immunostained cells was also determined by flow cytometry. FACS sequential gating strategy is shown in Supplementary Fig. 9a. To evaluate the binding ability of the cetuximab variants, wild-type EGFR, EGFR^S492R^, EGFR^G465E^ or EGFR^G465R^-expressing NIH3T3 cells were treated with serial concentrations of cetuximab, panitumumab or the cetuximab variants, and were then fluorescently labeled with the FITC-conjugated goat anti-human IgG (H + L) secondary antibody (Beyotime, China) at a dilution of 1:500 before flow cytometric analysis. FACS sequential gating strategy is shown in Supplementary Fig. 9b. Data were analyzed by GraphPad Prism software.

### Antibody binding ability with EGFR-ECD

The binding of cetuximab, panitumumab, and the cetuximab variants to EGFR-ECD was analyzed by ELISA. Ninety-six-well microtiter plates were coated with WT-EGFR-ECD-Fc, S492R-EGFR-ECD-Fc, G465E-EGFR-ECD-Fc, and G465R-EGFR-ECD-Fc at a concentration of 1 µg/mL overnight at 4 °C. Antibodies were diluted in PBS with 5% (w/v) nonfat milk and added to the coated plates at concentrations ranging from 1 × 10^−4^ nM to 100 nM. The plate wells were washed five times with PBST. Antibodies were detected with an HRP-conjugated polyclonal goat anti-human kappa light chain antibody (Thermo Fisher Scientific, USA) at a dilution of 1:500 using TMB as the substrate.

### Surface plasmon resonance

Antibody binding affinities were determined by a NeoSPR-M100 instrument (Neoline, Hangzhou, China). Flow cells composed of carboxyl chips (Neoline, Hangzhou, China) were coupled with recombinant WT-EGFR-ECD-Fc, S492R-EGFR-ECD-Fc and G465R-EGFR-ECD-Fc through amine-coupling chemistry, and the coupling buffer PBS was used as a reference. Cetuximab or the cetuximab variants were injected over the immobilized WT-EGFR-ECD-Fc, S492R-EGFR-ECD-Fc or G465R-EGFR-ECD-Fc (coupled at densities ranging from 96-293 relative units to flow cells composed of carboxyl chips) at a flow rate of 20 µL/min at 25 °C in PBS. Flow cells were regenerated following each injection using 30 mM NaOH buffer. Kinetic parameters were analyzed by TraceDrawer software.

### Inhibition of EGFR signaling

WT-EGFR-NIH3T3, S492R-EGFR-NIH3T3 and G465R-EGFR-NIH3T3 cells were plated in 6-well plates and serum starved overnight in a 37 °C incubator with 5% CO_2_. Then, the cells were treated with 100 nM cetuximab, panitumumab, Ctx-VY, Ctx-Y104D, Ctx-W52D or control IgG for 2 h and further stimulated for 15 min with 10 ng/mL EGF. Lysates were subjected to western blot analysis for detection of EGFR downstream signaling by various primary antibodies: rabbit anti-EGFR and rabbit anti-pEGFR (Tyr1068) antibodies (Cell Signaling Technology, Danvers, USA), rabbit anti-Akt and rabbit anti-pAkt (Ser473) antibodies (Cell Signaling Technology, Danvers, USA), and rabbit anti-Erk and rabbit anti-pErk (Thr202/Tyr204) antibodies (Cell Signaling Technology, Danvers, USA). HRP-conjugated goat anti-rabbit IgG (H + L) (Beyotime, China) at a dilution of 1:2000 was used as the secondary antibody. Immunoreaction signals were visualized by a ChemiDoc Touch Imaging System (Bio-Rad, USA) with ECL reagents.

### Induction of EGFR degradation

Antibody-induced EGFR-eGFP degradation was assessed by flow cytometry. HEK293T cells overexpressing WT-EGFR-eGFP, S492R-EGFR-eGFP and G465R-EGFR-eGFP were seeded in 48-well plates overnight in a 37 °C incubator with 5% CO_2_, and were then treated with cetuximab, panitumumab, Ctx-VY, Ctx-Y104D, Ctx-W52D or control IgG for 48 hours. Mean fluorescence intensity (MFI) values were normalized to those in the untreated controls. FACS sequential gating strategy is shown in Supplementary Fig. 9c. Data were analyzed by GraphPad Prism software.

### Inhibition of cell proliferation

SW48, G465R-EGFR-SW48 and COLO320DM cells stably overexpressing wild-type or mutant EGFR were seeded in 96-well plates overnight in a 37 °C incubator with 5% CO_2_ and treated with serial concentrations of therapeutic antibodies or cetuximab variants. Three days post treatment, cell viability was evaluated using a Cell Counting Kit-8 (CCK-8, Japan) and measuring the absorbance at 450 nm in a Model 680 microplate reader (Bio-Rad, USA). The cell proliferation inhibition rate is shown relative to cells treated with PBS only. Data were analyzed in GraphPad Prism software.

### ADCC and CDC assays

For the antibody-dependent cellular cytotoxicity (ADCC) assay, ten thousand target cells were coincubated with 5×10^5^ human peripheral blood mononuclear cells (PBMCs) for 10 hours, and cell viability was determined by a lactate dehydrogenase (LDH) detection kit (LDH Cytotoxicity Assay Kit; Beyotime). The percentage of specific lysis was determined as follows: % lysis = (experimental LDH release – spontaneous LDH release)/(maximum LDH release − spontaneous LDH release) × 100. All assays were performed in triplicates. The results are presented as the mean ± SD values. For the complement-dependent cytotoxicity (CDC) assays, antibodies were incubated with 1 × 10^4^ target cells/well and 10% human serum for 2 hours. Cell viability was determined by a Cell Counting Kit-8 (CCK-8, Japan). The percentage of specific lysis was determined as follows: % lysis = (experimental lysis - nonspecific lysis) × 100. Experiments were performed in triplicate. The results are presented as the mean ± SD values.

### In vivo efficacy of antibodies in mouse xenograft models

Mouse xenograft models bearing EGFR^WT^- or EGFR^Mut^- positive cells were generated by subcutaneous inoculation of 5 × 10^6^ SW48 (WT-EGFR), G465R-EGFR-SW48 (WT/G465R-EGFR-Heterozygote), WT-EGFR-COLO320DM, S492R-EGFR-COLO320DM or G465R-EGFR-COLO320DM cells into the right flanks of nude mice. When the mean tumor volume reached ~50 mm^3^, the mice were randomly divided into 20 groups (PBS, cetuximab, Ctx-VY, Ctx-Y104D and Ctx-W52D; *n* = 5 mice per group). Cetuximab or the cetuximab variants were injected intravenously into xenografted mice via the tail vein at a dosage of 25 mg/kg, once every 3 days for four times (q3d×4). Mouse body weights were measured once every 2–3 days with an electronic balance, and the tumor size was measured every 2–3 days with an electronic caliper; the tumor volume was calculated using the formula (L × W × W)/2, in which *L* represents the larger diameter of the tumor and *W* represents the smaller diameter. Tumor tissues were collected for preparation of paraffin sections for the terminal deoxynucleotidyl transferase-mediated dUTP nick end labeling (TUNEL) staining assay, immunohistochemical staining (EGFR and Ki-67), and hematoxylin and eosin (H&E) staining. Organs, including the heart, liver, spleen, lung, and kidney, were isolated for preparation of paraffin sections for H&E staining. Serum was collected for measurement of ALT, AST, BUN, and Cr to evaluate liver and kidney function. A Cobas c311 analyzer (Roche Diagnostics GmbH, Germany) was used to test ALT, AST, BUN and Cr.

### Immunohistochemical staining

Tumor tissues were fixed with 10% formaldehyde and embedded in paraffin. After dewaxing, rehydration, and antigen retrieval, sections were blocked with goat serum blocking solution and were then stained using rabbit polyclonal anti-EGFR (1:200, Beyotime, AF5153) or rabbit polyclonal anti-Ki-67 (1:500, Abcam, ab15580) antibodies overnight at 4 °C. After staining with the HRP-conjugated goat anti-rabbit IgG (H + L) antibody (Zhongshan Goldbridge Biotechnology, PV6001, 100 μL per sample) at 37 °C for 30 min, tumor tissue sections were visualized by a DAB detection kit (Boster Biological Technology, AR1026) and an Olympus BX61 light microscope (Olympus, Shinjuku-ku, Tokyo, Japan). For detection of apoptosis, paraffin-embedded sections of tumor tissues were labeled by a POD-TUNEL Kit (Roche Inc.) according to the manufacturer’s instruction. All images were acquired by an Olympus BX61 light microscope. Quantification was performed at ×400 magnification (3 images per tumor) using ImageJ 1.52a software.

### Statistics and reproducibility

The statistical analysis is described in the “Methods” section or figure legends. All the experiments were performed at least in triplicate and repeated.

### Reporting summary

Further information on research design is available in the Nature Research Reporting Summary linked to this article.

## Supplementary information

Supplementary Information

Reporting Summary

Description of Additional Supplementary Files

Supplementary Data 1

Supplementary Data 2

Supplementary Data 3

Supplementary Data 4

Supplementary Data 5

Supplementary Data 6

## Data Availability

The structures of wild-type EGFR ECD/cetuximab Fab complex was obtained from the Protein Data Bank (PDB code: 1YY9). All data generated or analyzed during this study are included in this article, its Supplementary Information files and the Source Data file. The uncropped gel or blot figures and original data underlying Figs. 1–7 and Supplementary Figs. 1–9 are provided as a Source Data file. Source data are provided with this paper.

## Source data

Source Data
