# Supplementary material for: Structure-guided and phage-assisted evolution of a therapeutic anti-EGFR antibody to reverse acquired resistance
Source: Nat Commun. 2022 Jul 30;13:4431. doi: 10.1038/s41467-022-32159-6 (PMC9338999; doi:10.1038/s41467-022-32159-6)

Supplementary Information

Structure-guided and phage-assisted evolution of a therapeutic anti-EGFR antibody to reverse acquired resistance

Xinlei Zhuang[1], Zhe Wang[1], Jiansheng Fan[1], Xuefei Bai[1], Yingchun Xu[1], James J. Chou[2], Tingjun Hou[1], Shuqing Chen[1,3]* and Liqiang Pan[1,4]*

1. Institute of Drug Metabolism and Pharmaceutical Analysis, College of Pharmaceutical Sciences, Zhejiang University, Hangzhou 310058, China.

2. Department of Biological Chemistry and Molecular Pharmacology, Harvard Medical School, 250 Longwood Avenue, Boston, MA 02115.

3. Department of Precision Medicine on Tumor Therapeutics, ZJU-Hangzhou Global Scientific and Technological Innovation Center, Hangzhou 311200, China.

4. The First Affiliated Hospital, Zhejiang University School of Medicine, Hangzhou 310003, China; Key Laboratory of Pancreatic Disease of Zhejiang Province, Hangzhou 310003, China.

*Correspondence:

Shuqing Chen (chenshuqing@zju.edu.cn) or Liqiang Pan (panliqiang@zju.edu.cn)

Supplementary Figures

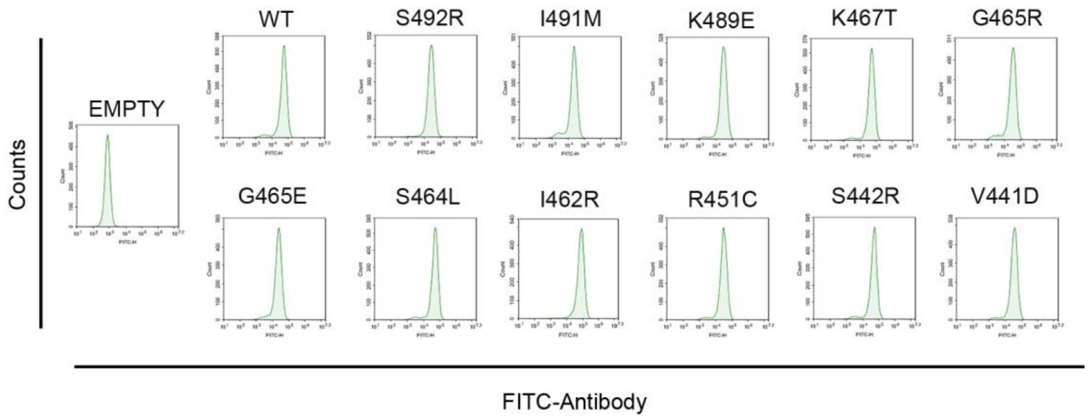

Supplementary Fig. 1. Identification of EGFR^{WT} or EGFR^{Mut} NIH3T3 stable cells, Related to Fig. 1.

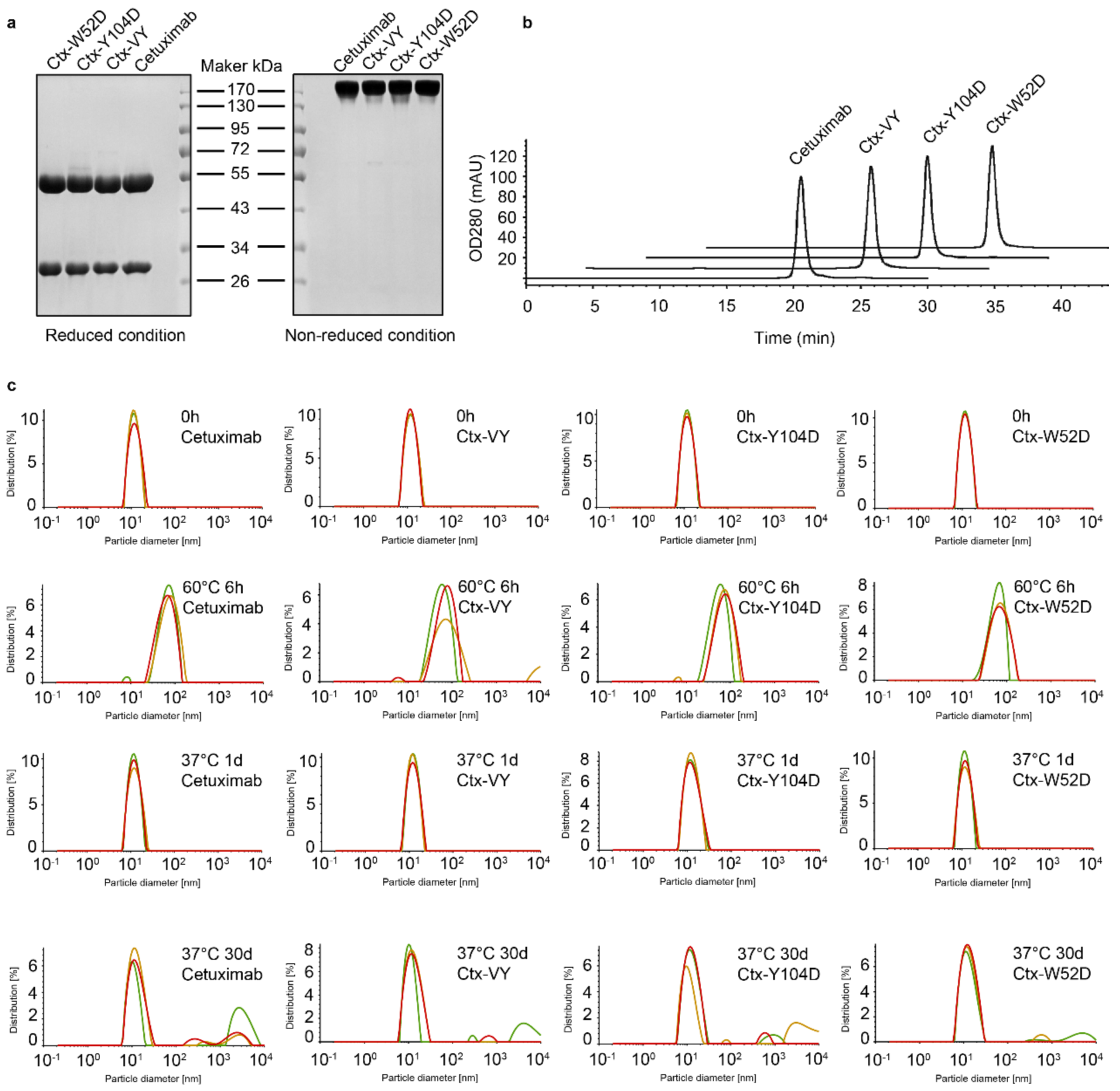

Supplementary Fig. 2. The stability and oligomerization state of the cetuximab and cetuximab variants, Related to Fig. 4.

(a) The reducing and nonreducing SDS–PAGE results for purified cetuximab and its variants. **(b)** The size-exclusion chromatography results for cetuximab and its variants. **(c)** Measurement of aggregation formation in cetuximab and cetuximab variants after incubation at 60°C and 37°C by DLS. Source data are provided as a Source Data file.

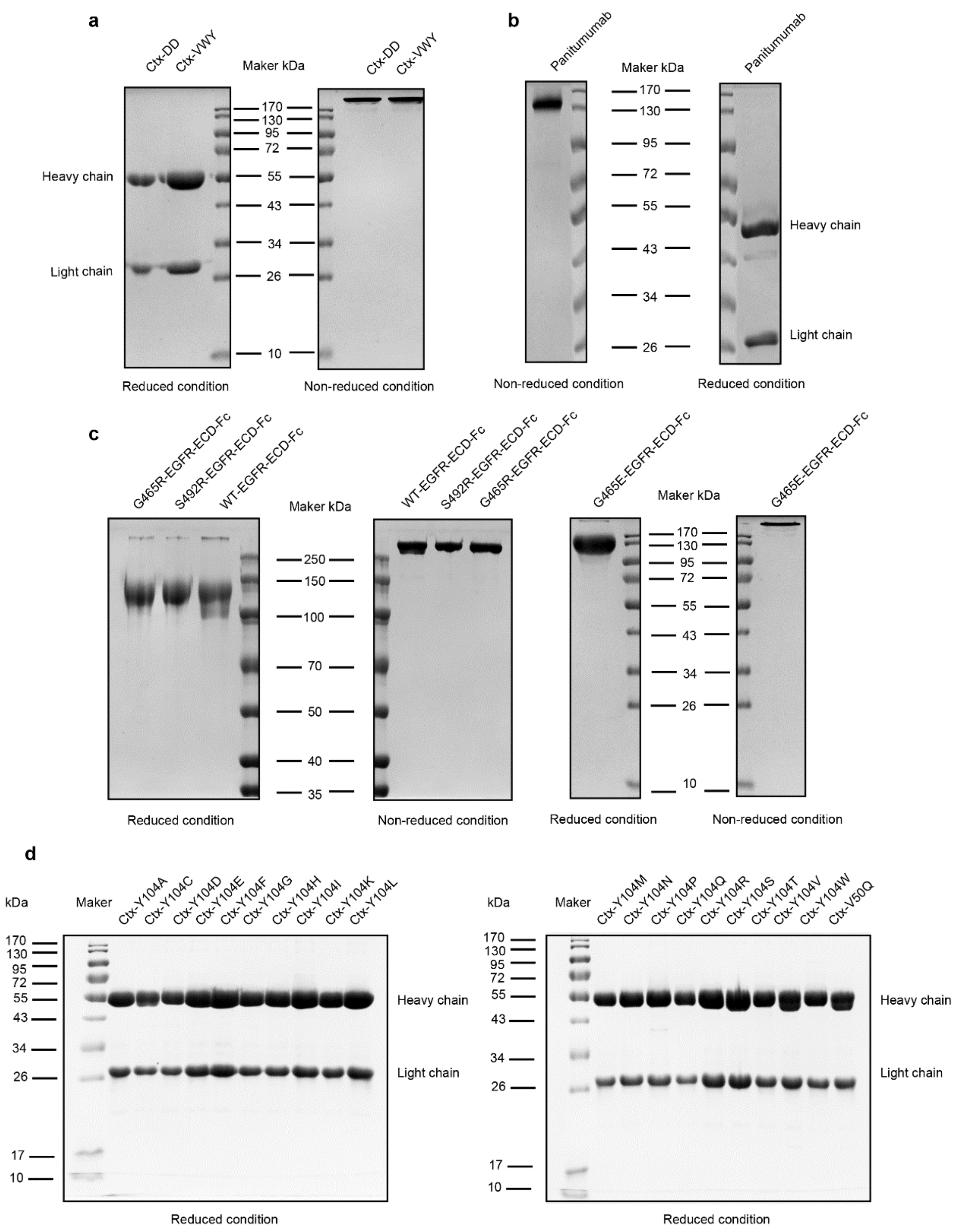

Supplementary Fig. 3. Characterization of the purified anti-EGFR antibodies, and EGFR-ECD-Fc protein, Related to Fig. 4.

(a) The reducing and non-reducing SDS-PAGE results of the purified panitumumab under reduced and non-reduced condition. **(b)** The reducing and non-reducing SDS-PAGE results of the purified Ctx-DD and Ctx-VWY. **(c)** The reducing and non-reducing SDS-PAGE results of the purified WT-EGFR-ECD-Fc, S492R-EGFR-ECD-Fc,

G465R-EGFR-ECD-Fc and G465E-EGFR-ECD-Fc protein. **(d)** The reducing SDS-PAGE result of the purified Ctx-Y104X and Ctx-V50Q. Source data are provided as a Source Data file.

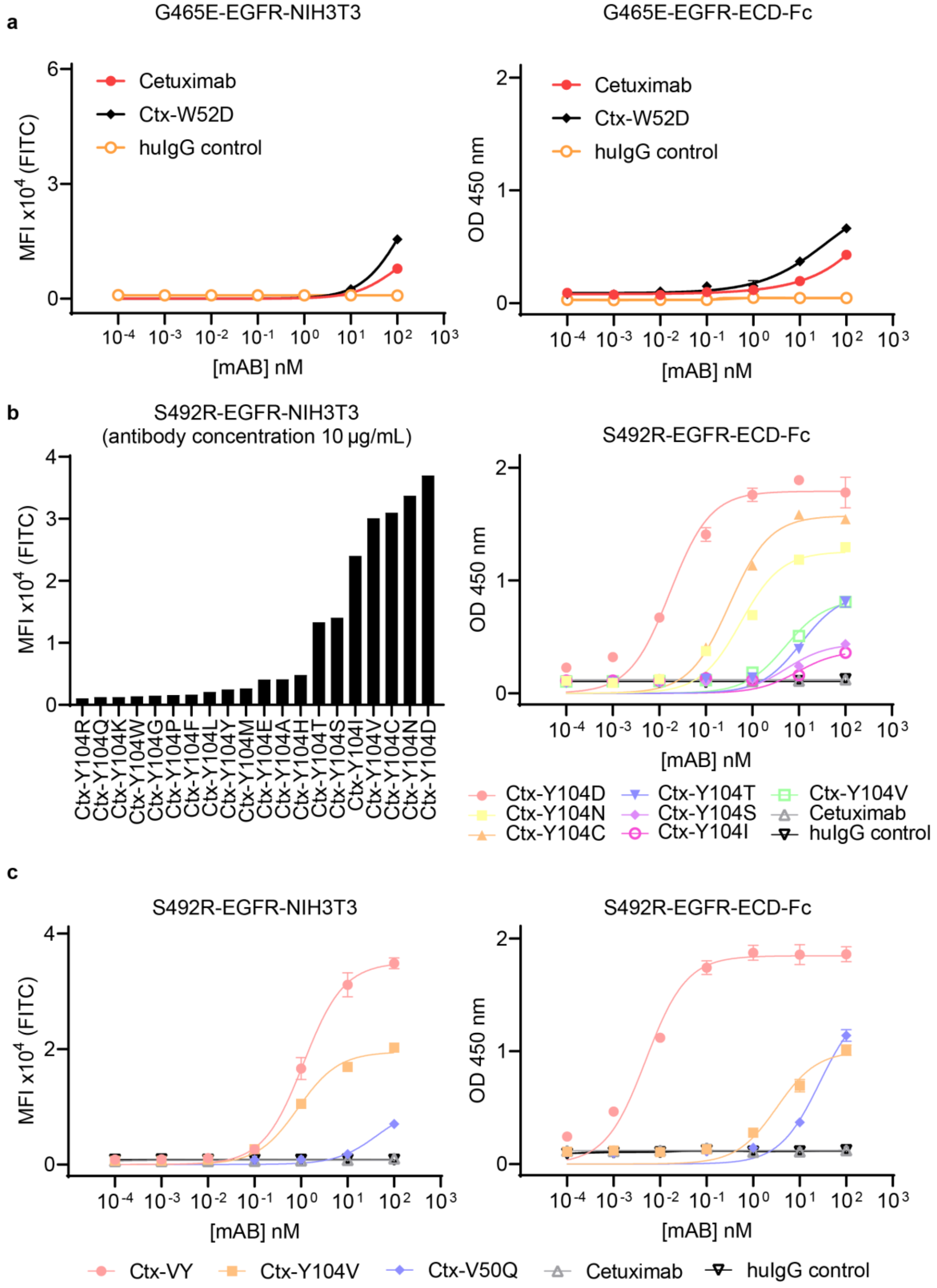

Supplementary Fig. 4. The binding ability of cetuximab variants with mutated EGFR, Related to Fig. 4.

(a) The binding ability of cetuximab and Ctx-W52D with (left panel) NIH3T3 cells expressing $EGFR^{G465E}$ or with (right panel) G465E-EGFR-ECD-Fc fusion protein. All

data are shown in mean ± SD values, n=3. **(b)** Site saturation mutagenesis of the Y104 residue of cetuximab and its effect on reversing $EGFR^{S492R}$-mediated resistance. The binding affinity of Ctx-Y104X for S492R-EGFR-NIH3T3 cells was evaluated by flow cytometry (left panel). The binding affinity of the top 7 high-affinity cetuximab variants for S492R-EGFR-ECD-Fc protein was further analyzed by ELISA (right panel). All data are shown in mean ± SD values, n=3. **(c)** The effect of two substitutions on the binding affinity of Ctx-VY for $EGFR^{S492R}$. The binding ability with S492R-EGFR-NIH3T3 cells was determined by flow cytometry (left panel) and with the S492R-EGFR-ECD-Fc protein by ELISA (right panel). All data are shown in mean ± SD values, n=3. Source data are provided as a Source Data file.

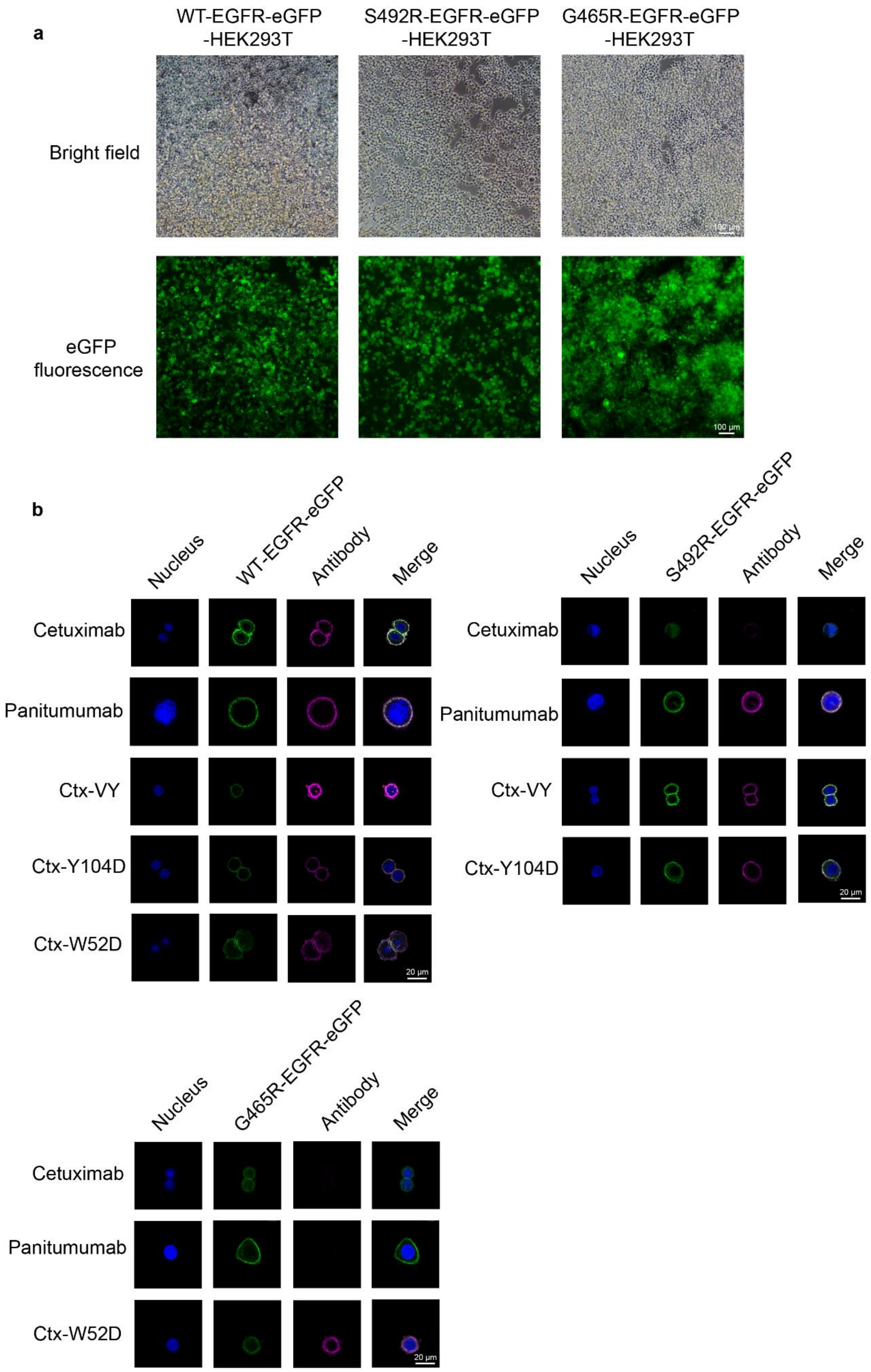

Supplementary Fig. 5. Confocal imaging of HEK293T cells expressing full-length WT-EGFR-eGFP, S492R-EGFR-eGFP and G465R-EGFR-eGFP after

immunostaining by cetuximab variants, Related to Fig. 5.

(a) Identification of the EGFR-eGFP expression in HEK293T stable cell Lines. Scale bars: 100 μm. **(b)** The cell-surface expression of WT-/Mut- EGFR in HEK293T stable cell lines. Scale bars: 20 μm.

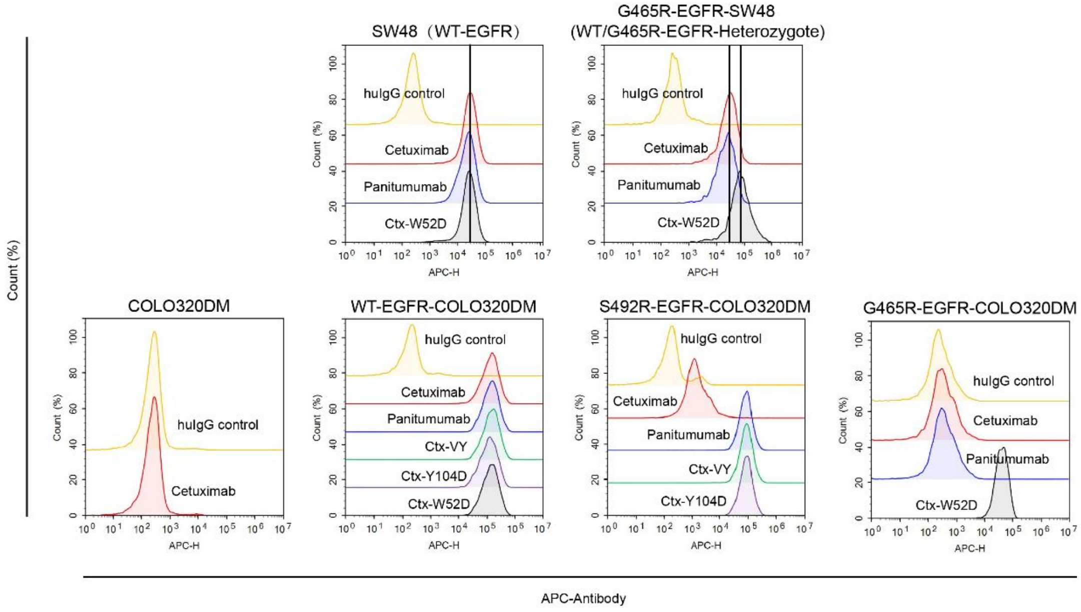

Supplementary Fig. 6. The cell-surface expression of WT-/Mut- EGFR in SW48 (WT-EGFR), COLO320DM, WT-EGFR-COLO320DM, S492R-EGFR-COLO320DM and G465R-EGFR-COLO320DM cells, Related to Figure 6 and Fig. 7.

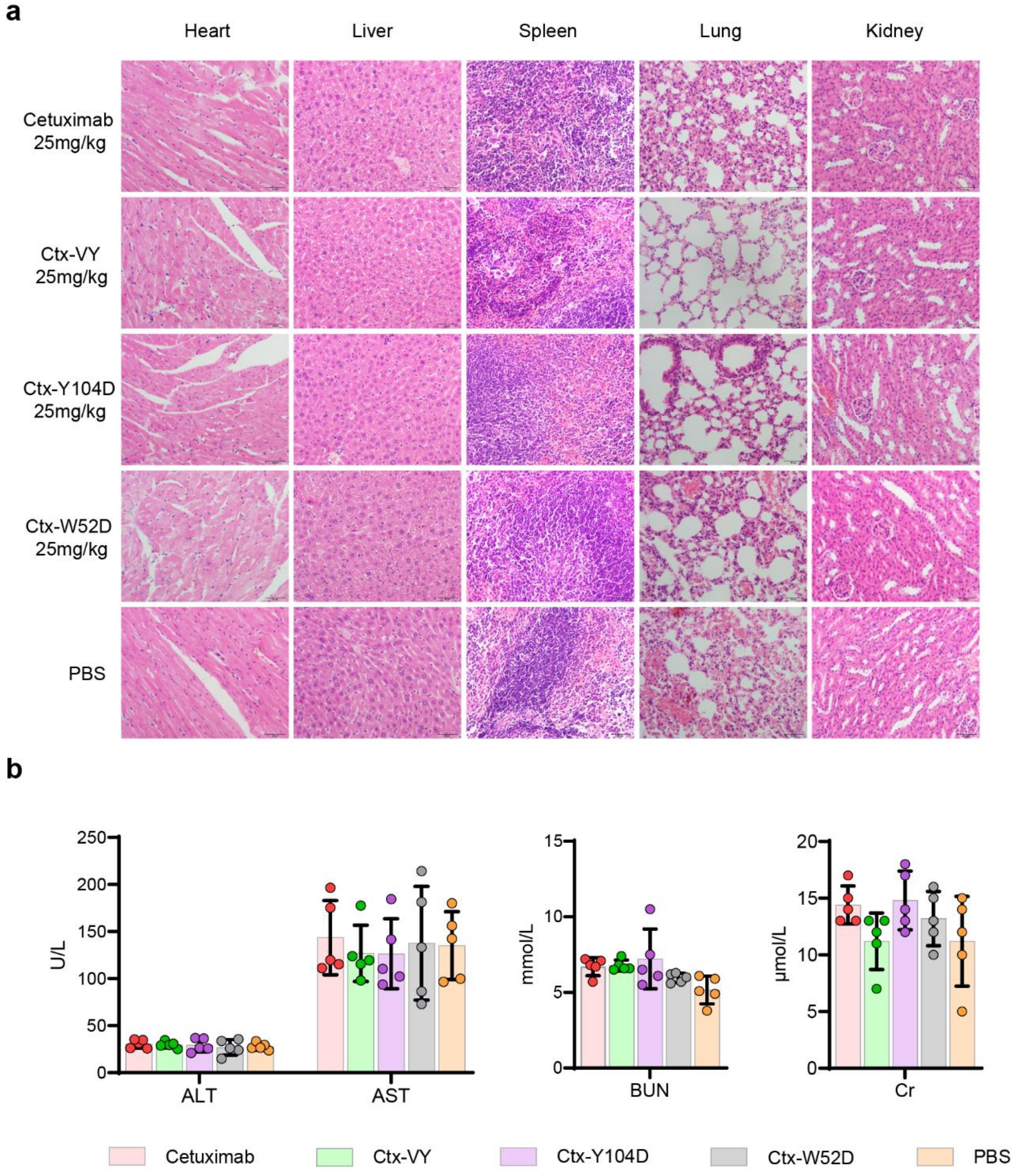

Supplementary Fig. 7. The toxicity profile of cetuximab variants, Related to Fig. 7.

(a) The H&E staining of different tissue sections from heart, liver, spleen, lung, and kidney after antibody treatment. Scale bars: 50 µm. **(b)** Serum levels of ALT, AST, BUN, and Cr after antibody treatment. All data are shown in mean ± SD values, n=5 mice for each group. Source data are provided as a Source Data file.

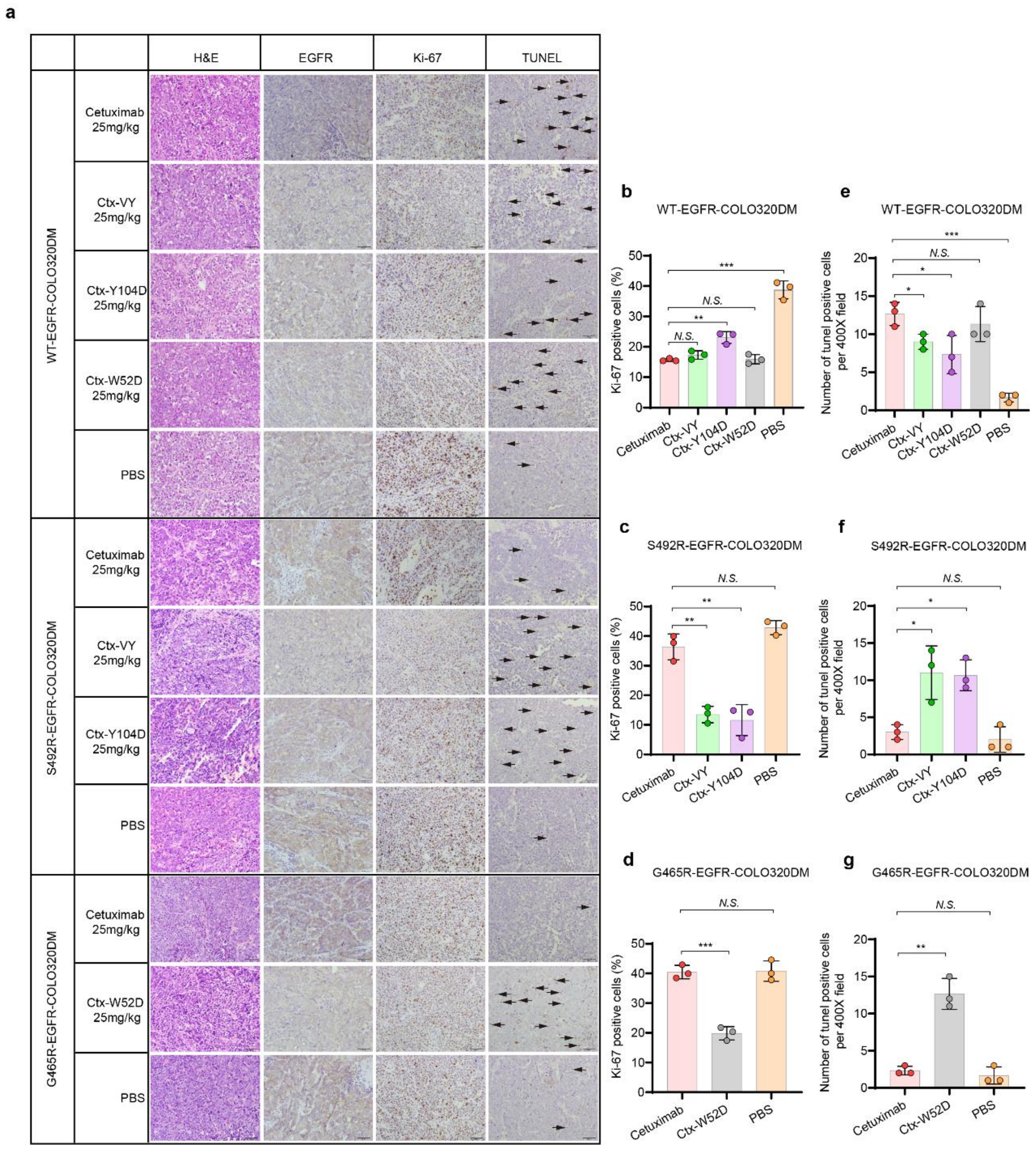

Supplementary Fig. 8. Histological examinations of tumor tissues after administration of cetuximab and cetuximab variants, Related to Fig. 7.

(a) Histological examinations of tumor tissues after H&E staining, after EGFR and Ki-67 staining, and after the TUNEL assay. Scale bar, 50 μm. **(b, c and d)** The percentages of Ki-67-positive cells from (a). **(e, f and g)** Quantification of TUNEL-positive cells from (a). The number of positive cells was counted in three randomized microscopic fields of view. All data (b-g) are shown in mean ± SD values, n=3 mice for each group (*p < 0.05, **p < 0.01, ***p < 0.001, ****p< 0.0001, *N.S.* means not significant; two-tailed Student's t-test.). Source data are provided as a Source Data file.

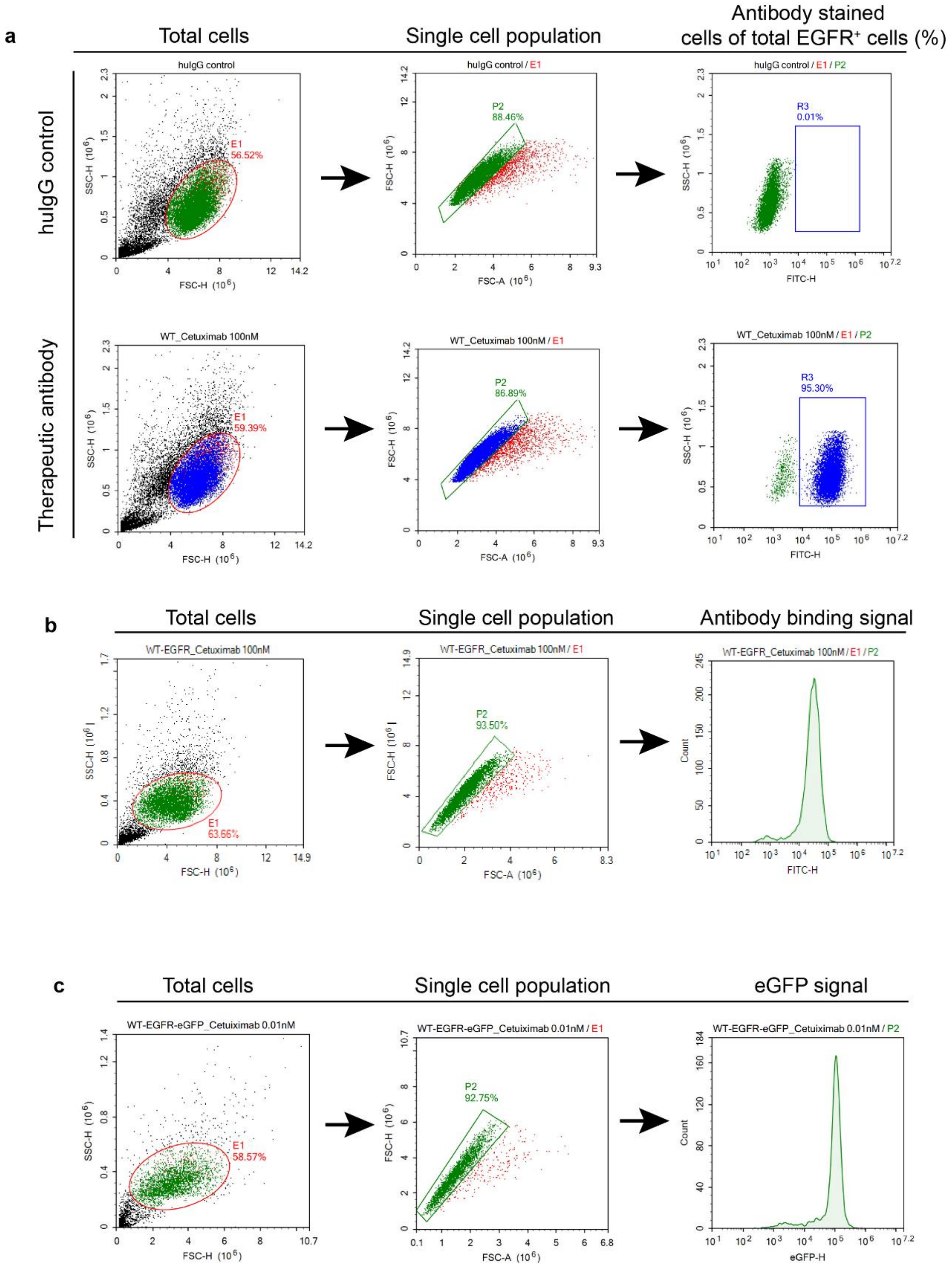

Supplementary Fig. 9. FACS sequential gating strategies.

(a) FACS sequential gating strategy related to Fig. 1b. **(b)** FACS sequential gating strategy related to Fig. 4c. **(c)** FACS sequential gating strategy related to Fig. 5d.

Supplementary Tables

Supplementary Table 1. The prediction results of EGFR^{Mut}/ cetuximab interaction by Rosetta platform are consistent with the validation result of the acquired cetuximab-resistant EGFR mutations after cetuximab treatment, Related to Figure 3.

Mutation	Patient tumors	Cell lines	Reported effect on binding	Experimental binding	Calculated *ΔΔ G* (Rosetta energy unit)	
					RosettaScripts	InterfaceAnalyzer
S492R	Resistant<br>（1，2）	Resistant<br>（1，2）	No binding<br>（1）	No binding	132.1	150.2
G465R	Resistant<br>（3）	Resistant<br>（1）	No binding<br>（1,4）	No binding	258.8	282.3

Supplementary Table 2. The ΔΔ*G* values for Cluster 1 of EGFR^{S492R}/Cetuximab^{Mut}, Related to Figure 3.

Residue	A	C	D	E	F	G	H	I	K	L	M	N	P	Q	R	S	T	V	W	Y
VL-D1	-9.422	-9.491	NaN	-9.269	-30.241	-9.635	-9.359	-9.172	-9.372	-9.375	-9.365	-10.956	-9.388	-9.196	-9.357	-9.308	-9.122	-9.175	-9.398	-9.425
VL-S26	-13.598	-13.573	-13.575	-13.628	-13.573	-13.599	-13.567	-13.602	-13.579	-13.611	-13.57	-13.579	-13.796	-13.588	-13.577	NaN	-13.645	-13.594	-13.58	-13.589
VL-Q27	-10.468	-10.164	-7.73	-12.011	-8.468	-10.018	-7.929	-0.271	-6.026	-9.666	-11.181	-8.272	-7.554	NaN	-6.638	-9.937	-10.416	-11.448	-6.049	-8.405
VL-S28	-12.615	-12.777	-12.511	-11.094	-12.342	-12.288	-12.732	-12.443	-12.2	-12.258	-12.185	-13.003	-7.397	-12.687	-12.441	NaN	-33.343	-11.155	-12.055	-12.076
VL-T31	-11.497	-11.497	-11.977	-11.502	-11.494	-11.515	-11.499	-11.399	-11.498	-11.495	-11.498	-11.501	-11.748	-11.5	-11.509	-11.457	NaN	-11.471	-11.499	-11.27
VL-N32	-181.404	-182.122	-179.341	-182.346	-183.697	-180.789	-181.961	-183.285	-179.773	-180.292	-181.257	NaN	-176.834	-181.148	-176.969	-181.149	-181.84	-182.709	-177.533	-183.603
VL-H34	-177.962	-177.953	-177.986	-178.15	-183.973	-177.72	NaN	-177.994	-177.723	-183.924	-183.941	-177.98	-178.487	-178.034	-183.696	-177.962	-177.97	-177.71	-184.703	-184.063
VL-Y50	-13.224	-13.396	-16.476	-13.075	-12.235	-15.2	-11.086	-13.651	-8.914	-12.796	-13.878	-12.934	-13.22	-13.11	-9.213	-13.034	-12.844	-13.439	-11.953	NaN
VL-E53	-8.702	-8.701	-9.923	NaN	-7.583	-8.726	-8.012	-8.493	-7.605	-8.03	-8.143	-7.721	-8.742	-10.411	-7.457	-8.7	-8.722	-8.56	-7.712	-7.537
VL-N91	-186.283	-185.734	-186.058	-209.654	-182.114	-223.29	-184.756	-187.948	-187.023	-183.107	-186.904	NaN	-185.85	-186.09	-176.372	-185.978	-185.177	-186.756	-186.693	-184.1
VL-N92	-42.78	-19.402	-17.514	-15.819	-16.793	-182.795	-19.208	-8.789	-39.601	157.943	-21.761	NaN	-21.68	-17.543	-17.1	-19.815	-18.045	-17.445	-14.306	-17.572
VL-N93	-20.332	-20.956	-20.586	-14.009	-2.785	-20.031	-10.058	-1.65	67.611	4.023	7.444	NaN	-6.124	-11.331	-4.18	-19.555	-14.587	7.277	72.32	7.017
VL-W94	-201.483	-42.609	-36.996	-42.408	-24.822	-27.591	-18.566	0.025	-27.969	-1.009	-3.013	-36.067	85.432	-40.85	-31.752	-206.846	-40.648	-35.676	NaN	-26.54
VL-P95	-23.956	-24.266	-26.932	-22.301	-19.52	-186.141	-21.682	-47.498	17.015	29.354	-23.158	-25.769	NaN	-22.876	-37.46	-24.075	-25.152	-26.513	-22.976	-21.988
VL-T96	-196.444	-221.641	-192.973	-179.586	-194.4	-194.957	-192.218	-186.227	-185.277	-190.822	-190.567	-188.722	-183.141	-171.591	-174.083	-197.437	NaN	-182.967	-25.473	-195.648
VH-N31	-12.283	-12.231	-12.086	-12.238	-12.345	-12.152	-12.189	-11.332	-12.219	-12.457	-12.665	NaN	-12.176	-12.354	-12.899	-12.17	-12.188	-11.157	-12.643	-12.446
VH-H35	-176.35	-176.641	-177.475	-175.253	-178.042	-176.857	NaN	-176.099	-176.052	-177.773	-176.604	-198.098	-176.683	-176.258	-175.024	-176.268	-176.716	-176.086	-183.274	-176.37
VH-W47	-186.837	-226.075	-184.865	-184.797	-228.265	-187.328	-222.306	-218.486	-193.445	-217.928	-184.365	-185.3	-184.743	-186.858	-185.833	-186.714	-187.153	-218.071	NaN	-227.27
VH-G49	-19.471	-19.212	-19.231	-19.656	-19.958	NaN	-19.157	-19.25	-19.393	-19.471	-19.615	-19.216	-18.964	-19.378	-19.964	-19.292	-19.252	-19.48	-20.044	-19.929
VH-V50	-180.546	-180.367	-183.381	-184.205	-184.539	-180.707	-184.056	-182.555	-182.65	-181.566	-185.639	-180.617	-212.727	-183.852	-181.319	-184.002	-179.517	NaN	-182.813	-184.029
VH-W52	-9.722	-10.277	-9.759	-29.555	-10.226	-9.668	-11.336	-10.858	-8.072	-9.211	-12.421	-31.775	-10.294	-11.61	-9.822	-9.747	-10.257	-10.898	NaN	-9.516
VH-S53	-13.563	-12.815	-13.98	-13.762	2.769	-13.038	10.299	-11.222	-0.307	-9.889	-21.403	-11.133	-14.056	-12.57	-12.382	NaN	-13.423	-13.242	13.649	-8.309
VH-G54	-10.683	-3.77	3.342	12.356	27.597	NaN	42.346	98.555	138.431	603.942	30.314	13.427	-1.71	7.983	88.31	-9.567	47.108	82.412	138.744	21.92
VH-G55	-12.644	-12.397	-13.354	-12.557	-13.958	NaN	-12.594	-33.341	-12.141	-33.829	-12.326	-12.157	74.082	-13.071	-12.238	-12.145	-12.387	-12.719	-13.066	-13.862
VH-N56	-11.537	-7.093	-9.139	-4.705	38.196	-10.661	-4.829	-9.606	-2.681	14.118	-9.594	NaN	-11.333	-6.324	-2.746	-10.978	-5.356	-6.648	-10.362	-9.839
VH-T57	-12.094	-12.069	-11.513	-12.092	-11.986	-32.807	-12.108	-12.169	-10.939	-11.997	-12.11	-11.791	-10.974	-11.862	-12.018	-12.053	NaN	-12.06	-12.291	-12.015
VH-D58	-24.144	-23.358	NaN	-21.461	-76.383	-25.759	-15.781	-3.549	-19.747	-25.238	-22.134	-23.285	-12.82	-21.449	-19.617	-24.337	-18.515	-14.409	-165.829	-48.279
VH-Y59	-18.757	-18.614	-18.712	-18.626	-18.681	-18.818	-18.524	-18.554	-18.634	-18.494	-18.623	-18.662	-20.886	-18.936	-18.471	-18.605	-18.515	-18.54	-18.641	NaN
VH-N60	-17.343	-18.69	-20.927	-20.018	-19.04	-23.535	-24.594	-21.059	-20.341	-21.613	-19.354	NaN	-20.324	-20.07	-19.877	-18.438	-16.327	48.815	-18.529	-18.624
VH-T61	-19.316	-19.461	-15.134	-19.288	-19.278	-19.052	-19.342	-15.9	-19.094	-19.683	-19.503	-19.341	-21.739	-19.335	-19.588	-19.181	NaN	-19.46	-19.379	-19.307
VH-N73	-11.998	-11.997	-12.009	-12.098	-11.998	-12.008	-32.671	-12.004	-11.785	-11.994	-12.015	NaN	-11.999	-12.073	-11.968	-11.998	-11.998	-11.998	-12.008	-11.998
VH-T100	-176.072	-176.073	-174.809	-176.111	-176.05	-176.198	-176.038	-176.381	-176.143	-176.189	-176.471	-175.836	-197.343	-175.538	-176.016	-176.052	NaN	-176.149	-177	-176.036
VH-Y101	-10.16	-10.688	-27.642	-8.596	-12.152	-174.496	-31.18	-9.027	-7.06	-0.713	-10.951	-9.415	-10.277	-9.706	-6.62	-8.825	-8.939	-8.077	-12.149	NaN
VH-Y102	-176.057	-176.768	-177.252	-174.762	-202.618	-174.939	-177.114	-193.309	-172.747	-168.303	-176.785	-177.507	-174.464	-175.182	-192.57	-173.976	-173.512	-174.441	-177.751	NaN
VH-D103	-178.765	-179.814	NaN	-176.708	-173.117	-179.358	-179.242	-181.105	-170.904	-179.067	-199.335	-200.015	-180.284	-177.405	-175.99	-178.47	-200.213	-179.719	-179.264	-170.047
VH-Y104	-230.031	-224.324	-222.972	-222.685	-189.223	-232.805	-202.923	-224.735	-206.319	-215.838	-234.074	-222.47	-231.479	-223.754	-204.328	-228.106	-223.651	-224.628	-169.489	NaN

Supplementary Table 3. The $\Delta\Delta G$ values for Cluster 2 of $EGFR^{S492R}$/ $Cetuximab^{Mut}$, Related to Figure 3.

Residue	A	C	D	E	F	G	H	I	K	L	M	N	P	Q	R	S	T	V	W	Y
VL-D1	47.148	47.206	NaN	47.228	47.02	46.959	47.198	47.54	46.801	47.347	47.203	45.586	46.561	47.565	48.065	47.245	47.532	47.537	46.184	47.083
VL-I2	43.764	43.816	42.087	44.833	47.053	42.809	48.176	NaN	51.354	45.467	45.489	43.519	43.481	44.152	52.094	43.863	43.891	43.45	46.739	47.915
VL-Q27	45.063	45.023	45.286	42.848	46.091	45.256	45.554	44.776	47.163	46.038	42.481	45.574	45.196	NaN	46.562	45.216	45.098	44.892	45.387	45.769
VL-S28	43.721	43.707	43.652	44.091	43.731	43.694	43.69	43.485	43.604	43.599	43.712	43.744	43.744	42.653	43.611	NaN	43.494	43.364	43.759	43.74
VL-N32	-138.857	-136.402	-137.546	-139.063	-137.594	-138.463	-138.114	-134.549	-137.155	-137.729	-141.78	NaN	-126.896	-138.866	-136.702	-138.009	-140.332	-142.768	-139.069	-141.549
VL-H34	-130.478	-130.517	-130.526	-130.824	-128.552	-130.586	NaN	-129.978	-128.118	-131.834	-130.204	-128.813	-130.837	-130.298	-130.488	-130.473	-130.493	-130.091	-129.594	-129.557
VL-K49	-131.334	-131.652	-132.165	-126.946	-133.275	-131.63	-132.543	-133.814	NaN	-132.2	-131.303	-133.257	-126.381	-130.259	-130.563	-125.694	-132.4	-132.345	-126.486	-131.406
VL-Y50	40.126	39.55	39.317	40.176	41.787	40.84	42.131	39.888	43.772	39.659	38.951	41.446	40.104	40.461	43.116	40.172	40.201	39.679	41.191	NaN
VL-E53	44.301	43.999	43.371	NaN	43.662	44.179	44.206	46.62	45.359	43.082	43.685	44.155	44.489	43.607	43.29	44.304	44.111	44.067	41.185	44.423
VL-N91	-111.457	-111.086	-111.039	-122.758	-127.095	-164.898	-113.235	-112.636	-112.186	-131.671	-102.681	NaN	-124.868	-109.205	-94.444	-111.449	-110.586	-111.483	-33.584	-105.582
VL-N92	4.047	3.661	4.432	4.781	3.003	-139.786	4.338	-146.631	6.101	173.856	2.16	NaN	-145.96	3.724	4.799	3.828	3.612	3.973	7.673	3.096
VL-N93	4.713	3.937	3.17	3.48	9.274	5.091	5.92	3.952	7.512	3.95	2.438	NaN	9.098	5.281	9.202	5.119	4.454	4.048	16.63	11.058
VL-W94	-25.141	-25.618	-23.921	-21.327	-151.203	-23.827	-25.537	-90.71	-1.563	6.471	3.756	-153.491	57.516	-20.424	-17.585	-23.436	-22.321	-25.49	NaN	-21.23
VL-P95	-147.189	-148.034	-149.072	-147.751	-146.514	-137.906	-144.826	-148.776	-134.96	-35.781	-66.036	-146.313	NaN	-143.346	-138.203	-146.281	-147.226	-147.693	-144.578	-145.55
VL-T96	-152.752	-141.004	-150.397	-149.313	-138.09	-138.59	-142.572	-144.74	-146.517	-148.778	-132.065	-145.634	-146.475	-145.313	-149.772	-146.246	NaN	-111.49	-148.323	-145.383
VH-N31	43.402	43.445	43.263	42.28	43.411	43.459	43.292	43.915	43.463	43.267	43.073	NaN	43.452	43.053	43.025	43.417	43.441	44.028	43.138	43.386
VH-H35	-128.957	-130.051	-130.496	-129.895	-127.699	-128.595	NaN	-129.864	-129.912	-128.163	-130.461	-130.841	-129.353	-129.784	-129.897	-129.055	-128.969	-130.943	-126.459	-128.537
VH-W47	-134.818	-128.399	-133.099	-125.118	-133.438	-147.702	-118.128	-132.983	-124.678	-129.763	-126.778	-132.507	-122.653	-124.914	-141.191	-129.107	-125.45	-125.577	NaN	-117.402
VH-G49	41.793	42.404	42.055	42.202	41.776	NaN	42.599	41.626	40.478	41.195	41.322	41.934	41.612	41.737	41.351	42.049	43.191	41.475	41.971	41.662
VH-V50	-126.013	-125.665	-119.824	-126.965	-128.54	-110.763	-125.356	-117.88	-125.135	-122.314	-122.912	-123.068	-121.581	-116.193	-127.737	-126.936	-123.434	NaN	-125.166	-125.591
VH-W52	44.691	44.214	44.827	45.097	44.306	44.889	42.796	44	47.321	45.681	42.804	45.084	44.695	42.475	44.708	44.484	43.938	43.654	NaN	44.817
VH-S53	40.965	40.967	41.524	40.857	48.829	41.091	48.596	43.112	58.694	51.56	53.597	46.134	40.979	40.828	41.656	NaN	40.709	42.346	47.183	55.932
VH-G54	48.654	59.871	67.439	80.908	86.46	NaN	79.698	149.756	171.059	516.842	102.055	69.57	55.783	75.352	151.704	48.064	89.991	139.793	140.416	99.267
VH-G55	40.795	41.009	39.516	40.888	40.241	NaN	40.955	40.787	41.034	40.482	41.039	41.099	63.465	40.634	41.089	41.399	40.905	40.752	38.203	40.171
VH-N56	42.936	45.848	46.655	51	104.641	43.968	46.2	46.746	52.971	76.628	44.925	NaN	42.492	50.216	53.401	43.641	49.106	47.665	44.445	45.425
VH-T57	42.899	42.883	43.456	42.983	41.83	42.835	43.151	42.739	42.967	42.911	42.934	43.068	45.929	42.935	41.853	43.107	NaN	42.818	42.442	42.848
VH-D58	-146.196	-25.533	NaN	-142.82	-131.13	-147.623	-63.024	-140.415	-141.18	-143.204	-26.682	-147.359	13.645	-143.73	-140.265	-146.665	-20.288	-139.447	-134.281	-98.399
VH-Y59	42.036	42.051	42.204	42.123	42.081	41.823	42.127	42.08	42.019	42.023	41.998	41.973	42.343	41.958	40.878	42.151	42.14	42.081	42.071	NaN
VH-N60	43.396	40.368	40.459	41.247	40.772	40.539	43.31	78.44	43.34	39.987	40.495	NaN	61.05	41.409	41.435	43.321	51.062	78.286	43.211	43.331
VH-T100	-129.627	-128.435	-127.815	-128.866	-129.555	-128.301	-129.145	-130.029	-128.995	-128.985	-129.161	-128.649	-127.412	-129.631	-130.633	-128.276	NaN	-128.322	-130.075	-129.493
VH-Y101	45.128	44.421	48.299	46.638	42.9	-128.057	44.652	46.961	48.212	54.346	44.795	46.18	44.969	45.713	48.966	46.095	48.644	47.542	53.153	NaN
VH-Y102	-139.534	-139.165	-138.334	-138.779	-143.644	-137.292	-140.613	-110.34	-134.404	-124.539	-137.61	-139.921	-117.246	-137.084	-130.158	-137.695	-130.304	-132.02	-134.601	NaN
VH-D103	-132.242	-126.227	NaN	-135.386	-97.603	-129.59	-120.178	-138.85	-119.538	-114.001	-137.713	-143.061	-136.862	-123.634	-128.273	-128.207	-132.96	-126.947	-116.87	-89.441
VH-Y104	-171.437	-167.738	-167.975	-159.597	-157.337	-171.116	-149.989	-167.434	-151.613	-159.15	-156.16	-165.144	-167.773	-159.806	-137.387	-169.874	-168.159	-170.894	-93.743	NaN
VH-E105	-131.001	-131.002	-130.76	NaN	-126.488	-130.991	-129.154	-130.888	-131.684	-131.606	-123.229	-131.03	-130.779	-130.954	-127.694	-131.011	-131.03	-130.966	-128.355	-126.067

Supplementary Table 4. The $\Delta\Delta G$ values for Cluster 3 of $EGFR^{S492R}$/$Cetuximab^{Mut}$, Related to Figure 3.

Residue	A	C	D	E	F	G	H	I	K	L	M	N	P	Q	R	S	T	V	W	Y
VL-D1	-73.614	-74.037	NaN	-74.484	-73.616	-73.792	-71.773	-73.931	-73.678	-73.721	-73.74	-75.02	-73.548	-73.695	-73.573	-73.568	-73.796	-73.926	-73.724	-73.593
VL-S26	-71.195	-71.187	-71.154	-71.229	-71.149	-71.222	-71.192	-71.157	-71.192	-71.147	-71.146	-71.204	-71.22	-71.153	-71.145	NaN	-71.16	-71.126	-71.153	-71.158
VL-Q27	-68.029	-68.489	-65.882	-70.411	-67.756	-67.49	-66.472	-59.353	-64.376	-67.846	-69.386	-66.757	-65.498	NaN	-65.418	-67.467	-68.332	-50.74	-67.135	-67.449
VL-S28	-71.119	-71.33	-70.9	-69.694	-70.607	-70.725	-71.487	-69.495	-70.663	-70.639	-70.735	-71.357	-66.208	-70.824	-70.916	NaN	-71.507	-69.004	-70.497	-70.51
VL-T31	-70.835	-70.607	-70.889	-70.609	-70.567	-70.813	-70.611	-70.437	-70.611	-70.609	-70.611	-70.35	-70.706	-70.61	-70.608	-70.495	NaN	-70.543	-70.61	-69.85
VL-N32	-121.079	-121.943	-118.066	-117.994	-120.746	-120.431	-120.454	-122.532	-119.67	-116.991	-120.053	NaN	-121.927	-114.487	-117.286	-120.738	-120.769	-121.977	-113.328	-121.813
VL-H34	-114.221	-114.21	-114.165	-114.03	-114.25	-113.445	NaN	-114.115	-113.08	-114.268	-114.154	-114.251	-115.116	-114.291	-114.545	-114.264	-114.196	-114.076	-114.942	-114.292
VL-Y50	-69.657	-69.727	-72.919	-69.517	-70.902	-69.881	-69.85	-69.558	-67.954	-69.542	-70.479	-69.254	-69.282	-70.5	-67.46	-69.436	-69.533	-69.949	-70.736	NaN
VL-E53	-67.883	-67.806	-68.9	NaN	-66.689	-67.908	-67.635	-67.764	-67.196	-67.459	-67.634	-66.399	-67.506	-67.858	-67.851	-67.889	-67.87	-67.807	-67.369	-67.151
VL-N91	-128.244	-128.151	-128.081	-128.828	-123.722	-165.413	-126.398	-130.782	-128.415	-131.251	-129.382	NaN	-127.794	-128.467	-124.074	-127.84	-129.65	-130.11	5.305	-123.576
VL-N92	-83.712	-83.752	-82.016	-81.233	-82.244	-124.33	-82.246	-70.128	-80.725	-69.837	-86.264	NaN	-84.178	-82.749	-80.864	-84.425	-81.562	-82.284	-81.092	-81.669
VL-N93	-84.597	-85.111	-84.95	-81.323	-74.956	-84.776	-78.067	-66.846	-12.762	-63.149	-68.641	NaN	-78.848	-79.264	-50.291	-83.953	-76.986	-56.752	-45.111	-73.204
VL-W94	-89.273	-90.003	-85.791	-83.563	-84.63	-88.373	-83.842	-25.943	-42.249	-66.069	-66.731	-85.439	195.307	-83.076	-64.159	-86.608	-83.17	-75.79	NaN	-85.666
VL-P95	-86.408	-85.954	-85.613	-85.298	-83.252	-132.759	-85.358	-84.941	-36.657	-88.041	-85.7	-86.28	NaN	-85.917	-44.286	-86.158	-83.68	-85.358	-85.537	-85.162
VL-T96	-137.694	-137.751	-136.629	-129.642	-139.238	-136.986	-136.165	-133.938	-117.057	-122.689	-129.731	-134.918	-113.207	-125.061	-128.346	-137.239	NaN	-136.298	-139.338	-140.187
VH-T30	-70.608	-70.651	-70.608	-70.608	-70.652	-70.651	-70.608	-70.651	-70.608	-70.608	-70.608	-70.651	-70.647	-70.608	-70.608	-70.608	NaN	-70.651	-70.662	-70.652
VH-N31	-70.641	-70.559	-70.741	-70.631	-70.522	-70.57	-70.681	-70.756	-70.555	-70.572	-70.715	NaN	-70.581	-70.603	-70.444	-70.477	-70.526	-70.754	-70.829	-70.59
VH-W47	-151.683	-152.206	-152.719	-151.931	-151.233	-151.644	-151.002	-152.449	-149.842	-151.684	-151.125	-151.716	-151.367	-151.233	-145.507	-151.734	-151.937	-152.55	NaN	-151.324
VH-V50	-133.705	-133.67	-131.159	-134.763	-29.929	-135.354	-134.357	-136.079	-116.753	-133.59	-133.094	-133.26	-118.943	-128.797	-127.337	-133.059	-138.191	NaN	23.735	-29.399
VH-W52	-66.723	-68.155	-65.963	-66.421	-69.683	-66.096	-70.779	-65.283	-65.707	-58.045	-68.651	-67.864	-68.329	-68.054	-67.986	-66.579	-67.507	-65.432	NaN	-71.038
VH-S53	-70.738	-70.353	-70.68	-67.662	-23.496	-70.317	-49.997	-61.898	-49.11	-59.567	-48.893	-65.571	-69.635	-49.552	-27.94	NaN	-67.198	-63.048	-27.829	-30.985
VH-G54	-59.816	-46.82	-45.601	-35.157	5.03	NaN	-26.098	-21.933	-29.442	-13.495	-37.708	-43.473	-53.428	-36.601	-34.169	-57.536	-55.299	-25.935	61.974	10.774
VH-G55	-70.953	-71.088	-71.439	-71.544	-71.825	NaN	-71.36	-71.023	-70.505	-72.031	-70.972	-71.172	-64.637	-70.91	-70.22	-71.066	-70.853	-71.046	-70.261	-71.846
VH-N56	-71.988	-67.195	-64.838	-53.231	20.265	-70.537	-46.737	-66.865	-48.142	-15.064	-45.371	NaN	-68.864	-40.581	-51.825	-72.266	-50.833	-63.147	-61.79	-58.108
VH-T57	-70.958	-70.893	-70.062	-71.103	-70.653	-71.143	-70.362	-70.318	-70.897	-71.099	-71.052	-70.642	-69.93	-71.044	-70.935	-70.95	NaN	-71.02	-70.902	-72.259
VH-D58	-99.119	-89.539	NaN	-81.662	-20.222	-104.274	-66.297	-78.681	-80.431	-56.664	-85.683	-81.888	-93.138	-82.215	-71.599	-97.048	-90.15	-89.169	339.214	17.652
VH-Y59	-70.648	-70.624	-70.378	-70.57	-70.541	-78.167	-70.453	-70.506	-70.575	-70.629	-70.632	-70.528	-74.433	-70.933	-70.541	-70.433	-70.451	-70.521	-70.467	NaN
VH-N60	-77.884	-78.604	-78.37	-78.722	-76.728	-79.632	-77.782	-76.855	-77.413	-77.088	-76.651	NaN	-77.73	-78.737	-77.878	-78.366	-74.354	-55.649	-77.663	-77.234
VH-T61	-78.307	-76.622	-78.171	-78.353	-78.321	-78.244	-78.194	-74.175	-76.401	-78.547	-77.694	-78.291	-76.91	-78.216	-78.286	-77.894	NaN	-76.984	-78.406	-78.425
VH-T64	-70.664	-70.57	-70.938	-71.647	-70.283	-70.709	-70.662	-70.203	-70.248	-70.367	-70.051	-70.595	-70.83	-71.098	-70.657	-70.663	NaN	-70.551	-70.488	-70.254
VH-T100	-114.763	-114.671	-112.456	-115.863	-115.193	-114.573	-114.78	-114.996	-114.693	-115.022	-114.84	-114.487	-114.172	-114.393	-114.527	-114.732	NaN	-114.713	-115.857	-115.119
VH-Y101	-68.347	-68.888	-66.454	-63.567	-70.249	-111.213	-69.382	-65.842	-66.194	-47.473	-69.149	-68.221	-69.004	-65.884	-66.405	-66.957	-67.76	-64.518	-68.604	NaN
VH-Y102	-118.474	-118.87	-118.465	-117.696	-124.175	-116.893	-120.228	-119.906	-114.758	-119.368	-119.499	-119.115	-115.328	-118.643	-115.441	-117.101	-115.913	-120.539	-86.918	NaN
VH-D103	-122.063	-121.469	NaN	-120.058	-99.913	-121.091	-116.262	-122.767	-118.111	-120.876	-123.947	-122.222	-123.763	-121.967	-120.183	-121.51	-121.88	-121.983	-118.521	-101.597
VH-Y104	-168.781	-168.236	-168.583	-166.852	-138.081	-170.115	-144.4	-168.901	-147.694	-145.482	-163.778	-165.321	-169.464	-166.43	-132.462	-168.059	-168.158	-169.697	-110.333	NaN

Supplementary Table 5. The $\Delta\Delta G$ values for Cluster 1 of $EGFR^{G465R}$/$Cetuximab^{Mut}$, Related to Figure 3.

Residue	A	C	D	E	F	G	H	I	K	L	M	N	P	Q	R	S	T	V	W	Y
VL-D1	2.19	40.365	NaN	2.491	40.499	30.308	75.462	2.527	40.41	30.509	3.762	29.868	2.228	30.557	30.557	68.478	2.535	30.554	31.118	68.477
VL-I2	27.894	28.576	26.992	28.331	34.835	27.864	31.207	NaN	38.767	33.365	27.069	27.87	28.924	30.551	41.579	27.974	28.037	28.135	34.093	33.845
VL-S26	27.333	27.338	28.582	27.326	27.332	27.681	28.58	27.143	27.333	27.335	27.107	27.105	27.328	28.581	27.686	NaN	27.188	27.501	27.331	28.275
VL-Q27	28.597	27.77	29.335	25.275	29.67	31.418	30.569	28.2	33.808	28.73	29.583	30.881	31.148	NaN	31.098	29.017	29.699	29.371	29.389	29.928
VL-S28	37.09	27.32	27.747	38.791	27.473	75.219	27.524	29.237	75.12	37.301	28.958	37.306	75.254	-9.909	75.174	NaN	-10.214	28.878	28.585	44.689
VL-N32	-46.518	-7.678	-43.263	-6.445	1.457	43.488	48.671	40.969	-5.743	43.236	-9.198	NaN	-5.118	-47.531	-7.23	40.631	2.612	-11.037	39.076	38.307
VL-H34	41.101	39.705	57.008	41.41	-7.615	50.416	NaN	-5.394	-41.794	39.819	-43.037	-42.447	41.092	-36.717	5.616	41.542	-6.816	45.118	-4.732	-7.339
VL-Y50	64.005	-4.033	62.165	24.652	-3.13	34.228	26.741	24.841	8.72	34.596	41.265	62.717	-5.503	24.235	26.807	62.171	-3.745	24.147	19.27	NaN
VL-E53	43.98	0.716	65.01	NaN	44.368	36.978	27.335	44.222	28.036	37.008	27.502	27.495	69.569	37.025	65.759	27.128	1.288	65.075	27.43	27.225
VL-N91	24.078	-25.101	15.903	14.121	16.928	50.082	14.96	22.913	66.175	11.718	62.529	NaN	16.594	14.061	70.377	14.636	-25.052	-24.301	63.285	16.514
VL-N92	26.813	24.805	78.288	28.302	-8.602	26.705	75.806	82.628	74.825	238.172	-14.302	NaN	26.731	-11.767	27.378	27.769	-12.936	27.932	29.592	27.751
VL-N93	-6.44	25.165	24.22	27.955	31.717	28.042	-7.193	31.09	38.629	79.865	27.994	NaN	30.807	32.548	50.483	27.188	-10.763	28.834	-0.399	0.291
VL-W94	67.538	0.021	76.119	4.661	29.159	30.182	66.216	5.096	13.276	79.727	7.471	37.38	272.051	70.21	72.158	29.927	33.547	4.158	NaN	28.454
VL-P95	68.257	26.006	-6.743	25.508	26.898	37.372	25.794	-6.431	23.105	100.528	-7.178	68.066	NaN	25.972	-5.812	25.526	-4.791	-6.042	-3.295	-6.436
VL-T96	10.714	9.54	-25.137	59.033	59.488	18.658	68.313	18.542	51.63	19.259	-20.864	7.833	-24.633	12.197	-24.608	8.818	NaN	51.054	10.055	45.799
VH-N31	28.479	63.946	37.67	28.024	28.066	38.281	65.959	67.839	-0.111	27.926	72.651	NaN	37.82	65.855	28.164	44.874	0.226	67.576	28.721	0.228
VH-H35	5.25	34.783	-2.352	-10.489	6.642	-37.486	NaN	63.394	45.781	46.362	3.127	-2.275	-3.806	31.721	11.761	-30.725	-7.415	29.614	38.803	16.144
VH-W47	32.595	-3.919	-4.921	8.167	12.854	-3.683	-3.733	-3.002	-29.444	33.631	-8.254	-33.42	-33.104	7.322	32.986	-33.149	2.993	-5.209	NaN	-3.865
VH-V50	-171.187	-157.271	-160.727	-116.021	-5.55	-127.854	-85.807	-160.268	-141.874	-64.583	-30.706	-86.452	-143.126	49.53	-29.791	-165.003	-128.615	NaN	-130.457	-139.676
VH-W52	-150.692	-144.066	-153.414	-112.932	-144.483	-153.305	-149.939	-126.204	-117.976	-125.965	-153.204	-179.323	-178.692	-124.738	-118.596	-116.146	-153.517	-144.625	NaN	-76.225
VH-S53	10.093	3.222	4.441	29.019	-13.899	-34.77	2.923	-35.279	1.216	36.987	38.161	5.191	-35.104	-5.912	38.715	NaN	-6.765	2.86	104.459	19.655
VH-G54	4.784	8.987	15.633	37.429	24.203	NaN	70.563	81.868	177.263	574.252	53.393	45.314	38.303	17.083	161.523	-0.079	46.207	93.23	83.208	77.786
VH-G55	33.485	-33.745	33.149	31.45	-6.597	NaN	31.706	-2.737	35.039	-4.101	-3.947	7.344	-29.214	-35.038	31.776	34.862	-30.493	-33.512	39.738	5.498
VH-N56	-72.322	-21.983	-95.702	-91.268	118.568	-25.278	-91.625	-50.708	-51.598	-49.366	76.319	NaN	-60.837	-92.784	-50.088	24.482	-94.953	-88.975	-93.216	-93.948
VH-T57	-96.101	-67.631	-58.111	-95.341	-58.401	-65.165	-57.917	-96.803	-96.312	-93.184	-68.53	-66.661	-65.594	-68.246	-30.329	-30.483	NaN	-93.341	-67.892	-58.74
VH-D58	-79.038	-115.596	NaN	-78.875	-81.897	-88.31	-46.111	-121.28	-76.357	-89.094	-118.767	-77.494	-115.116	-81.107	-76.302	-88.364	-79.21	-87.694	-46.551	-56.235
VH-Y59	66.317	38.278	28.53	0.204	0.549	29.491	38.08	73.237	-0.066	37.907	28.101	28.695	34.621	1.339	28.95	37.714	28.077	-0.027	5.671	NaN
VH-T64	26.731	65.474	28.775	37.157	0.214	38.637	37.357	-0.571	37.388	1.459	27.527	67.033	27.37	27.76	28.168	28.2	NaN	27.837	-0.767	0.966
VH-A98	NaN	-5.039	-33.323	-33.557	33.183	-5.377	-5.388	3.654	-32.024	4.965	-4.677	7.241	-4.401	-33.503	6.469	-32.061	32.354	5.771	-4.273	35.323
VH-T100	32.859	4.317	-5.187	30.341	31.313	-7.219	-5.239	-4.423	-3.966	-8.713	-5.062	39.647	-3.202	4.73	-6.796	4.222	NaN	-5.806	-7.395	-6.973
VH-Y101	-29.74	35.172	3.344	-28.194	-2.778	16.758	-0.131	39.931	11.167	41.773	36.008	-28.694	-30.16	-27.312	-25.604	-2.483	-0.888	-30.199	-25.315	NaN
VH-Y102	-99.07	-61.99	-99.256	-100.058	-131.803	-126.138	-91.127	-120.959	-56.82	-50.237	-127.499	-128.204	-126.392	-103.277	-101.04	-98.058	-99.056	-122.629	-102.286	NaN
VH-D103	-4.349	39.644	NaN	0.313	-23.519	34.309	5.186	-7.142	2.008	6.5	-29.9	-5	38.665	-0.923	2.516	-29.524	-31.774	33.767	-3.034	-27.777
VH-Y104	-40.34	-35.607	-40.648	-32.85	-15.855	-37.033	2.38	38.093	46.059	-5.195	-34.758	38.864	-5.479	-2.814	43.106	14.946	37.995	37.615	-16.035	NaN
VH-E105	-5.522	-33.195	-6.727	NaN	0.162	32.684	34.36	-4.233	33.631	-2.909	-34.826	33.16	33.886	-4.431	-30.3	-33.839	-4.617	-34.292	-31.494	-2.964
VH-F106	-29.076	-29.482	0.277	6.173	NaN	10.876	-2.332	-30.97	7.292	34.588	-30.443	37.428	-3.312	-0.525	-31.225	1.365	-2.914	-32.328	21.333	13.386

Supplementary Table 6. The $\Delta\Delta G$ values for Cluster 2 of EGFR^{G465R}/ Cetuximab^{Mut}, Related to Figure 3.

Residue	A	C	D	E	F	G	H	I	K	L	M	N	P	Q	R	S	T	V	W	Y
VL-D1	2.564	2.767	NaN	2.198	2.65	3.321	3.483	2.561	4.354	2.662	2.395	2.076	2.794	3.227	2.911	2.773	2.56	2.768	1.441	2.71
VL-I2	-2.162	-2.181	0.233	-0.285	-0.423	-1.635	3.801	NaN	2.233	-3.539	0.751	-2.334	-2.293	5.564	8.087	-1.778	-1.78	-4.652	-0.378	1.459
VL-Q27	-0.438	-1.303	-0.387	-1.819	0.896	-1.257	0.756	5.903	1.836	0.456	-1.039	2.96	-1.37	NaN	1.671	-0.934	-0.549	0.308	1.71	0.812
VL-S28	-0.732	-0.844	-0.705	-0.011	-0.711	-0.892	-0.767	-0.817	-0.77	-0.758	-0.653	-0.754	-0.776	-0.528	-1.026	NaN	-0.914	-1.215	-0.909	-0.696
VL-N32	-35.771	-36.326	-35.057	-35.982	-38.627	-35.457	-33.427	-37.293	-33.796	-36.738	-37.461	NaN	-35.445	-36.222	-33.649	-35.38	-36.13	-36.833	-38.822	-39.169
VL-H34	-14.925	-15.987	-17.269	-36.9	-33.973	-36.92	NaN	-34.88	-14.302	-31.478	-33.799	-15.655	-35.114	-19.916	-13.405	-16.355	-30.727	-36.952	-35.608	-35.092
VL-Y50	-3.43	-3.458	-4.274	-3.822	-3.135	-3.43	-2.869	-3.501	-23.239	-3.808	-4.183	-3.638	-4.06	-3.871	-3.312	-3.424	-3.38	-3.684	-3.429	NaN
VL-E53	-1.15	-1.15	-1.192	NaN	-1.022	-1.15	-1.202	-1.15	-0.979	0.634	-1.082	-1.317	1.158	-1.41	1.742	-1.358	-1.15	-1.15	1.018	-1.097
VL-N91	-21.977	-45.515	-48.15	-37.438	-31.217	-46.102	-34.056	-39.161	-34.318	-51.293	-37.216	NaN	-42.025	-36.654	-22.444	-44.288	-38.126	-41.277	7.064	-10.879
VL-N92	-0.909	-0.874	-0.739	-0.159	-3.039	-1.07	-1.343	-0.641	-0.891	0.296	-3.344	NaN	-1.127	-1.294	-1.312	-0.786	-1.755	-1.083	-1.311	-2.183
VL-N93	-2.085	-2.604	-3.254	2.933	12.623	-1.437	-1.211	-1.736	3.748	-1.305	5.231	NaN	-0.017	2.455	4.764	-1.552	-2.054	-2.858	9.545	11.688
VL-W94	-17.132	-17.491	-14.534	-13.27	-16.432	-14.954	-18.288	-7.718	-4.442	-5.368	-10.964	-18.152	276.045	-13.082	-14.067	-15.455	-12.818	-11.068	NaN	-15.724
VL-P95	-21.733	-22.037	-21.438	-21.71	-21.551	-21.146	-21.754	-24.891	-25.096	25.498	-21.438	-21.482	NaN	-21.56	-19.964	-21.556	-21.031	-21.154	-20.47	-21.426
VL-T96	-53.782	-52.261	-42.822	-47.102	-7.999	-45.774	-39.372	-31.497	-41.32	-40.731	-51.203	-53.5	-52.772	-46.901	-42.759	-48.079	NaN	-47.55	-37.529	31.513
VH-N31	-0.346	-0.346	-0.346	-0.557	-0.34	-0.349	-0.354	-0.408	-0.347	-0.351	-0.316	NaN	-0.35	-0.387	-0.526	-0.347	-0.336	-0.405	-0.592	-0.364
VH-H35	-52.766	-52.739	-52.573	-52.963	-45.738	-50.157	NaN	-26.813	-51.505	-48.701	-51.9	-52.675	-49.668	-51.913	-57.592	-52.969	-51.922	-21.52	-46.932	-51.71
VH-W47	-40.459	-35.579	-35.387	-26.774	-38.41	-38.137	-38.376	-38.521	-41.487	-46.806	-36.92	-34.908	-38.731	-37.519	-26.506	-38.26	-38.99	-37.667	NaN	-38.432
VH-V50	-121.45	-115.184	-117.03	-104.606	-48.156	-121.231	-57.053	-116.072	-48.871	-111.527	-103.248	-116.183	-125.397	-57.384	-50.847	-119.902	-110.785	NaN	-110.466	-124.68
VH-W52	-119.402	-121.028	-119.579	-97.721	-95.556	-131.163	-97.252	-120.638	-90.477	-99.839	-99.755	-119.746	-119.648	-96.872	-91.662	-119.074	-120.535	-121.439	NaN	-91.277
VH-S53	-14.989	-14.522	-15.248	-14.296	-12.763	-15.085	-12.41	-15.52	-7.851	-14.576	-12.532	-13.763	-16.548	-10.603	-10.449	NaN	-14.723	-14.94	-13.027	-13.228
VH-G54	-12.743	-7.632	2.402	13.852	181.088	NaN	43.721	99.329	47.319	269.301	40.366	-0.645	-0.99	11.858	54.12	-12.109	22.163	79.867	65.544	176.852
VH-G55	-16.081	-15.439	-16.609	-15.866	-16.573	NaN	-15.647	-15.202	-15.351	-16.295	-14.728	-14.418	7.328	-16.324	-15.901	-14.409	-15.013	-15.214	-18.929	-16.291
VH-N56	-26.474	-23.368	-18.956	-16.341	10.204	-30.488	-67.676	-84.938	-15.169	26.55	-20.455	NaN	-25.387	-17.853	-19.564	-20.847	-17.698	-72.015	-72.443	-69.438
VH-T57	-22.009	-22.143	-21.523	-22.355	-21.894	-21.746	-21.873	-20.083	-21.586	-21.897	-22.016	-21.577	-17.567	-21.639	-21.664	-21.943	NaN	-22.058	-21.392	-21.661
VH-D58	-71.516	-71.534	NaN	-72.14	-59.434	-71.01	-71.949	-81.27	-69.657	-72.132	-73.12	-65.534	-74.241	-74.273	-70.171	-71.074	-72.129	-70.995	-67.273	-14.787
VH-T64	-0.024	-0.035	-0.047	-0.325	0.025	-0.024	-0.024	0.073	-0.074	0.084	0.292	-0.005	-0.971	0.951	0	-0.024	NaN	0.024	-0.185	-0.043
VH-T100	-34.14	-32.701	-34.627	-34.822	-32.786	-32.526	-34.064	-31.516	-32.382	-32.156	-34.306	-32.199	-34.005	-32.829	-33.508	-32.933	NaN	-31.12	-33.494	-32.415
VH-Y101	-12.925	-13.735	-9.481	-11.518	-14.803	-11.452	-12.667	-8.237	-10.35	24.069	-13.599	-11.617	-13.114	-12.829	-10.244	-11.895	-11.809	-11.543	-10.94	NaN
VH-Y102	-92.613	-93.498	-92.645	-91.385	-97.505	-91.196	-98.067	-87.674	-89.163	-92.681	-94.498	-92.758	-84.38	-95.898	-87.339	-90.738	-91.28	-89.489	-94.372	NaN
VH-D103	-51.237	-53.901	NaN	-44.301	-50.427	-50.823	-51.367	-38.859	-45.175	-45.755	-53.007	-53.167	-50.678	-48.351	-47.74	-52.656	-53.559	-53.991	-50.22	-46.064
VH-Y104	-55.529	-51.304	-54.137	-50.915	-41.153	-55.716	-28.71	-52.019	-18.898	-48.083	-44.551	-48.881	-53.542	-46.719	1.041	-54.215	-51.92	-52.005	333.584	NaN
VH-E105	-35.747	-36.222	-37	NaN	-34.149	-35.807	-34.528	-35.519	-34.752	-28.774	-34.718	-35.93	-25.458	-34.639	-33.783	-26.969	-35.878	-36.557	-33.638	-41.896
VH-F106	-31.836	-33.437	-30.771	-31.78	NaN	-31.598	-29.017	-34.559	-28.155	-34.172	-32.11	-31.671	-35.734	-33.082	-33.047	-31.589	-30.888	-32.24	-0.415	-7.879

Supplementary Table 7. The $\Delta\Delta G$ values for Cluster 3 of EGFR^{G465R}/ Cetuximab^{Mut}, Related to Figure 3.

Residue	A	C	D	E	F	G	H	I	K	L	M	N	P	Q	R	S	T	V	W	Y
VL-D1	3.803	45.572	NaN	67.777	4.101	67.465	46.006	31.977	3.897	46.601	-31.917	44.284	3.872	3.882	-32.143	3.813	4.204	4.181	45.216	47.077
VL-I2	0.274	0.123	-0.119	2.897	1.671	0.241	2.466	NaN	7.587	7.018	2.83	-0.303	0.577	11.021	8.767	0.276	1.274	0.577	1.507	1.361
VL-Q27	-0.513	-0.553	-1.547	-1.621	0.366	0.087	1.368	-0.606	2.666	0.657	-1.404	1.079	-0.326	NaN	1.577	-0.12	-0.086	1.101	0.379	0.542
VL-S28	43.001	-0.012	0.188	-35.371	-36.253	-36.259	0.091	-36.293	-36.274	3.069	0.248	0.123	0.058	0.393	41.544	NaN	-0.039	42.847	0.019	42.265
VL-N32	-62.701	-20.215	-18.473	-19.555	-20.987	-19.112	-20.243	-21.097	-18.743	29.291	-21.128	NaN	-19.729	-63.139	-17.27	-19.247	28.286	-20.77	-21.66	-23.055
VL-H34	-18.473	-18.532	-58.766	-18.369	-59.288	-18.301	NaN	50.072	-18.143	-61.717	-18.594	-18.315	-61.015	-18.729	28.203	-12.763	-13.051	-12.156	-18.52	50.523
VL-Y50	-39.91	-39.921	-3.424	-4.573	37.327	-3.821	37.75	-3.722	3.168	38.151	59.129	37.988	-41.869	2.48	-3.54	37.934	2.11	23.905	-4.259	NaN
VL-E53	-38.064	-1.847	-1.645	NaN	39.7	-1.948	62.327	3.944	-1.693	-1.925	39.811	39.835	-0.006	-1.823	-37.888	-1.8	-38.111	39.749	-37.829	3.914
VL-N91	-18.108	25.779	-22.743	-68.302	-63.604	-44.06	-23.207	-25.003	-20.438	22.065	-27.07	NaN	-24.086	-24.802	-7.144	-23.169	-23.656	25.271	-13.801	-65.87
VL-N92	-0.092	64.15	0.571	1.276	2.953	-0.346	39.261	-0.384	0.114	49.806	61.864	NaN	-0.531	-36.319	42.835	2.791	2.525	-0.266	0.492	-1.522
VL-N93	-32.939	-0.305	-0.912	27.662	69.249	-0.168	10.96	0.308	6.316	3.047	-36.582	NaN	-0.039	1.33	7.898	-33.344	37.907	0.211	-26.019	6.241
VL-W94	-117.821	-81.654	-77.575	-35.382	-82.297	-115.802	-40.235	-75.874	-68.74	-108.347	-76.753	-82.33	215.203	-77.833	-78.037	-74.631	-77.208	-36.246	NaN	-80.362
VL-P95	-83.684	-83.791	-123.16	-83.885	-83.581	-83.724	-83.344	-76.548	-37.341	-47.107	-83.433	-83.605	NaN	-38.661	-76.635	-77.5	-84.213	-82.969	-79.418	-83.547
VL-T96	-77.297	-83.514	-87.25	-83.428	4.909	-85.141	-67.976	-83.098	-42.211	-88.509	-38.45	-85.832	-123.859	-83.55	-88.769	-79.299	NaN	-83.17	57.308	-11.3
VH-N31	-0.202	5.13	41.84	-0.173	41.821	-0.208	-0.171	-36.529	-0.204	-0.186	41.414	NaN	27.519	-0.266	-36.374	-36.433	42.519	5.142	-36.632	-0.2
VH-H35	-82.006	-117.464	-80.574	-71.464	-67.374	-119.29	NaN	-109.751	-110.004	-50.739	-78.575	-80.232	-39.708	-113.224	-78.609	-81.471	-28.025	-73.719	-32.114	-108
VH-W47	-35.316	-36.056	28.326	-35.903	-29.747	-35.457	9.951	8.532	-31.256	-42.296	-72.636	-71.693	7.191	7.958	10.323	-35.445	5.721	-7.515	NaN	-69.772
VH-V50	-95.567	-131.309	-95.314	-135.691	-52.299	-96.581	-73.834	-89.503	-126.725	-138.093	-90.998	-52.978	-56.271	-101.634	-87.366	-31.621	-96.696	NaN	-88.453	-194.855
VH-I51	-55.379	-55.619	8.345	-55.418	-13.653	-55.439	-50.199	NaN	-55.394	-91.645	-55.445	-55.478	-52.851	-13.863	-13.725	-55.387	-13.81	-55.559	-55.416	-52.778
VH-W52	-174.054	-218.377	-217.171	-216.782	-221.42	-216.496	-202.023	-216.422	-137.418	-214.014	-212.503	-253.06	-252.422	-214.095	-194.546	-216.427	-254.644	-246.995	NaN	-215.06
VH-S53	-6.584	-85.473	-49.101	-85.452	-43.978	-49.004	11.602	-7.953	-3.491	-6.558	-42.584	-48.135	-87.299	-7.109	-46.645	NaN	-48.87	-85.814	-28.736	-17.46
VH-G54	-41.394	-41.974	-34.326	8.809	-14.572	NaN	31.692	48.637	68.621	495.641	-56.9	-26.69	-37.334	11.787	84.124	-40.622	-0.451	36.038	41.851	-36.683
VH-G55	-87.496	-50.765	12.131	-9.325	-50.861	NaN	-50.863	-8.829	-50.497	-50.997	-86.52	-50.21	-44.651	-50.913	-51.257	-50.254	-50.11	-8.378	-53.869	-87.265
VH-N56	-107.93	-99.118	-93.832	-95.644	60.549	-149.743	-95.783	-111.175	-94.909	-62.311	-36.929	NaN	-100.815	-96.952	-95.033	-102.777	-95.81	-80.316	4.174	-96.863
VH-T57	-89.159	-125.291	-93.604	-83.75	-129.175	-88.923	-88.87	-93.059	-129.183	-93.067	-129.356	-129.204	-53.583	-93.166	-125.133	-51.551	NaN	-89.355	-88.664	-50.634
VH-D58	-54.628	-100.538	NaN	-100.511	-90.661	-129.226	-126.268	-86.488	-98.226	-63.334	-60.232	-53.638	-72.805	-99.887	-98.534	-77.957	-86.038	-48.016	-81.477	-36.688
VH-T64	-0.019	5.57	-0.044	42.42	0.027	-36.283	-0.019	-36.225	0.066	0.04	42.636	-0.048	-0.403	42.368	-36.461	-0.019	NaN	-36.243	-36.218	5.73
VH-T100	-18.355	-18.038	-18.596	-19.546	-54.338	23.377	-18.029	-53.003	-18.066	-54.096	9.39	23.603	-16.494	-17.8	-19.122	23.743	NaN	-17.439	-55.11	23.375
VH-Y101	22.004	-42.915	-38.917	0.762	-43.955	-40.666	-42.489	-39.368	-39.91	-10.916	-42.618	-41.018	-42.111	21.982	-39.908	23.016	-41.423	-0.782	-34.277	NaN
VH-Y102	-111.258	-106.465	-110.943	-112.549	-73.879	-109.979	-71.424	-104.994	-106.861	-109.619	-147.933	-69.499	-70.02	-69.749	-43.155	-110.481	-106.519	-106.403	-109.234	NaN
VH-D103	-71.709	-78.715	NaN	-74.116	-76.494	-76.774	-112.061	-74.801	-70.525	-74.391	-77.208	-114.114	-123.815	-74.354	-72.222	-113.524	-35.964	-121.901	-110.744	-108.141
VH-Y104	-96.602	-95.292	-131.587	-90.997	-90.667	-97.314	-39.938	-88.061	-121.083	-87.216	-125.59	-89.698	-51.742	-88.644	-87.781	-51.688	-130.784	-93.984	-87.087	NaN
VH-E105	-22.212	-27.358	14.142	NaN	-25.842	13.981	-25.282	-27.904	-27.589	-27.331	13.326	-27.297	-62.32	15.015	-23.405	-27.408	-27.325	-27.67	-21.305	9.08
VH-F106	-31.321	-31.521	-67.597	-31.402	NaN	-67.598	-31.324	10.064	-36.588	-31.576	10.145	-31.383	-75.658	-31.423	-32.56	-31.393	-31.414	-67.487	11.254	-28.493

Supplementary Table 8. All kon, koff, and KD values in three technical replicates, Related to Fig. 4.

	Experiment #1			Experiment #2			Experiment #3		
	Avidity to WT-EGFR-ECD-Fc			Avidity to WT-EGFR-ECD-Fc			Avidity to WT-EGFR-ECD-Fc		
	Kon	Koff	KD	Kon	Koff	KD	Kon	Koff	KD
	(1/Ms)	(1/s)	(M)	(1/Ms)	(1/s)	(M)	(1/Ms)	(1/s)	(M)
Cetuximab	2.87X10^{5}	1.17X10^{-4}	0.41X10^{-9}	1.38X10^{5}	9.26X10^{-5}	0.67X10^{-9}	8.03X10^{4}	9.06X10^{-6}	0.11X10^{-9}
Ctx-VY	5.24X10^{4}	2.10X10^{-4}	4.00X10^{-9}	4.57X10^{4}	4.44X10^{-5}	0.97X10^{-9}	7.49X10^{4}	1.33X10^{-4}	1.77X10^{-9}
Ctx-Y104D	8.06X10^{4}	5.17X10^{-4}	6.54X10^{-9}	6.05X10^{4}	1.26X10^{-4}	2.08X10^{-9}	1.05X10^{5}	2.79X10^{-4}	2.66X10^{-9}
Ctx-W52D	1.37X10^{5}	1.35X10^{-4}	0.99X10^{-9}	8.21X10^{4}	5.80X10^{-5}	0.71X10^{-9}	1.05X10^{5}	7.03X10^{-5}	0.67X10^{-9}
	Avidity to S492R-EGFR-ECD-Fc			Avidity to S492R-EGFR-ECD-Fc			Avidity to S492R-EGFR-ECD-Fc		
	Kon	Koff	KD	Kon	Koff	KD	Kon	Koff	KD
	(1/Ms)	(1/s)	(M)	(1/Ms)	(1/s)	(M)	(1/Ms)	(1/s)	(M)
Cetuximab	N.D.*	N.D.	N.D.	N.D.	N.D.	N.D.	N.D.	N.D.	N.D.
Ctx-VY	1.50X10^{5}	5.53X10^{-4}	3.68X10^{-9}	8.74X10^{4}	1.47X10^{-4}	1.68X10^{-9}	9.21X10^{4}	1.40X10^{-4}	1.52X10^{-9}
Ctx-Y104D	1.12X10^{5}	4.07X10^{-4}	3.63X10^{-9}	9.52X10^{4}	1.07X10^{-4}	1.13X10^{-9}	2.41X10^{5}	2.96X10^{-4}	1.23X10^{-9}
	Avidity to G465R-EGFR-ECD-Fc			Avidity to G465R-EGFR-ECD-Fc			Avidity to G465R-EGFR-ECD-Fc		
	Kon	Koff	KD	Kon	Koff	KD	Kon	Koff	KD
	(1/Ms)	(1/s)	(M)	(1/Ms)	(1/s)	(M)	(1/Ms)	(1/s)	(M)
Cetuximab	N.D.	N.D.	N.D.	N.D.	N.D.	N.D.	N.D.	N.D.	N.D.
Ctx-W52D	3.11X10^{5}	1.05X10^{-3}	3.39X10^{-9}	1.47X10^{4}	6.82X10^{-4}	4.64X10^{-9}	9.73X10^{4}	5.49X10^{-4}	5.64X10^{-9}

*N.D., not determined

Supplementary Table 9. Primers used in this study

Gene name	primer sequence
WT-EGFR	Overlap-F: TTTGGGACCTCCGGTCAGAAAACCAAAATTATAAGCAACAGAGGTGAAAACAGCTGCAA<br>Overlap-R: TTGCAGCTGTTTTCACCTCTGTTGCTTATAATTTTGGTTTTCTGACCGGAGGTCCCAAA
S492R-EGFR	Overlap-F: TTTGGGACCTCCGGTCAGAAAACCAAAATT AAGAGAAACA GAGGTGAAAA CAGCTGCAA<br>Overlap-R: TTGCAGCTGTTTTCACCTCTGTTTCTCTTAATTTTGGTTTTCTGACCGGAGGTCCCAAA
I491M-EGFR	Overlap-F: TTTGGGACCTCCGGTCAGAA AACCAAAATT ATGAGCAACA GAGGTGAAAA CAGCTGCAA<br>Overlap-R: TTGCAGCTGTTTTCACCTCTGTTGCTCATAATTTTGGTTTTCTGACCGGAGGTCCCAAA
K489E-EGFR	Overlap-F: TTTGGGACCTCCGGTCAGAAAACCGAGATTATAAGCAACA GAGGTGAAAA CAGCTGCAA<br>Overlap-R: TTGCAGCTGTTTTCACCTCTGTTGCTTATAATCTCGGTTTTCTGACCGGAGGTCCCAAA
K467T-EGFR	Overlap-F: GGAGATAAGTGATGGAGATGTGATAATTTCAGGAAACACCAATTTGTGCTATGCAAATA<br>Overlap-R: TATTTGCATAGCACAAATTGGTGTTTCCTGAAATTATCACATCTCCATCACTTATCTCC
G465R-EGFR	Overlap-F: GGAGATAAGTGATGGAGATGTGATAATTTCAAGAAACAAAAATTTGTGCTATGCAAATA<br>Overlap-R: TATTTGCATAGCACAAATTTTTGTTTCTTGAAATTATCACATCTCCATCACTTATCTCC
G465E-EGFR	Overlap-F: GGAGATAAGTGATGGAGATGTGATAATTTCAGAGAACAAAAATTTGTGCTATGCAAATA<br>Overlap-R: TATTTGCATAGCACAAATTTTTGTTCTCTGAAATTATCACATCTCCATCACTTATCTCC
S464L-EGFR	Overlap-F: GGAGATAAGTGATGGAGATGTGATAATTCTGGGAAACAAAAATTTGTGCTATGCAAATA<br>Overlap-R: TATTTGCATAGCACAAATTTTTGTTTCCCAGAATTATCACATCTCCATCACTTATCTCC
I462R-EGFR	Overlap-F: GGAGATAAGTGATGGAGATGTGAGAATTTCAGGAAACAAAAATTTGTGCTATGCAAATA<br>Overlap-R: TATTTGCATAGCACAAATTTTTGTTTCCTGAAATTCTCACATCTCCATCACTTATCTCC
R451C-EGFR	Overlap-F: CTGAACATAACATCCTTGGGATTATGCTCCCTCAAGGAGATAAGTGATGGAGAT<br>Overlap-R: ATCTCCATCACTTATCTCCTTGAGGGAGCATAATCCCAAGGATGTTATGTTCAG
S442R-EGFR	Overlap-F: ACATGGTCAGTTTTCTCTTGCAGTCGTCAGGCTGAACATAACATCCTTGGGATTACGCT<br>Overlap-R: AGCGTAATCCCAAGGATGTTATGTTCAGCCTGACGACTGCAAGAGAAAACTGACCATGT
V441D-EGFR	Overlap-F: ACATGGTCAGTTTTCTCTTGCAGTCGACAGCCTGAACATAACATCCTTGGGATTA CGCT<br>Overlap-R: AGCGTAATCCCAAGGATGTTATGTTCAGGCTGTCGACTGCAAGAGAAAACTGACCATGT
WT/MUT-EGFR	F: CCGGATATCATGCGACCCTCCGGGACGGCCGGGGCAGCGCTCCTGGCGCTGCTGGCTGC<br>R: CCGCTCGAGTCATGCTCCAATAAATTCACTGCTTTGTGGCGCGACCCTTAGGTATTCTG
WT/MUT-EGFR-eGFP	Overlap-F: ACAAAGCAGTGAATTTATTGGAGCAATGGTGAGCAAGGGCGAGGAGCT<br>Overlap-R: AGCTCCTCGCCCTTGCTCACCATTGCTCCAATAAATTCACTGCTTTGT<br>F: GGGGTACCATGCGACCCTCCGGGACGG<br>R: GCTCTAGAGCTCAAAGCTTCTTGTACAGCTCGT
WT/MUT-EGFR-ECD-Fc	Overlap-F: GGGCCTAAGATCCCGTCCATCGAGCCCAAATCTTGTGACAAA<br>Overlap-R: TTTGTCACAAGATTTGGGCTCGATGGACGGGATCTTAGGCCC<br>F: CTGGGACTGCTCCTGCTGTGGTTTCCCGGCGCCAGATGCCTGGAGGAAAAGAAAGTTTG<br>R: TTTATAGCGGCCGCTCATTTACCCGGAGACAGGGAGAGGCTCTTCTGCGTGTAGTGGTT
Ctx-Y104X	Overlap-F: GCGCTCTGACCTATTACGATNNSGAGTTCGCGTATTGGGGTCA<br>Overlap-R: TGACCCCAATACGCGAACTCSNNATCGTAATAGGTCAGAGCGC<br>F: GTGGCGGCCGCTCGAGATGGATATGAGGGTGCCTGCCCAGCTGCTGGGACTG<br>R: GCCCTCTAGACTCGAGTCATTTACCCGGAGACAGGGAGAGG
Ctx-W52D	Overlap-F: AAGGCCTGGAATGGCTGGGTGTAATCNNKAGCGGCGGTAACACCGACTACAAT<br>Overlap-R: GATTACACCCAGCCATTCCAGGCCTTTGCCAGGGCTCTGACGCACCC

	F: GTGGCGGCCGCTCGAGATGGATATGAGGGTGCCTGCCCAGCTGCTGGGACTG<br>R: GCCCTCTAGACTCGAGTCATTTACCCGGAGACAGGGAGAGG
Ctx-VY	Overlap-F1: AAGGCCTGGAATGGCTGGGTCAGATCTGGAGCGGCGGTAACAC<br>Overlap-R1: GTGTTACCGCCGCTCCAGATCTGACCCAGCCATTCCAGGCCTT<br>Overlap-F2: GCGCTCTGACCTATTACGATGTGGAGTTCGCGTATTGGGGTCA<br>Overlap-R2: TGACCCCAATACGCGAACTCCACATCGTAATAGGTCAGAGCGC<br>F: GTGGCGGCCGCTCGAGATGGATATGAGGGTGCCTGCCCAGCTGCTGGGACTG<br>R: GCCCTCTAGACTCGAGTCATTTACCCGGAGACAGGGAGAGG
Ctx-Y104D	Overlap-F1: CTGACCTATTACGATGATGAGTTCGCGTAT<br>Overlap-R1: ATACGCGAACTCATCATCGTAATAGGTCAG<br>F: GTGGCGGCCGCTCGAGATGGATATGAGGGTGCCTGCCCAGCTGCTGGGACTG<br>R: GCCCTCTAGACTCGAGTCATTTACCCGGAGACAGGGAGAGG
Ctx-DD	Overlap-F1: AAGGCCTGGAATGGCTGGGTGTAATCNNKAGCGGCGGTAACACCGACTACAAT<br>Overlap-R1: GATTACACCCAGCCATTCCAGGCCTTTGCCAGGGCTCTGACGCACCC<br>Overlap-F2: CTGACCTATTACGATGATGAGTTCGCGTAT<br>Overlap-R2: ATACGCGAACTCATCATCGTAATAGGTCAG<br>F: GTGGCGGCCGCTCGAGATGGATATGAGGGTGCCTGCCCAGCTGCTGGGACTG<br>R: GCCCTCTAGACTCGAGTCATTTACCCGGAGACAGGGAGAGG
Ctx-VWY	Overlap-F1: AAGGCCTGGAATGGCTGGGTCAGATCGACAGCGGCGGTAACAC<br>Overlap-R1: GTGTTACCGCCGCTGTCGATCTGACCCAGCCATTCCAGGCCTT<br>Overlap-F2: GCGCTCTGACCTATTACGATGTGGAGTTCGCGTATTGGGGTCA<br>Overlap-R2: TGACCCCAATACGCGAACTCCACATCGTAATAGGTCAGAGCGC<br>F: GTGGCGGCCGCTCGAGATGGATATGAGGGTGCCTGCCCAGCTGCTGGGACTG<br>R: GCCCTCTAGACTCGAGTCATTTACCCGGAGACAGGGAGAGG
Cetuximab-scFv library Related to S492R	F: ATGCGGCCCAGCCGGCCCAGGTTCAACTGAAACAGTCCGG<br>R: AAATATGCGGCCGCTTTCAGTTCCAGTTTCGTGCCGGCACCGAAAGT<br>Overlap-F1: CCTGGAATGGCTGGGTNNSATCTGGAGCGGCGGTAACACCNNSTACAATACCCCATTCA<br>Overlap-R1: TGAATGGGGTATTGTASNNGGTGTTACCGCCGCTCCAGATSNNACCCAGCCATTCCAGG<br>Overlap-F2: GCGCTCTGACCTATTACGATNNSGAGTTCGCGTATTGGGGTCAGGGCACTCTGGTTACC<br>Overlap-R2: GGTAACCAGAGTGCCCTGACCCCAATACGCGAACTCSNNATCGTAATAGGTCAGAGCGC<br>Overlap-F3: AGCTTTTCTTGCCGCGCGTCCCAAAGCATTGGTACCNNSATTCACTGGTACCAGCAGCG<br>Overlap-R3: CGCTGCTGGTACCAGTGAATSNNGGTACCAATGCTTTGGGACGCGCGGCAAGAAAAGCT<br>Overlap-F4: GCTGACTACTATTGTCAGCAGAACAATAACNNSCCGNNSACTTTCGGTGCCGGCACGAA<br>Overlap-R4: TTCGTGCCGGCACCGAAAGTSNNCGGSNNGTTATTGTTCTGCTGACAATAGTAGTCAGC
Cetuximab-scFv library Related to G465R	F: ATGCGGCCCAGCCGGCCCAGGTTCAACTGAAACAGTCCGG<br>R: AAATATGCGGCCGCTTTCAGTTCCAGTTTCGTGCCGGCACCGAAAGT<br>Overlap-F1: CCTGGAATGGCTGGGTNNSATCNNSAGCGGCGGTAACACCNNSTACAATACCCCATTCA<br>Overlap-R1: TGAATGGGGTATTGTASNNGGTGTTACCGCCGCTSNNGATSNNACCCAGCCATTCCAGG

Supplementary References

1. S. Arena, B. Bellosillo, G. Siravegna, A. Martinez, I. Canadas, L. Lazzari, N. Ferruz, M. Russo, S. Misale, I. Gonzalez, M. Iglesias, E. Gavilan, G. Corti, S. Hobor, G. Crisafulli, M. Salido, J. Sanchez, A. Dalmases, J. Bellmunt, G. De Fabritiis, A. Rovira, F. Di Nicolantonio, J. Albanell, A. Bardelli, C. Montagut, Emergence of Multiple EGFR Extracellular Mutations during Cetuximab Treatment in Colorectal Cancer. *Clin Cancer Res* **21**, 2157-2166 (2015).
2. C. Montagut, A. Dalmases, B. Bellosillo, M. Crespo, S. Pairet, M. Iglesias, M. Salido, M. Gallen, S. Marsters, S. P. Tsai, A. Minoche, S. Seshagiri, S. Serrano, H. Himmelbauer, J. Bellmunt, A. Rovira, J. Settleman, F. Bosch, J. Albanell, Identification of a mutation in the extracellular domain of the Epidermal Growth Factor Receptor conferring cetuximab resistance in colorectal cancer. *Nat Med* **18**, 221-223 (2012).
3. Bertotti, A., Papp, E., Jones, S. et al. The genomic landscape of response to EGFR blockade in colorectal cancer. *Nature* **526**, 263–267 (2015).
4. F. Braig, M. Marz, A. Schieferdecker, A. Schulte, M. Voigt, A. Stein, T. Grob, M. Alawi, D. Indenbirken, M. Kriegs, E. Engel, U. Vanhoefer, A. Grundhoff, S. Loges, K. Riecken, B. Fehse, C. Bokemeyer, M. Binder, Epidermal growth factor receptor mutation mediates cross-resistance to panitumumab and cetuximab in gastrointestinal cancer. *Oncotarget* **6**, 12035-12047 (2015).

Supplementary Fig. 2a uncropped scans of gel

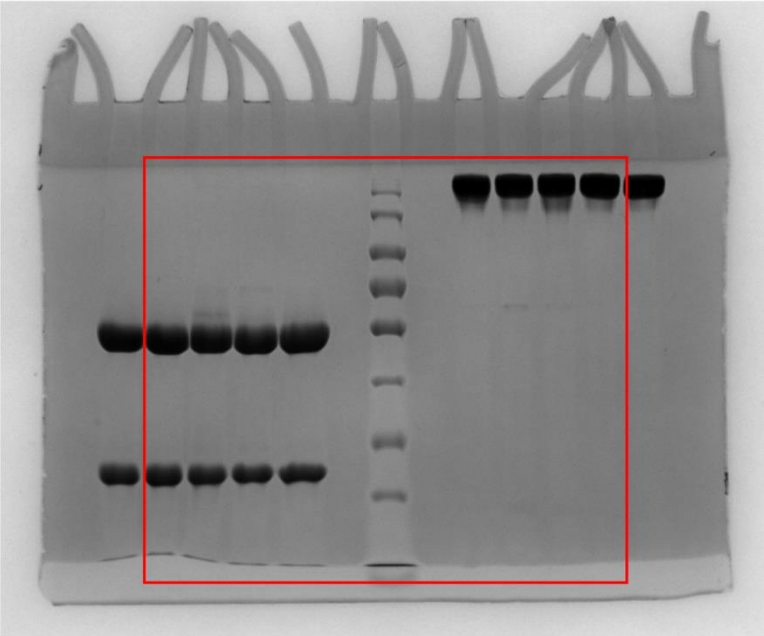

Supplementary Fig. 3 uncropped scans of gels

a

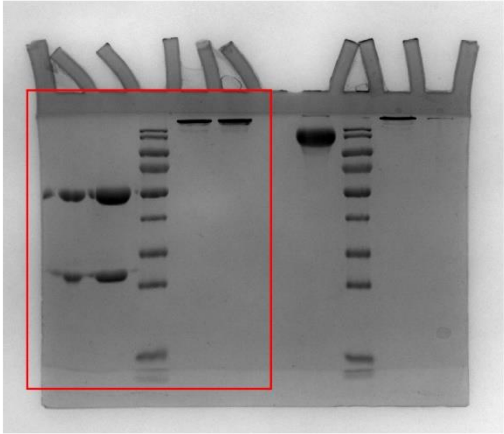

b

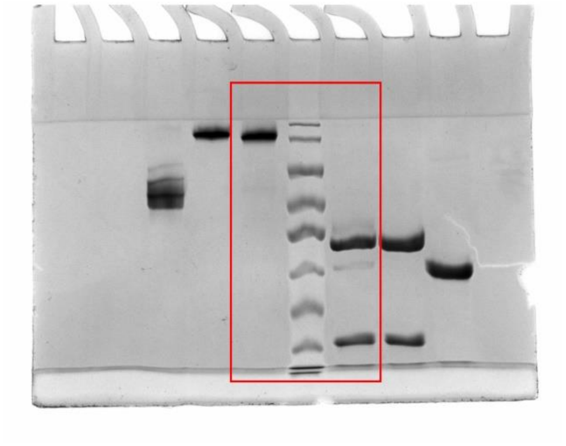

c

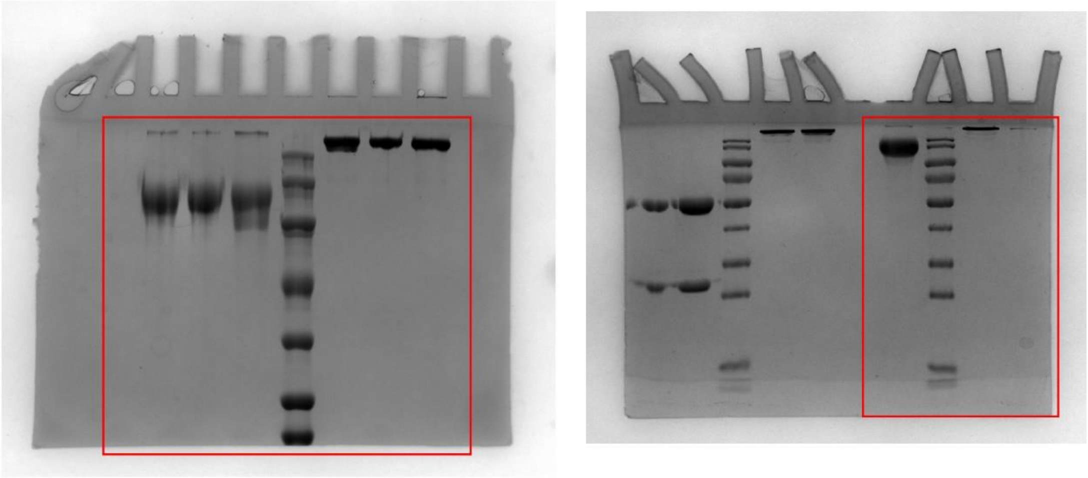

d

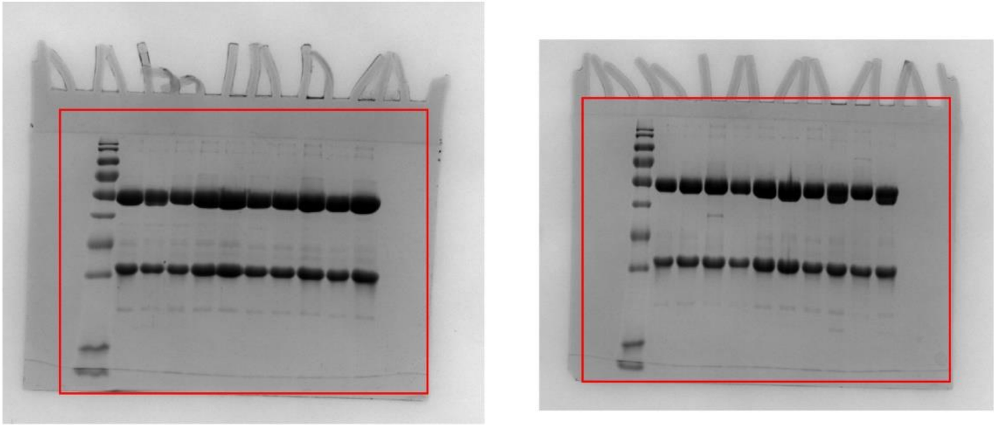

Supplement: Supplementary file 1 — Supplementary Information [file 41467_2022_32159_MOESM1_ESM.pdf]
