# Supplementary material for: Structure-guided and phage-assisted evolution of a therapeutic anti-EGFR antibody to reverse acquired resistance
Source: Nat Commun. 2022 Jul 30;13:4431. doi: 10.1038/s41467-022-32159-6 (PMC9338999; doi:10.1038/s41467-022-32159-6)
Supplement: Supplementary file 2 — Reporting Summary [file 41467_2022_32159_MOESM2_ESM.pdf]

nature research

Corresponding author(s): Shuqing Chen, Liqiang Pan
Last updated by author(s): Jun 29, 2022

Reporting Summary

Nature Research wishes to improve the reproducibility of the work that we publish. This form provides structure for consistency and transparency in reporting. For further information on Nature Research policies, see our Editorial Policies and the Editorial Policy Checklist.

Statistics

For all statistical analyses, confirm that the following items are present in the figure legend, table legend, main text, or Methods section.

n/a	Confirmed	
☐	☒	The exact sample size (*n*) for each experimental group/condition, given as a discrete number and unit of measurement
☐	☒	A statement on whether measurements were taken from distinct samples or whether the same sample was measured repeatedly
☐	☒	The statistical test(s) used AND whether they are one- or two-sided<br>*Only common tests should be described solely by name; describe more complex techniques in the Methods section.*
☒	☐	A description of all covariates tested
☐	☒	A description of any assumptions or corrections, such as tests of normality and adjustment for multiple comparisons
☐	☒	A full description of the statistical parameters including central tendency (e.g. means) or other basic estimates (e.g. regression coefficient) AND variation (e.g. standard deviation) or associated estimates of uncertainty (e.g. confidence intervals)
☐	☒	For null hypothesis testing, the test statistic (e.g. *F*, *t*, *r*) with confidence intervals, effect sizes, degrees of freedom and *P* value noted<br>*Give P values as exact values whenever suitable.*
☒	☐	For Bayesian analysis, information on the choice of priors and Markov chain Monte Carlo settings
☒	☐	For hierarchical and complex designs, identification of the appropriate level for tests and full reporting of outcomes
☒	☐	Estimates of effect sizes (e.g. Cohen's *d*, Pearson's *r*), indicating how they were calculated

Our web collection on statistics for biologists contains articles on many of the points above.

Software and code

Policy information about availability of computer code

Data collection	A Moflo Astrios EQ flow cytometer (Beckman, USA) and ACEA NovoCyteTM flow cytometer (ACEA Biosciences, USA) were used for flow cytometry data acquisition. ChemiDoc Touch Imaging System (Bio-Rad, USA) was used for gel scanning and Immunoblotting. A Synergy™ H1 microplate reader was used for absorbance detected in a 96 well plates for BCA protein quantification, ELISA assay, cell proliferation assay,ADCC and CDC assay. The Nanodrop 2000 Spectrophotometer (Thermo Fischer) was used to characterize proteins and nucleic acid. A NeoSPR-M100 (Neoline, Hangzhou, China) was used to acquire the surface plasmon resonance data. An ÄKTA pure system UPC-900 (GE Healthcare, PA, USA) was used for protein purification. Size exclusion date was acquired with the Agilent 1200 & EZChrom Elite software provided by Agilent Corporation. Fluorescence images were acquired with Olympus FV3000 confocal laser scanning microscope. A Cobas c311 analyzer(Roche Diagnostics GmbH, Germany) was used to test ALT, AST, BUN and Cr. LitesizerTM 500 (Anton paar, USA) was used for DLS data acquisition.
Data analysis	Pymol was used to analyze the cetuximab/EGFR crystal structure (Protein Data Bank: 1YY9). Rosetta platform was used for structure preparation and computational mutation scanning. The online server SWISS-MODEL was used to predict the 3D protein structures of S492R- or G465R-mutated EGFR, and cetuximab variants. GraphPad Prism (v8.4) was used for data analysis and curve-fitting. ACEANovoExpress was used for flow cytometry data analysis. Image J 1.52a (Wayne Rasband National Institutes of Health, USA)to quantify IHC staining data analysis.

For manuscripts utilizing custom algorithms or software that are central to the research but not yet described in published literature, software must be made available to editors and reviewers. We strongly encourage code deposition in a community repository (e.g. GitHub). See the Nature Research guidelines for submitting code & software for further information.

Data

Policy information about availability of data

All manuscripts must include a data availability statement. This statement should provide the following information, where applicable:

- Accession codes, unique identifiers, or web links for publicly available datasets
- A list of figures that have associated raw data
- A description of any restrictions on data availability

The structures of wild-type EGFR ECD/cetuximab Fab complex was obtained from the Protein Data Bank (PDB code: 1YY9) (https://www.rcsb.org/structure/1YY9). All data generated or analyzed during this study are included in this article and its Supplementary Information files. The uncropped gel or blot figures and original data underlying Figs. 1–7 and Supplementary Figs. 1–9 are provided as a Source Data file. Source data are provided with this paper. All the other data are available within the article and its Supplementary Information. Source data are provided with this paper.

Field-specific reporting

Please select the one below that is the best fit for your research. If you are not sure, read the appropriate sections before making your selection.

☒ Life sciences ☐ Behavioural & social sciences ☐ Ecological, evolutionary & environmental sciences

For a reference copy of the document with all sections, see nature.com/documents/nr-reporting-summary-flat.pdf

Life sciences study design

All studies must disclose on these points even when the disclosure is negative.

Sample size	For in vitro experiments, at least three biologically independent experiments were performed for all experiments unless otherwise stated. No statistical method was used to predetermine sample size. Such sample sizes are typical for the in vitro experiments and sufficient for a statistical analysis. For in vivo experiments, a sample size of n = 5 mice per group were used, which is sufficient to generate statistically significant results. No statistical method was used to predetermine sample size. The sample size for animal studies were chosen based on prior experience with the same experimental design.
Data exclusions	There were no data exclusions from the data provided.
Replication	All biological data were confirmed with multiple replicates as noted in the methods and figure legends.
Randomization	No specific randomization was done for in vitro experiments or assays. For in vivo anti-tumor experiments, tumor-bearing mice in different treatment groups were randomly divided into different experimental groups and housed under standard conditions.
Blinding	No blinding was performed in vitro and in vivo experiments in order to make comparisons between specific treatments. In addition, We performed experiments in a non-blinded manner, since the experimental design was complicated and the researchers were limited.

Behavioural & social sciences study design

All studies must disclose on these points even when the disclosure is negative.

Study description	*Briefly describe the study type including whether data are quantitative, qualitative, or mixed-methods (e.g. qualitative cross-sectional, quantitative experimental, mixed-methods case study).*
Research sample	*State the research sample (e.g. Harvard university undergraduates, villagers in rural India) and provide relevant demographic information (e.g. age, sex) and indicate whether the sample is representative. Provide a rationale for the study sample chosen. For studies involving existing datasets, please describe the dataset and source.*
Sampling strategy	*Describe the sampling procedure (e.g. random, snowball, stratified, convenience). Describe the statistical methods that were used to predetermine sample size OR if no sample-size calculation was performed, describe how sample sizes were chosen and provide a rationale for why these sample sizes are sufficient. For qualitative data, please indicate whether data saturation was considered, and what criteria were used to decide that no further sampling was needed.*
Data collection	*Provide details about the data collection procedure, including the instruments or devices used to record the data (e.g. pen and paper, computer, eye tracker, video or audio equipment) whether anyone was present besides the participant(s) and the researcher, and whether the researcher was blind to experimental condition and/or the study hypothesis during data collection.*
Timing	*Indicate the start and stop dates of data collection. If there is a gap between collection periods, state the dates for each sample cohort.*
Data exclusions	*If no data were excluded from the analyses, state so OR if data were excluded, provide the exact number of exclusions and the rationale behind them, indicating whether exclusion criteria were pre-established.*

Non-participation	*State how many participants dropped out/declined participation and the reason(s) given OR provide response rate OR state that no participants dropped out/declined participation.*
Randomization	*If participants were not allocated into experimental groups, state so OR describe how participants were allocated to groups, and if allocation was not random, describe how covariates were controlled.*

Ecological, evolutionary & environmental sciences study design

All studies must disclose on these points even when the disclosure is negative.

Study description	*Briefly describe the study. For quantitative data include treatment factors and interactions, design structure (e.g. factorial, nested, hierarchical), nature and number of experimental units and replicates.*
Research sample	*Describe the research sample (e.g. a group of tagged Passer domesticus, all Stenocereus thurberi within Organ Pipe Cactus National Monument), and provide a rationale for the sample choice. When relevant, describe the organism taxa, source, sex, age range and any manipulations. State what population the sample is meant to represent when applicable. For studies involving existing datasets, describe the data and its source.*
Sampling strategy	*Note the sampling procedure. Describe the statistical methods that were used to predetermine sample size OR if no sample-size calculation was performed, describe how sample sizes were chosen and provide a rationale for why these sample sizes are sufficient.*
Data collection	*Describe the data collection procedure, including who recorded the data and how.*
Timing and spatial scale	*Indicate the start and stop dates of data collection, noting the frequency and periodicity of sampling and providing a rationale for these choices. If there is a gap between collection periods, state the dates for each sample cohort. Specify the spatial scale from which the data are taken*
Data exclusions	*If no data were excluded from the analyses, state so OR if data were excluded, describe the exclusions and the rationale behind them, indicating whether exclusion criteria were pre-established.*
Reproducibility	*Describe the measures taken to verify the reproducibility of experimental findings. For each experiment, note whether any attempts to repeat the experiment failed OR state that all attempts to repeat the experiment were successful.*
Randomization	*Describe how samples/organisms/participants were allocated into groups. If allocation was not random, describe how covariates were controlled. If this is not relevant to your study, explain why.*
Blinding	*Describe the extent of blinding used during data acquisition and analysis. If blinding was not possible, describe why OR explain why blinding was not relevant to your study.*

Did the study involve field work? ☐ Yes ☐ No

Field work, collection and transport

Field conditions	*Describe the study conditions for field work, providing relevant parameters (e.g. temperature, rainfall).*
Location	*State the location of the sampling or experiment, providing relevant parameters (e.g. latitude and longitude, elevation, water depth).*
Access & import/export	*Describe the efforts you have made to access habitats and to collect and import/export your samples in a responsible manner and in compliance with local, national and international laws, noting any permits that were obtained (give the name of the issuing authority, the date of issue, and any identifying information).*
Disturbance	*Describe any disturbance caused by the study and how it was minimized.*

Reporting for specific materials, systems and methods

We require information from authors about some types of materials, experimental systems and methods used in many studies. Here, indicate whether each material, system or method listed is relevant to your study. If you are not sure if a list item applies to your research, read the appropriate section before selecting a response.

Materials & experimental systems

n/a	Involved in the study	
	☒	Antibodies
	☒	Eukaryotic cell lines
☒		Palaeontology and archaeology
	☒	Animals and other organisms
☒		Human research participants
☒		Clinical data
☒		Dual use research of concern

Methods

n/a	Involved in the study	
☒		ChIP-seq
	☒	Flow cytometry
☒		MRI-based neuroimaging

Antibodies

Antibodies used

Mouse anti-Flag tag antibody (Genscript, China; A00187), 1:500
FITC-conjugated goat anti-mouse IgG (H+L) (Beyotime, China, A0568), 1:500
HRP-conjugated anti-E tag antibody (Abcam, Cambridge, MA, USA, Ab3415), 1:1000
FITC-conjugated goat anti-human IgG (H+L) (Beyotime, China, A0556), 1:500
HRP-conjugated polyclonal goat anti-human kappa light chain antibody (Thermo Fisher Scientific, USA, A18853), 1:1000
Rabbit anti-EGFR antibody (Cell Signaling Technology, Danvers, USA, 4267T), 1:1000
Rabbit anti-EGFR phosphorylated Tyr1068 antibody (Cell Signaling Technology, Danvers, USA,3777T), 1:1000
Rabbit anti-Akt antibody (Cell Signaling Technology, Danvers, USA, 4691T), 1:1000
Rabbit anti-Akt phosphorylated Ser473 antibody (Cell Signaling Technology, Danvers, USA, 4060T), 1:1000
Rabbit anti-Erk antibody (Cell Signaling Technology, Danvers, USA, 4695T), 1:1000
Rabbit anti-Erk phosphorylated Thr202/Tyr204 antibody (Cell Signaling Technology, Danvers, USA,4376S), 1:1000
HRP-conjugated goat anti-rabbit IgG (H+L) (Beyotime, China, A0208), 1:2000
HRP-conjugated goat anti-rabbit IgG (H+L) (Zhongshan Goldbridge Biotechnology, PV6001), 100 μL per sample
Rabbit polyclonal anti-EGFR (Beyotime,China, AF5153), 1:200
Rabbit polyclonal anti-Ki-67 (Abcam, ab15580),1:500

Validation

All commercially available antibodies were validated by the vendor.
Links for each antibody used are:
Mouse anti-Flag tag antibody (https://www.genscript.com.cn/antibody/A00187-THE_DYKDDDDK_Tag_Antibody_mAb_Mouse.html)
FITC-conjugated goat anti-mouse IgG (H+L) (https://www.beyotime.com/product/A0568.htm)
HRP-conjugated anti-E tag antibody (https://www.abcam.cn/hrp-e-tag-antibody-ab3415.html)
FITC-conjugated goat anti-human IgG (H+L) (https://www.beyotime.com/product/A0556.htm)
HRP-conjugated goat anti-rabbit IgG (H+L)(https://www.beyotime.com/product/A0208.htm)
HRP-conjugated polyclonal goat anti-human kappa light chain antibody(https://www.thermofisher.com/cn/zh/antibody/product/Goat-anti-Human-Kappa-Light-Chain-Secondary-Antibody-Polyclonal/A18853)
Rabbit anti-EGFR antibody (https://www.cellsignal.cn/products/primary-antibodies/egf-receptor-d38b1-xp-rabbit-mab/4267?site-search-type=Products&N=4294956287&Ntt=egfr&fromPage=plp)
Rabbit anti-EGFR phosphorylated Tyr1068 antibody (https://www.cellsignal.cn/products/primary-antibodies/phospho-egf-receptor-tyr1068-d7a5-xp-rabbit-mab/3777?site-search-type=Products&N=4294956287&Ntt=egfr&fromPage=plp)
Rabbit anti-Akt antibody (https://www.cellsignal.cn/products/primary-antibodies/akt-pan-c67e7-rabbit-mab/4691?site-search-type=Products&N=4294956287&Ntt=akt&fromPage=plp)
Rabbit anti-Akt phosphorylated Ser473 antibody (https://www.cellsignal.cn/products/primary-antibodies/phospho-akt-ser473-d9e-xp-rabbit-mab/4060?site-search-type=Products&N=4294956287&Ntt=akt&fromPage=plp)
Rabbit anti-Erk antibody (https://www.cellsignal.cn/products/primary-antibodies/p44-42-mapk-erk1-2-137f5-rabbit-mab/4695?site-search-type=Products&N=4294956287&Ntt=erk&fromPage=plp)
Rabbit anti-Erk phosphorylated Thr202/Tyr204 antibody (https://www.cellsignal.cn/products/primary-antibodies/phospho-p44-42-mapk-erk1-2-thr202-tyr204-20g11-rabbit-mab/4376?site-search-type=Products&N=4294956287&Ntt=erk&fromPage=plp).
Rabbit polyclonal anti-EGFR (https://www.beyotime.com/product/AF5153.htm)
Rabbit polyclonal anti-Ki-67 (https://www.abcam.cn/ki67-antibody-ab15580.html)

Eukaryotic cell lines

Policy information about cell lines

Cell line source(s)

NIH3T3 mouse embryonic fibroblast cells were purchased from the Cell Bank of the Chinese Academy of Sciences (Shanghai, China). HEK293T and HEK293F cells was a gift from Prof Dr. Linqi Zhang (Tsinghua University, China) which was originally from ATCC. SW48 and COLO320DM cells were purchased from Cobioer Biosciences (Nanjing, China).

Authentication

Cell lines have not been subjected to additional authentication.

Mycoplasma contamination

Cells lines were tested regularly for mycoplasma contamination and were negative.

Commonly misidentified lines (See ICLAC register)	No commonly misidentified cell line was used.

Palaeontology and Archaeology

Specimen provenance	*Provide provenance information for specimens and describe permits that were obtained for the work (including the name of the issuing authority, the date of issue, and any identifying information).*
Specimen deposition	*Indicate where the specimens have been deposited to permit free access by other researchers.*
Dating methods	*If new dates are provided, describe how they were obtained (e.g. collection, storage, sample pretreatment and measurement), where they were obtained (i.e. lab name), the calibration program and the protocol for quality assurance OR state that no new dates are provided.*

☐ Tick this box to confirm that the raw and calibrated dates are available in the paper or in Supplementary Information.

Ethics oversight	*Identify the organization(s) that approved or provided guidance on the study protocol, OR state that no ethical approval or guidance was required and explain why not.*

Note that full information on the approval of the study protocol must also be provided in the manuscript.

Animals and other organisms

Policy information about studies involving animals; ARRIVE guidelines recommended for reporting animal research

Laboratory animals	BALB/c nude mice (6- to 8-weeks old, male, body weight 20–30 g) were purchased from Shanghai SLAC Laboratory Animal Co., Ltd. (Shanghai, China) and housed in groups of 5 mice in an individually ventilated cage (IVC) in a 12:12 light-dark cycle (08:30–20:30 light; 20:30–8:30 dark). The ambient temperature was 22 ± 2°C with 50–60% relative humidity.
Wild animals	No wild animals were involved in this study.
Field-collected samples	The study did not involve field-collected samples.
Ethics oversight	Experiments were carried out according to the National Institutes of Health (United States) Guide for the Care and Use of Laboratory Animals. All the animal work was performed in accordance with the protocol approved by the Committee on the Ethics of Animal Experiments of Zhejiang University (Hangzhou, China).

Note that full information on the approval of the study protocol must also be provided in the manuscript.

Human research participants

Policy information about studies involving human research participants

Population characteristics	*Describe the covariate-relevant population characteristics of the human research participants (e.g. age, gender, genotypic information, past and current diagnosis and treatment categories). If you filled out the behavioural & social sciences study design questions and have nothing to add here, write "See above."*
Recruitment	*Describe how participants were recruited. Outline any potential self-selection bias or other biases that may be present and how these are likely to impact results.*
Ethics oversight	*Identify the organization(s) that approved the study protocol.*

Note that full information on the approval of the study protocol must also be provided in the manuscript.

Clinical data

Policy information about clinical studies

All manuscripts should comply with the ICMJEguidelines for publication of clinical research and a completedCONSORT checklist must be included with all submissions.

Clinical trial registration	*Provide the trial registration number from ClinicalTrials.gov or an equivalent agency.*
Study protocol	*Note where the full trial protocol can be accessed OR if not available, explain why.*
Data collection	*Describe the settings and locales of data collection, noting the time periods of recruitment and data collection.*
Outcomes	*Describe how you pre-defined primary and secondary outcome measures and how you assessed these measures.*

Dual use research of concern

Policy information about dual use research of concern

Hazards

Could the accidental, deliberate or reckless misuse of agents or technologies generated in the work, or the application of information presented in the manuscript, pose a threat to:

No	Yes	
☐	☐	Public health
☐	☐	National security
☐	☐	Crops and/or livestock
☐	☐	Ecosystems
☐	☐	Any other significant area

Experiments of concern

Does the work involve any of these experiments of concern:

No	Yes	
☐	☐	Demonstrate how to render a vaccine ineffective
☐	☐	Confer resistance to therapeutically useful antibiotics or antiviral agents
☐	☐	Enhance the virulence of a pathogen or render a nonpathogen virulent
☐	☐	Increase transmissibility of a pathogen
☐	☐	Alter the host range of a pathogen
☐	☐	Enable evasion of diagnostic/detection modalities
☐	☐	Enable the weaponization of a biological agent or toxin
☐	☐	Any other potentially harmful combination of experiments and agents

ChIP-seq

Data deposition

☐ Confirm that both raw and final processed data have been deposited in a public database such as GEO.

☐ Confirm that you have deposited or provided access to graph files (e.g. BED files) for the called peaks.

Data access links<br>*May remain private before publication.*	*For "Initial submission" or "Revised version" documents, provide reviewer access links. For your "Final submission" document, provide a link to the deposited data.*
Files in database submission	*Provide a list of all files available in the database submission.*
Genome browser session<br>(e.g. UCSC)	*Provide a link to an anonymized genome browser session for "Initial submission" and "Revised version" documents only, to enable peer review. Write "no longer applicable" for "Final submission" documents.*

Methodology

Replicates	*Describe the experimental replicates, specifying number, type and replicate agreement.*
Sequencing depth	*Describe the sequencing depth for each experiment, providing the total number of reads, uniquely mapped reads, length of reads and whether they were paired- or single-end.*
Antibodies	*Describe the antibodies used for the ChIP-seq experiments; as applicable, provide supplier name, catalog number, clone name, and lot number.*
Peak calling parameters	*Specify the command line program and parameters used for read mapping and peak calling, including the ChIP, control and index files used.*
Data quality	*Describe the methods used to ensure data quality in full detail, including how many peaks are at FDR 5% and above 5-fold enrichment.*
Software	*Describe the software used to collect and analyze the ChIP-seq data. For custom code that has been deposited into a community repository, provide accession details.*

Flow Cytometry

Plots

Confirm that:

- [x] The axis labels state the marker and fluorochrome used (e.g. CD4-FITC).
- [x] The axis scales are clearly visible. Include numbers along axes only for bottom left plot of group (a 'group' is an analysis of identical markers).
- [x] All plots are contour plots with outliers or pseudocolor plots.
- [x] A numerical value for number of cells or percentage (with statistics) is provided.

Methodology

Sample preparation	To determine therapeutic antibody binding affinity with cell lines, wild-type or mutant EGFR (i.e., S492R, I491M, K489E, K467T, G465R, G465E, S464L, I462R, R451C, S442R, V441D) expressing NIH3T3 cells were harvested by trypsinization and washed twice with cold PBS (pH 7.4). Each cell line was stained with cetuximab or panitumumab (three 10-fold serial dilutions from 100 nmol/L, 30min) on ice, followed by washing with ice cold PBS (pH 7.4) twice and treatment with FITC-conjugated goat anti-human IgG (H+L) secondary antibody (Beyotime, China) at a 1:500 dilution in PBS (pH 7.4) for 30min on ice. Samples were washed and resuspended in ice cold PBS (pH 7.4) before analysis via ACEA NovoCyteTM flow cytometer (ACEA Biosciences, USA). Percentages of monoclonal antibody stained cells/total EGFR positive cells were determined. Mean fluorescent intensity (MFI) of immune-stained cells was also determined by flow cytometry.
Instrument	ACEA NovoCyteTM flow cytometer (ACEA Biosciences, USA)
Software	Data analysis was performed using the ACEANovoExpress
Cell population abundance	N/A - cell sorting was not performed.
Gating strategy	We gated upon the population of single cells based on FSC and SSC values.

- [x] Tick this box to confirm that a figure exemplifying the gating strategy is provided in the Supplementary Information.

Magnetic resonance imaging

Experimental design

Design type	*Indicate task or resting state; event-related or block design.*
Design specifications	*Specify the number of blocks, trials or experimental units per session and/or subject, and specify the length of each trial or block (if trials are blocked) and interval between trials.*
Behavioral performance measures	*State number and/or type of variables recorded (e.g. correct button press, response time) and what statistics were used to establish that the subjects were performing the task as expected (e.g. mean, range, and/or standard deviation across subjects).*

Acquisition

Imaging type(s)	*Specify: functional, structural, diffusion, perfusion.*
Field strength	*Specify in Tesla*
Sequence & imaging parameters	*Specify the pulse sequence type (gradient echo, spin echo, etc.), imaging type (EPI, spiral, etc.), field of view, matrix size, slice thickness, orientation and TE/TR/flip angle.*
Area of acquisition	*State whether a whole brain scan was used OR define the area of acquisition, describing how the region was determined.*

Diffusion MRI ☐ Used ☐ Not used

Preprocessing

Preprocessing software	*Provide detail on software version and revision number and on specific parameters (model/functions, brain extraction, segmentation, smoothing kernel size, etc.).*
Normalization	*If data were normalized/standardized, describe the approach(es): specify linear or non-linear and define image types used for transformation OR indicate that data were not normalized and explain rationale for lack of normalization.*
Normalization template	*Describe the template used for normalization/transformation, specifying subject space or group standardized space (e.g. original Talairach, MNI305, ICBM152) OR indicate that the data were not normalized.*

Noise and artifact removal	*Describe your procedure(s) for artifact and structured noise removal, specifying motion parameters, tissue signals and physiological signals (heart rate, respiration).*
Volume censoring	*Define your software and/or method and criteria for volume censoring, and state the extent of such censoring.*

Statistical modeling & inference

Model type and settings	*Specify type (mass univariate, multivariate, RSA, predictive, etc.) and describe essential details of the model at the first and second levels (e.g. fixed, random or mixed effects; drift or auto-correlation).*
Effect(s) tested	*Define precise effect in terms of the task or stimulus conditions instead of psychological concepts and indicate whether ANOVA or factorial designs were used.*

Specify type of analysis: ☐ Whole brain ☐ ROI-based ☐ Both

Statistic type for inference<br>(See Eklund et al. 2016)	*Specify voxel-wise or cluster-wise and report all relevant parameters for cluster-wise methods.*
Correction	*Describe the type of correction and how it is obtained for multiple comparisons (e.g. FWE, FDR, permutation or Monte Carlo).*

Models & analysis

n/a	Involved in the study
☐	☐ Functional and/or effective connectivity
☐	☐ Graph analysis
☐	☐ Multivariate modeling or predictive analysis

Functional and/or effective connectivity	*Report the measures of dependence used and the model details (e.g. Pearson correlation, partial correlation, mutual information).*
Graph analysis	*Report the dependent variable and connectivity measure, specifying weighted graph or binarized graph, subject- or group-level, and the global and/or node summaries used (e.g. clustering coefficient, efficiency, etc.).*
Multivariate modeling and predictive analysis	*Specify independent variables, features extraction and dimension reduction, model, training and evaluation metrics.*
