# Supplementary material for: Structure-guided and phage-assisted evolution of a therapeutic anti-EGFR antibody to reverse acquired resistance
Source: Nat Commun. 2022 Jul 30;13:4431. doi: 10.1038/s41467-022-32159-6 (PMC9338999; doi:10.1038/s41467-022-32159-6)
Supplement: Supplementary file 3 — Description of Additional Supplementary Files [file 41467_2022_32159_MOESM3_ESM.pdf]

File name: Supplementary Data 1

Description: The $\Delta\Delta G$ values for Cluster 1 of $EGFR^{S492R}$/ $Cetuximab^{Mut}$.

File name: Supplementary Data 2

Description: The $\Delta\Delta G$ values for Cluster 2 of $EGFR^{S492R}$/ $Cetuximab^{Mut}$.

File name: Supplementary Data 3

Description: The $\Delta\Delta G$ values for Cluster 3 of $EGFR^{S492R}$/ $Cetuximab^{Mut}$.

File name: Supplementary Data 4

Description: The $\Delta\Delta G$ values for Cluster 1 of $EGFR^{G465R}$/ $Cetuximab^{Mut}$.

File name: Supplementary Data 5

Description: The $\Delta\Delta G$ values for Cluster 2 of $EGFR^{G465R}$/ $Cetuximab^{Mut}$.

File name: Supplementary Data 6

Description: The $\Delta\Delta G$ values for Cluster 3 of $EGFR^{G465R}$/ $Cetuximab^{Mut}$.
